# Transcriptional patterns of cancer-related genes in primary and metastatic tumours revealed by machine learning

**DOI:** 10.1186/s12915-025-02339-z

**Published:** 2025-08-07

**Authors:** Faeze Keshavarz-Rahaghi, Erin Pleasance, Steven J. M. Jones

**Affiliations:** 1 Canada’s Michael Smith Genome Sciences Centre at BC Cancer, Vancouver, BC Canada; 2 Bioinformatics Graduate Program, University of British Columbia, Vancouver, BC Canada; 3 Department of Medical Genetics, University of British Columbia, Vancouver, BC Canada; 4 Department of Molecular Biology and Biochemistry, Simon Fraser University, Burnaby, BC Canada

**Keywords:** Transcriptome, Machine learning, Cancer, Random Forest

## Abstract

**Background:**

A key to understanding cancer is to determine the impact on the cellular pathways caused by the repertoire of DNA changes accrued in a cancer cell. Exploring the interactions between genomic aberrations and the expressed transcriptome can not only improve our understanding of the disease but also identify potential therapeutic approaches.

**Results:**

Using random forest models, we successfully identified transcriptional patterns associated with the loss of wild-type activity in cancer-related genes across various tumour types. While genes like *TP53* and *CDKN2A* exhibited unique pan-cancer transcriptional patterns, others like *ATRX*, *BRAF*, and *NRAS* showed tumour-type-specific expression patterns. We also observed that genes like *AR* and *ERBB4* did not lead to strong detectable patterns in the transcriptome when disrupted. Our investigation has also led to the identification of genes highly associated with transcriptional patterns. For instance, *DRG2* emerged as the top contributor in classification of *ATRX* alterations in lower-grade gliomas and was significantly downregulated in *ATRX* mutant tumours. Additionally, transcriptional features important in classification of *PTEN* aberrations, such as *CDCA8*, *AURKA*, and *CDC20*, were found to be closely related to *PTEN* function.

**Conclusions:**

Our findings demonstrate the utility of machine learning in interpretation of cancer genomic data and provide new avenues for development of targeted therapies tailored to individual patients with cancer. Our analysis on the transcriptome revealed genes with expression levels strongly correlated with alterations in cancer-related genes. Additionally, we identified *AURKA* inhibitors as potential therapeutic option for tumours with alterations in tumour suppressors like *FBXW7* or *NSD1*.

**Supplementary Information:**

The online version contains supplementary material available at 10.1186/s12915-025-02339-z.

## Background

Cancer remains a leading cause of mortality worldwide, with treatment efficacy and adverse effects varying among individual patients [1, 2]. Cancer drivers are frequently mutated genes, playing important roles in cancer initiation, progression, and maintenance [3–6]. Incorporating molecular data into clinical practice enables personalized therapies and improves patient outcomes [1, 2, 7]. Pivotal initiatives including The Cancer Genome Atlas (TCGA) and the British Columbia (BC) Cancer Agency’s Personalized Oncogenomics (POG) program have sequenced hundreds to thousands of tumour samples, generating genome sequences and gene expression profiles [7, 8].

While DNA sequencing is widely used in clinical settings, and most regulatory-approved targeted treatments by the United States Food and Drug Administration are based on genomic alterations [9–11], only a subset of tumours harbour actionable genomic alterations and therapies guided solely by DNA sequencing do not always lead to response. [2, 12]. Furthermore, a significant proportion of mutations identified through whole-genome sequencing occur in regions not transcribed as RNA for which it is challenging to infer role in tumour function [3]. Investigating the transcriptome aids in comprehending the effects of genetic aberrations on cellular pathways [1, 13, 14]. The dynamic nature of the transcriptome which is influenced by cell type, cellular state, regulatory mechanisms, and other factors provides insights into molecular changes in cancer [1]. Gene expression data has proven valuable for drug sensitivity prediction and detection of cancer subtypes with unique clinical outcomes [15–19].

Interpreting the impact of different types of variations on gene function is feasible through investigating the transcriptome [1]. Single-nucleotide variants (SNVs), small insertions/deletions (INDELs), copy number alterations (CNAs), and structural variants (SVs) are prevalent genomic aberrations which can be associated with transcriptional patterns and combined to predict tumour features [3, 9, 13, 14, 20]. Machine learning (ML) methods have demonstrated success in analysing pan-cancer genome-wide expression data for diverse applications, including predicting tumour types, identifying molecular signatures of metastasis, detecting pathway activation, and uncovering prognostic genes [21–24]. These methods have identified transcriptional signatures of disease with significance in prognosis and treatment [13, 14, 16]. However, prior studies were constrained to a limited number of genes and/or tumour types, while a broader gene list was explored only when the primary focus was on distinguishing cancer state from normal [25]. Consequently, comprehensive research regarding the impact of gene alterations on the transcriptome remains lacking.

In this study, we used ML to determine whether genetic aberrations in cancer driver genes generate specific and detectable transcriptional changes unique to those genes. Through an analysis of pan-cancer whole transcriptome data, we identified transcriptional changes associating with alterations in specific cancer-related genes, either spanning multiple tumour types or specific to a particular tumour type. Using random forest (RF) models, we pinpointed gene transcripts contributing to these patterns and examined their relationships with the driver genes under investigation. These findings provide insights into oncogenic mechanisms and have the potential to help identify active and targetable cellular pathways in primary and metastatic tumours.

## Results

### Expression profiles from tumour cohorts

To explore transcriptional patterns related to tumour mutations, we collated 8726 tumour samples from TCGA and 608 from POG studies, a total of 9334 patients, which include complete DNA mutation and RNA expression data. 56,645 overlapping coding and non-coding genes were identified. PCA plots revealed clustering of samples from the two studies, despite being from various tumour types and a mix of primary and metastatic tumours (Additional file 1: Figures S1-S3). Given the absence of distinct systematic separation between the TCGA and POG datasets, the samples were merged for downstream classification analyses.

Frequently mutated and cancer driver genes were reviewed [3, 5, 6], to select 50 genes as the focus of this study (Additional file 1: Table S1). Using somatic and germline SNV/INDEL data, samples were categorized into three groups: with “impactful” mutations, including nonsense and missense events, with “non-impactful” mutations, including synonymous and UTR events, and with wild-type gene copies.

### Random Forest classification improved by combining SNV/INDEL and copy number data

To evaluate whether transcriptional patterns associated with SNVs/INDELs in the genes of interest exist, ML models were used to classify tumour transcriptomes based on mutational status of these genes. RF, Support Vector Machine (SVM), and Neural Network (NN) models were tested on mutational status of *TP53* (Additional file 1: Figure S4). Among these, RF outperformed the others and surpassed the performance of the XGBoost model previously used by Zhang et al. to classify p53 pathway activity [26]. Additionally, our prior in-depth analysis on *TP53* validated the suitability of RF models for this type of task [13]. Thus, RFs were used for all subsequent classification tasks.

**Fig. 1:**
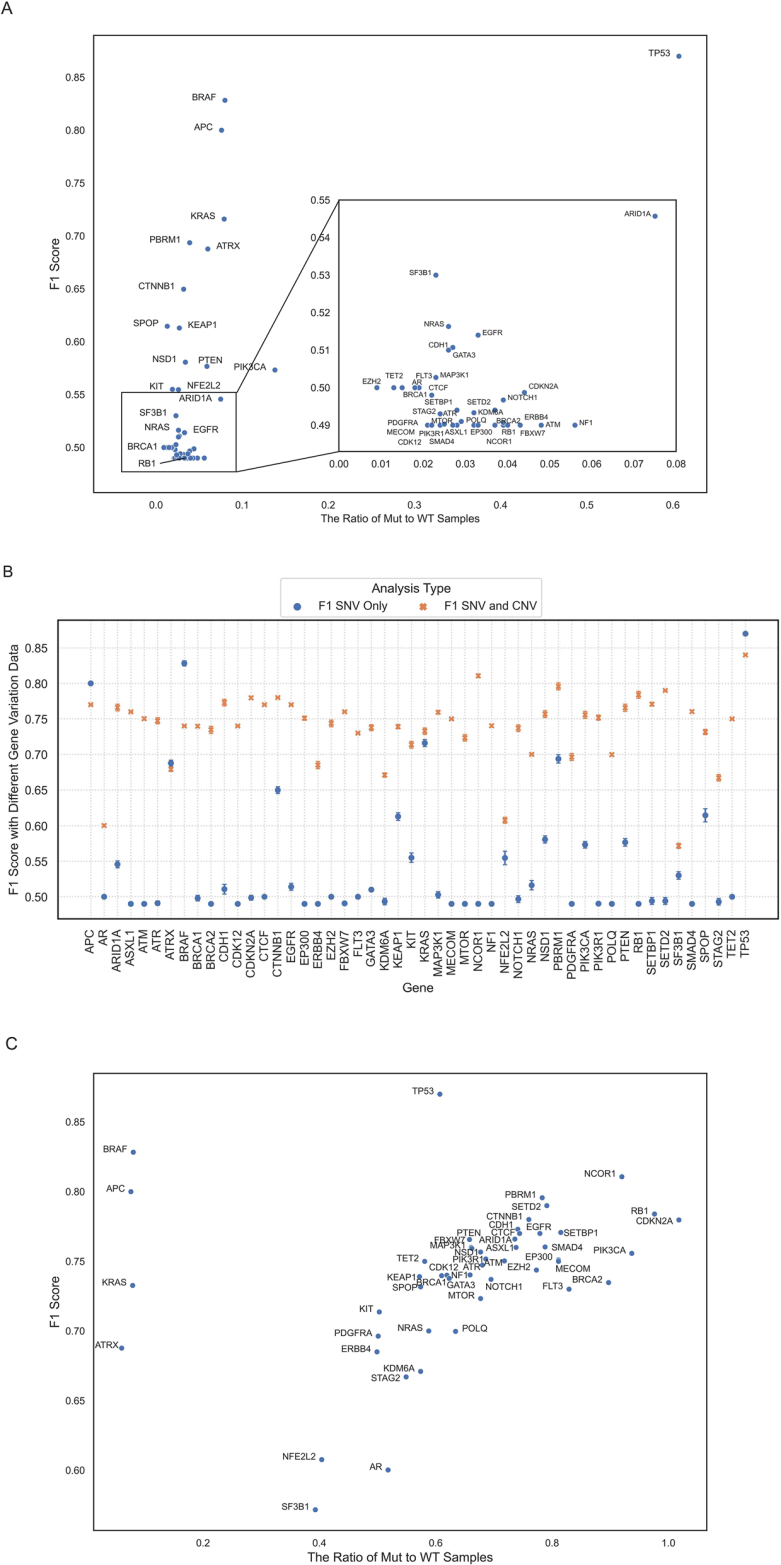
F1 score of classification **A** when only samples with SNVs/INDELs were labeled as mutant versus the ratio of samples containing mutations over samples with wild-type gene copies, **B** with different combinations of gene alterations. Blue circles represent results when only samples with SNVs/INDELs were labeled as mutant and orange crosses show the results when samples with either SNVs/INDELs or CNAs were labeled as mutant, **C** when samples with either SNVs/INDELs or CNAs were labeled as mutant versus the ratio of samples containing alterations over samples with wild-type gene copies

Only samples harbouring “impactful” mutations and wild-type copies were included. Five-fold CV analyses were conducted, and F1 scores were calculated (Fig. 1A). For many genes, small variants were found to be relatively infrequent, which contributed to class imbalance and adversely affected the model’s overall performance as evidenced by low F1 scores. We therefore incorporated other types of somatic variation, CNA and SV data, into the analysis. Four different sets of analyses were conducted: the first focused solely on the impact of SNVs and INDELs, while the other three examined combinations of SNVs/INDELs with CNAs, SNVs/INDELs with SVs, and finally SNVs/INDELs with both CNAs and SVs.

Average F1 scores indicated that adding copy number data significantly improved performance, increasing the F1 score by ~ 19.3% on average (Additional file 1: Figure S5). In contrast, inclusion of structural variant data only increased the F1 score by ~ 0.1%. The limited effect of SVs might be attributed to the small number of samples carrying such variations in our data, and given the difficulty in confirming the impact of SVs on the genes of interest which can lead to ambiguity during training, SV data was excluded from further analysis.

For most genes, the inclusion of CNAs enhanced the model’s performance (Fig. 1B). Consequently, CNAs were incorporated into downstream analysis for all genes except *APC*, *ATRX*, *BRAF*, *KRAS*, and *TP53*, for which performance was not improved. Robustness of these findings was confirmed with consistent results on an independent 10% test set (Additional file 1: Figure S6). Incorporating CNAs not only aligns with biological understanding of different ways genes are impacted in cancer [27], but also helps mitigate the class imbalance problem by improving the mutant to wild-type ratio (Fig. 1C).

### Tumour type-specific analysis and class imbalance at the tumour type level

**Fig. 2:**
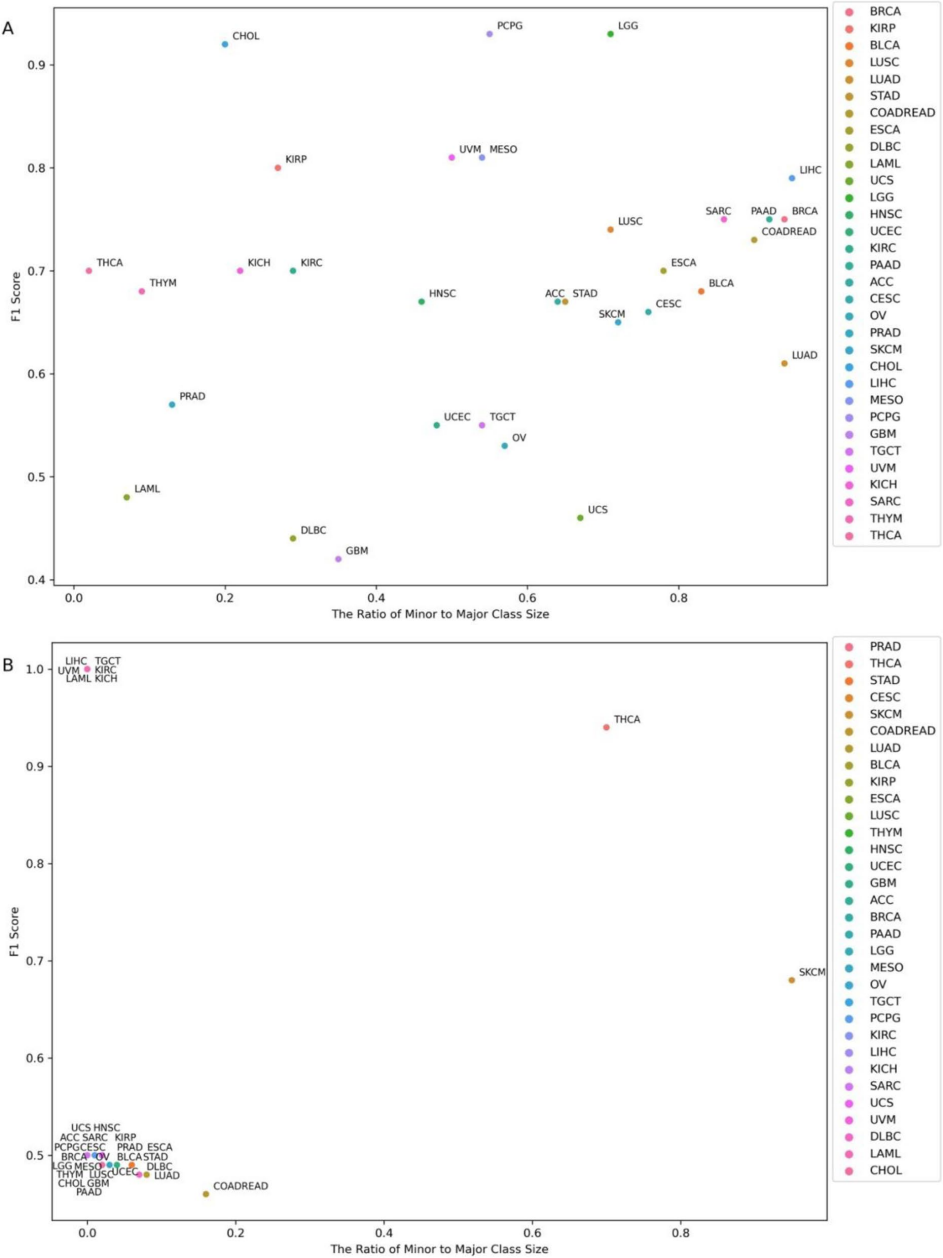
F1 scores for classification of samples based on **A** *ARID1A* and **B** *BRAF* alterations against the ratio of minor to major group sizes (with the minor group being the smaller of mutant or wild-type sample sets) across 33 TCGA tumour types (COAD and READ are combined). TCGA tumour types: ACC = Adrenocortical carcinoma, BLCA = Bladder Urothelial Carcinoma, BRCA = Breast invasive carcinoma, CESC = Cervical squamous cell carcinoma and endocervical adenocarcinoma, CHOL = Cholangiocarcinoma, COAD = Colon adenocarcinoma, DLBC = Lymphoid Neoplasm Diffuse Large B-cell Lymphoma, ESCA = Esophageal carcinoma, GBM = Glioblastoma multiforme, HNSC = Head and Neck squamous cell carcinoma, KICH = Kidney Chromophobe, KIRC = Kidney renal clear cell carcinoma, KIRP = Kidney renal papillary cell carcinoma, LAML = Acute Myeloid Leukaemia, LGG = Brain Lower Grade Glioma, LIHC = Liver hepatocellular carcinoma, LUAD = Lung adenocarcinoma, LUSC = Lung squamous cell carcinoma, MESO = Mesothelioma, OV = Ovarian serous cystadenocarcinoma, PAAD = Pancreatic adenocarcinoma, PCPG = Pheochromocytoma and Paraganglioma, PRAD = Prostate adenocarcinoma, READ = Rectum adenocarcinoma, SARC = Sarcoma, SKCM = Skin Cutaneous Melanoma, STAD = Stomach adenocarcinoma, TGCT = Testicular Germ Cell Tumours, THCA = Thyroid carcinoma, THYM = Thymoma, UCEC = Uterine Corpus Endometrial Carcinoma, UCS = Uterine Carcinosarcoma, UVM = Uveal Melanoma

**Fig. 3:**
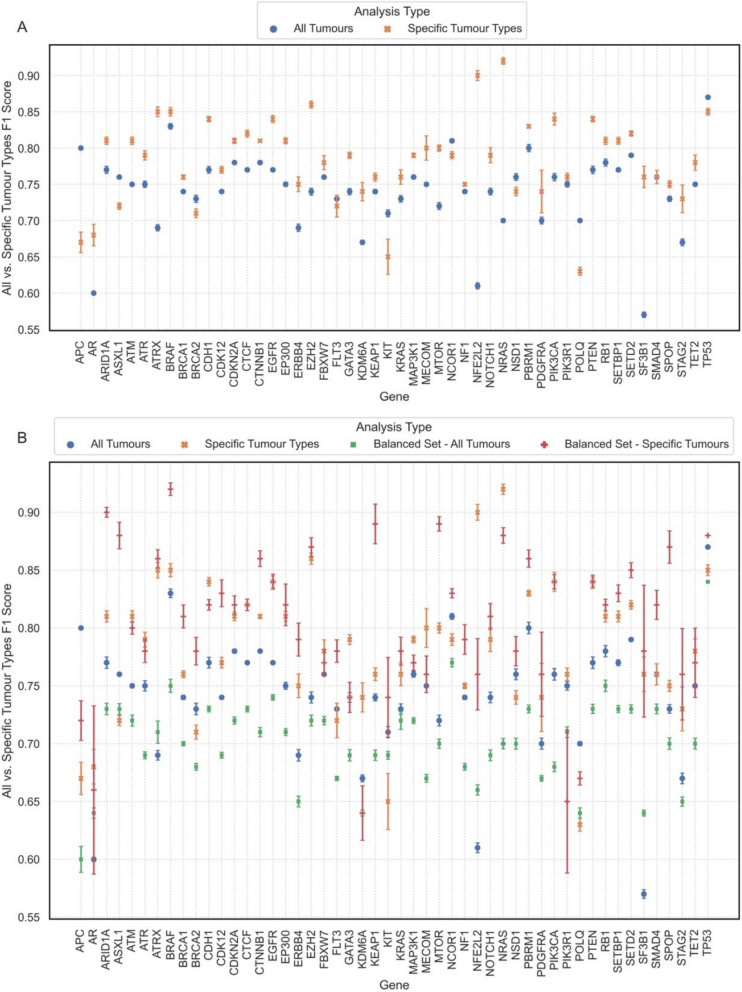
Comparison of F1 scores of classification based on alterations in genes of interest across **A** all versus specific tumour types, **B** four settings: 1. When all samples were used, 2. When samples from selected tumour types were used, 3. When balanced sets of all tumour types were used, and 4. When balanced sets of selected tumour types were used. The dots, crosses, and squares represent the average F1 scores, with whiskers indicating the standard deviation of F1 scores across 30 permutations per setting

Assessing the RF model’s performance across the 33 TCGA cancer cohorts indicated that for certain genes, like *ARID1A*, the model achieved high F1 scores across diverse tumour types (Fig. 2A). For other genes including *BRAF*, strong performance was confined to limited tumour types, particularly thyroid carcinoma and cutaneous melanoma (Fig. 2B). Therefore, it was crucial to ensure that the RF model was not simply learning to classify samples based on their cancer type. F1 scores for individual tumour types were calculated for each gene (Additional file 1: Figure S7), and a *z*-test was employed to find tumour types that could be effectively classified based on alterations in the 50 genes of interest (Additional file 1: Table S1). Evaluating performance on the selected tumour types compared to those from pan-cancer analyses revealed which genes benefitted from tumour-type-specific analysis (Fig. 3A). If the average F1 score improved by more than 5%—as observed for genes like *BRAF*,* ATRX*, and *NRAS*— selected tumour types were utilized for downstream analysis. Otherwise, the transcriptional pattern was interpreted to be more universal, and all tumour types were included to ensure a broader sample set and maximize the likelihood of identifying more generalizable transcriptional patterns.

We next explored the effect of class imbalance within individual tumour types. For *BRAF*, where the model performed well only in thyroid carcinoma and cutaneous melanoma, balancing the underperforming tumour types improved performance considerably in colorectal and stomach adenocarcinomas (Additional file 1: Figure S8). Similarly, balancing for all 50 genes (Additional file 1: Figure S9) identified balanced tumour types where the model performed strongly for each gene (Additional file 1: Table S2). Furthermore, analyses were conducted on balanced sets encompassing all tumour types. Results from pan-cancer analysis, specific tumour types, balanced sets of all tumour types, and balanced sets of specific tumour types were then compared (Fig. 3B). Down-sampling to balance sets of specific tumour types was pursued only when the average F1 score improved by over 5%, as applied to *ARID1A*, *ASXL1*, *BRAF*, *BRCA1*, *CDK12*, *CTNNB1*, *KEAP1*, *MTOR*, *NOTCH1*, *PBRM1*, *PDGFRA*, *SETBP1*, *SETD2*, *SMAD4*, and *SPOP* genes, with a mean F1 improvement of 9.4% (range 6–17%). Balancing across all tumour types did not notably improve performance for any genes.

For genes where the focus of downstream analysis was on multiple tumour types (balanced or unbalanced), transcriptional modifications were investigated both within individual cancer types and across all selected tumour types (Additional file 1: Tables S3-S17). When necessary, analyses were repeated on a subset of cancer types (Additional file 1: Tables S18-S22). Subsequently top-ranked genes from the classification results were extracted, and genes in close chromosomal proximity were excluded (Additional file 1: Table S23). Final lists of highly contributing genes were reviewed (Additional file 1: Tables S24-S56) [28–163], which revealed adjustments necessary specifically for *APC* and *BRAF*. Nearly all the top genes with known associations with *APC* were linked to colorectal cancers, despite training across all tumour types (Additional file 1: Table S57) [28, 164–170], and the model’s performance was lacking across all tumour types, except colorectal cancer (Additional file 1: Figure S10). As this suggests the model was capturing signals related to colorectal cancer rather than the effects of *APC* mutations, the second-best mode of analysis was selected which utilized a balanced set of colorectal cancers for analysis (Fig. 3B). Similarly, almost all genes with established literature associations to *BRAF* were reported in thyroid cancer, despite using balanced sets of thyroid (396 samples) and colorectal cancers (112 samples) in the analysis (Additional file 1: Table S28). To ensure that the larger number of thyroid tumours did not obscure the model’s decision-making process, separate training was conducted on thyroid and colorectal samples. As anticipated, the majority of the top genes from thyroid classification overlapped with the initial list, while those important for classification of colorectal tumours were notably different (Additional file 1: Tables S58 and S59) [50–61, 171–174].

To further validate our classifier approach, we used the Cancer Dependency Map (DepMap), a collection of data from 1673 cancer cell lines from 96 primary diseases. Expression, point mutation, and copy number data for tumour cell lines were obtained from DepMap [175]. Due to notable differences in tumour type representation between TCGA and DepMap data, the three genes with strongest pan-cancer transcriptional patterns were selected, *CDKN2A*,* RB1*, and *TP53*. DepMap expression data only includes protein-coding genes, and therefore RF models were retrained using this subset of genes. The trained RFs were tested on the DepMap data and resulted in F1 scores of 0.57 for both *CDKN2A* and *RB1*, and 0.79 for *TP53*. These results are encouraging given the substantial difference between training and testing tumour types, including the presence of childhood cancers in DepMap not seen during training. Moreover, the variation in F1 scores among the three genes indicates that genes with higher F1 and top Gini scores have more generalizable patterns.

### Examining transcriptional patterns

**Fig. 4:**
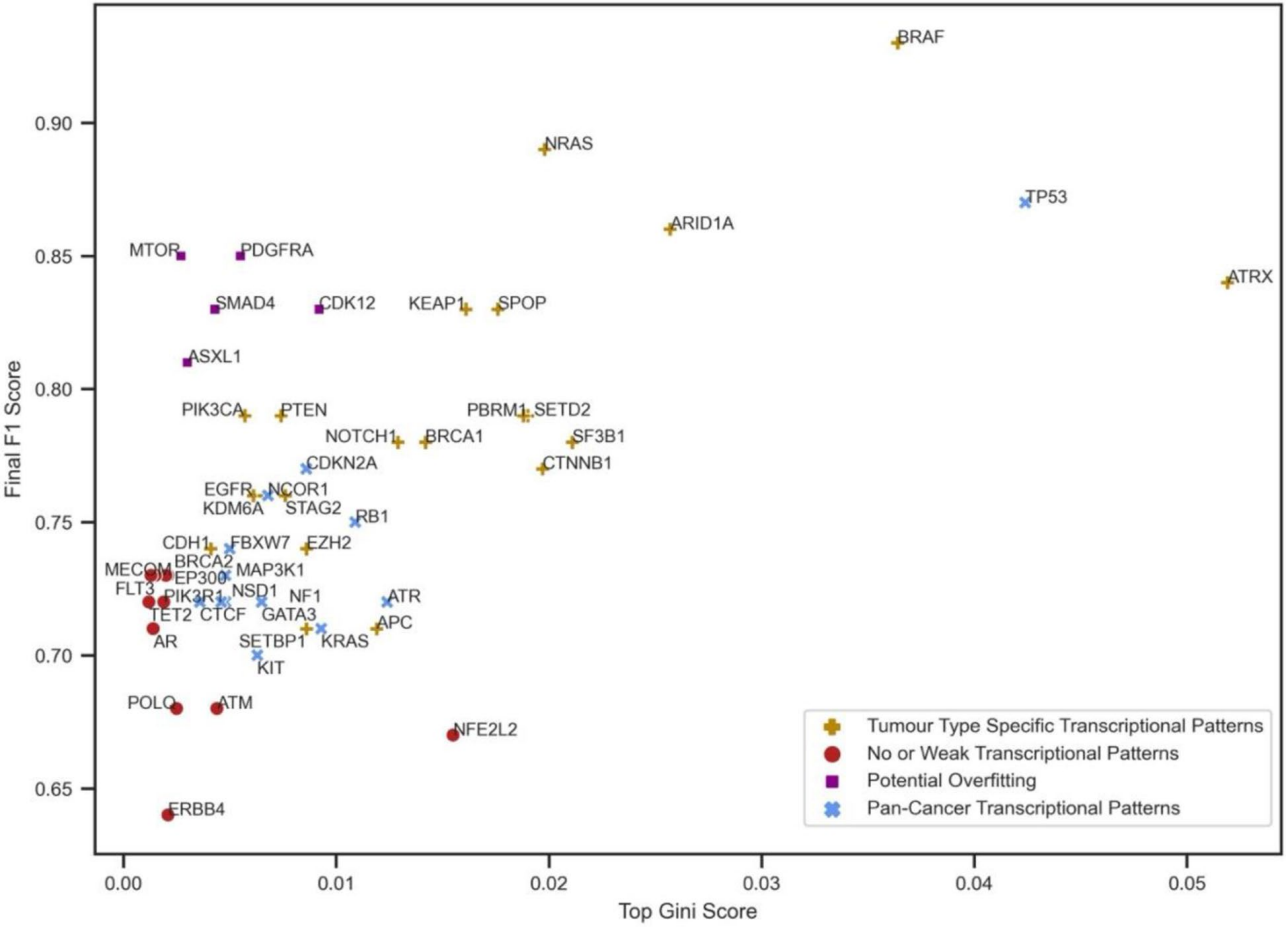
Final F1 score obtained from the optimal mode of analysis against the top Gini score belonging to the feature that contributed the most to classification. Colours and shapes correspond to identified transcriptional pattern categories associated with the genes studied in this work

The optimal analysis settings for each of the 50 genes were used to train RF models, with chromosomally proximal genes removed. Genes contributing significantly to classification were identified based on Gini importance (examples in Additional file 1: Figures S11 and S12). Genes below significance thresholds (Additional file 1: Figures S13-S16; see Methods) were categorized as having no or weak transcriptional patterns associated with their genomic alterations, including *ERBB4*, *POLQ*, and *AR* (Fig. 4). Furthermore, a few genes showed high F1 but low Gini scores, suggesting potential overfitting (Fig. 4). This may be attributed to the limited sample sizes, as analyses for these genes were restricted to balanced sets of only one or two tumour types. These genes, along with those showing no or weak transcriptional patterns, were excluded from further analysis.

**Fig. 5:**
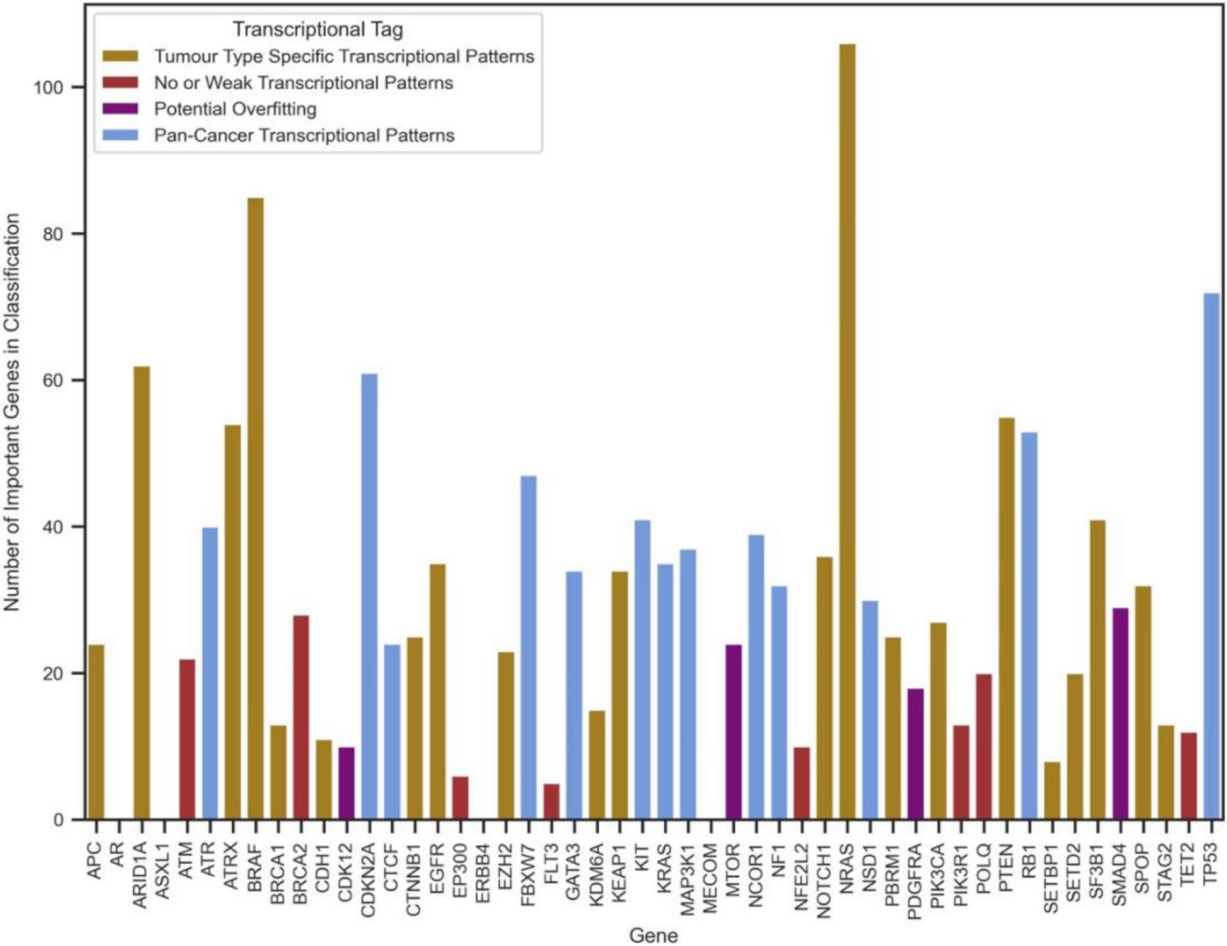
Number of genes found to be highly contributing to the classification task for each gene of interest. Colours represent the associated transcriptional pattern categories

For the remaining genes, the top 15 features with the highest influence on classification were extracted (Additional file 1: Tables S24-S56). Number of highly contributing features to classification of all studied genes can be found in Fig. 5. A literature review identified known associations between these features and the target gene. While clear connections were found for well-studied genes, no known associations were identified for lesser-studied genes, like *KDM6A* and *NSD1*. Our findings suggest that there is value in further investigating these top-ranking genes through wet lab experiments, such as studies in cell lines.

Comparison of overall F1 scores and top Gini scores illustrated stronger signals for genes such as *ATRX*, *BRAF*, and *TP53*. Consequently, it may be that targeting top-contributing genes to classification may offer therapeutic benefits in cancer treatment for patients carrying these driver mutations. It is speculated that the genes near the top right corner of the graph likely exhibit more generalizable transcriptional patterns. To further explore the nature of feature association types to mutational status of *ATRX*,* BRAF*, and *TP53* genes, SHAP analysis was conducted (Additional file 1: Figures S17-S19). This approach further improves biological interpretation of the model by indicating whether a feature drives the prediction toward or away from the wild-type or mutated class. While for *BRAF*, higher expression of the top contributing genes was linked to a mutant prediction, for *ATRX* and *TP53*, elevated expression of top features was associated with wild-type function prediction. This pattern is evident in the SHAP value plots, where higher expression values (indicated in red) are shifted toward the negative class (mutant) for *BRAF* and the positive class (wild-type) for *ATRX* and *TP53.* This is an interesting observation since *BRAF* is an oncogene and its associated genes appear more active in the mutated state, while *ATRX* and *TP53* are tumour suppressors, whose functional networks are downregulated or disrupted in the presence of mutations.

**Fig. 6:**
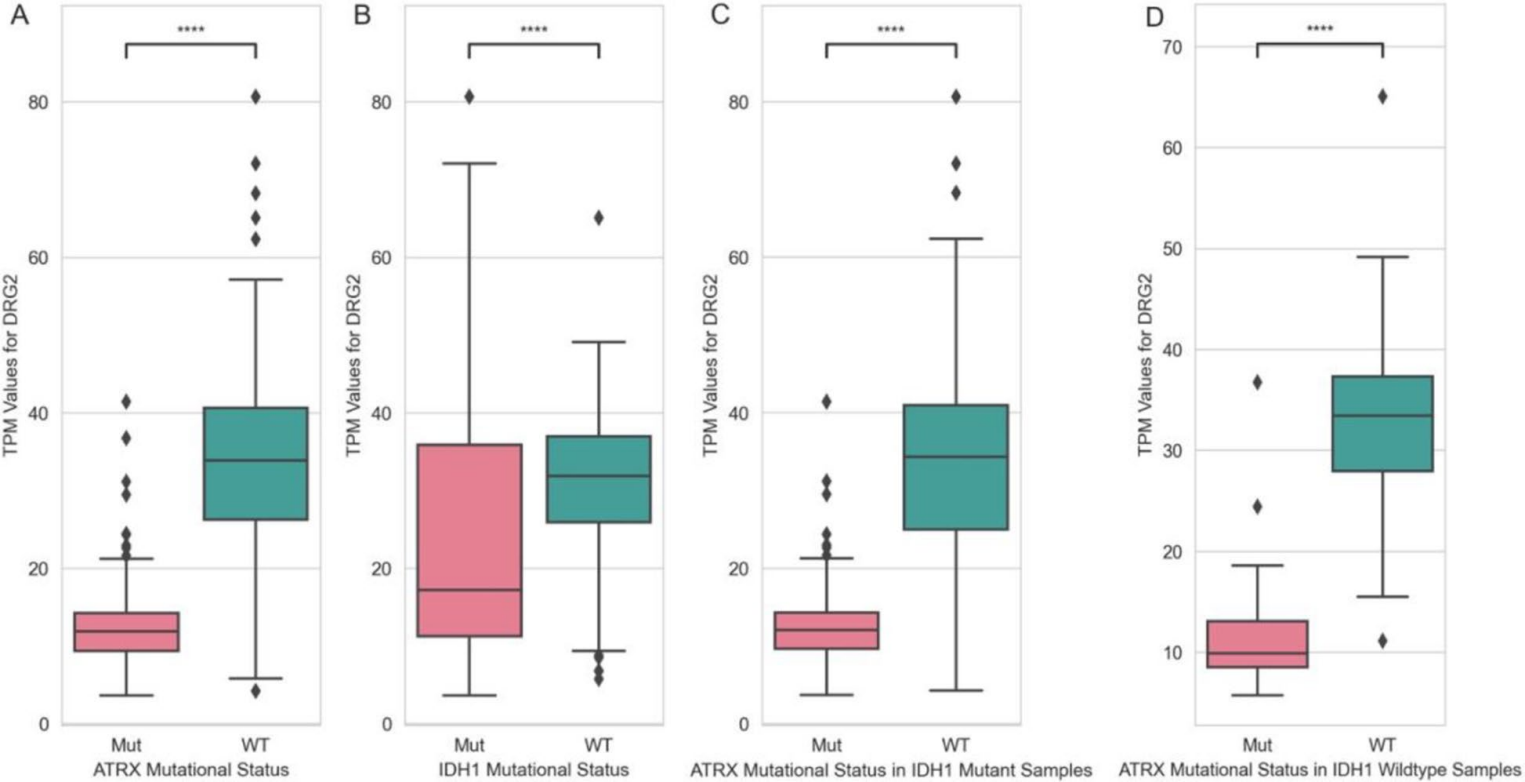
TPM expression values of *DRG2* gene in presence and absence of **A** *ATRX* gene mutations in all LGG samples, **B** *IDH1* gene mutations in all LGG samples, **C** *ATRX* gene mutations in *IDH1* mutant LGG samples, and **D** *ATRX* gene mutations in *IDH1* wild-type samples (*P*-values are obtained in a two-sided Mann–Whitney-Wilcoxon test with Bonferroni correction; *p*-value annotation legend: ns: 5e − 2 < *p* <  = 1; *: 1e − 2 < *p* <  = 5e − 2; **: 1e − 3 < *p* <  = 1e − 2; ***: 1e − 4 < *p* <  = 1e − 3; ****: *p* <  = 1e − 4)

We explored RF model’s predictions for select tumour suppressor genes to assess what aligns with or adds to the existing knowledge about tumours with the associated driver mutations. *ATRX* showed one of the strongest transcriptional signatures in lower-grade gliomas (LGG), with the *DRG2* gene emerging as the top feature in classification. *DRG2* was previously shown to be downregulated in *IDH1*-mutant gliomas, where *ATRX* mutations are common [47]. In our analysis, 179 out of 498 LGG samples (~ 36%) had an *ATRX* mutation, with *DRG2* downregulated in these samples (Fig. 6). While earlier research linked *DRG2* expression to *IDH1* mutations, our findings suggest that *DRG2* expression correlates more strongly with *ATRX* mutational status (*p*-value = 6.79e − 48) than with *IDH1* mutations (*p*-value = 8.88e − 7) (Fig. 6). SHAP analysis further supported this association between *DRG2* downregulation and *ATRX* mutation as lower expression values associated more strongly with the mutant class.

**Fig. 7:**
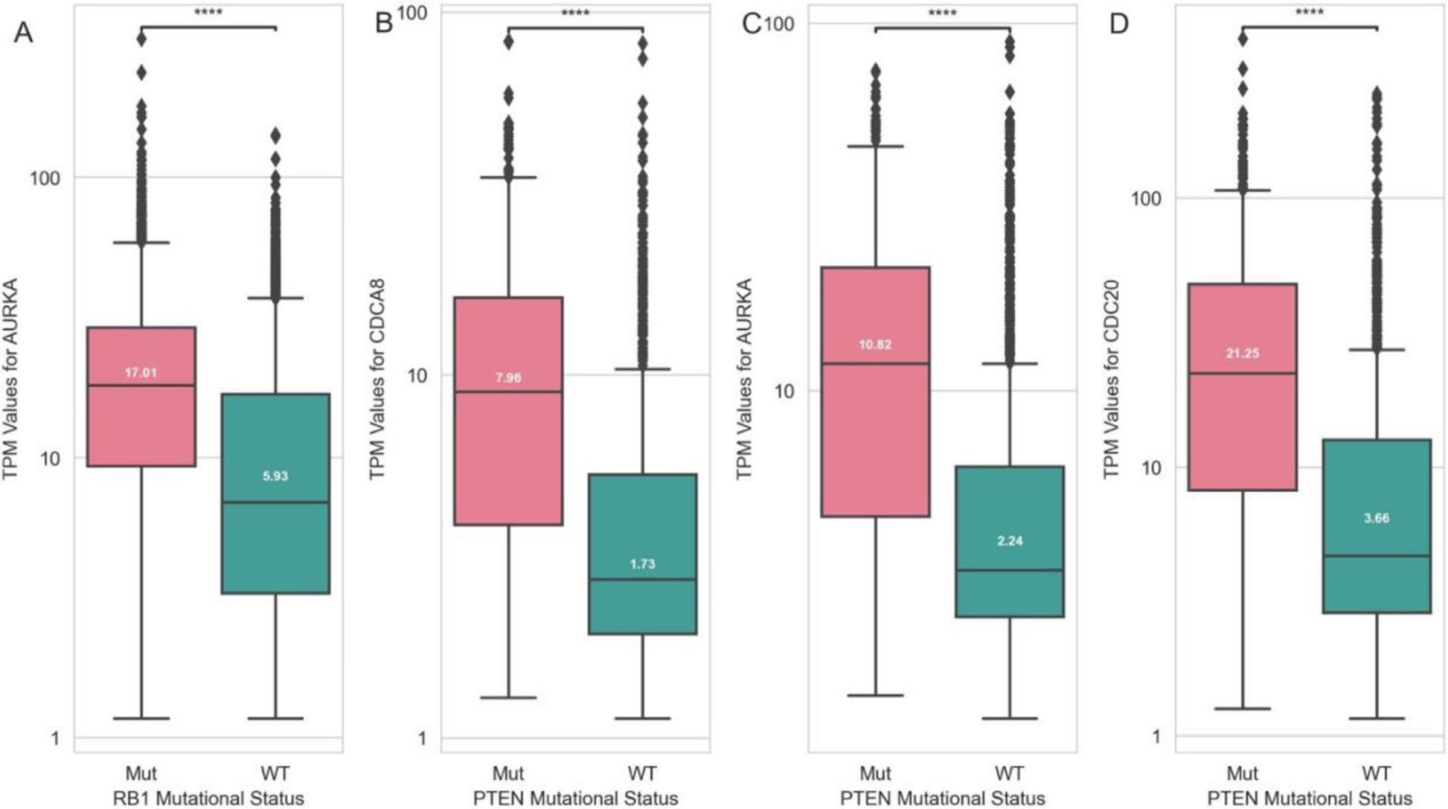
Log 10 of TPM expression values for **A** *AURKA* gene in presence and absence of *RB1* gene mutations across all tumour types, **B** *CDCA8*, **C** *AURKA*, and **D** *CDC20* genes in presence and absence of *PTEN* gene mutations across LGG, PRAD, SARC, and SKCM tumours (numbers inside the boxplots show median values)

*RB1* exhibited a pan-cancer transcriptional signature. Among the 10 genes most associated with *RB1* classification, five were found to have known links to *RB1*. *AURKA*, second in the top-ranked set, was overexpressed in *RB1* mutant cells (*P*-value = 3.40e − 282; Fig. 7A). *AURKA* promotes cancer cell survival, and consistent with our model’s finding, its inhibition has been shown to be effective in *RB1* mutant cells [149, 150]. Similarly, for *PTEN*, 8 out of top 10 genes in classification were found to have known associations with this gene. *AURKA*, ranked second, was upregulated in *PTEN* mutant LGG, PRAD, SARC, and SKCM tumours (*P*-value = 1.04e − 69; Fig. 7C). *CDCA8* and *CDC20*, ranked first and third, were also overexpressed in *PTEN* mutant samples (*P*-values of 3.25e − 72 and 6.39e − 74 respectively; Figs. 7B and 7D). Both genes are involved in cell cycle regulation and negatively affect survival [138, 140, 176], making them attractive targets for cancer therapies. Pathway analysis using top-ranked genes revealed changes in cancer-related pathways such as cell cycle, cell division, and mitosis (Additional file 1: Tables S60-S61) [177, 178].

A fully trained model on samples with impactful mutations and wild-type gene copies can reveal the effects of non-impactful mutations, as demonstrated in our previous work on *TP53* gene where RF classification correctly identified silent mutations with splicing impacts [13]. The final trained RF models were therefore used to predict mutational status of samples harbouring non-impactful mutations (Additional file 1: Table S62). Notably, samples with non-impactful mutations predominantly predicted as mutant consistently involved intron variants. These samples were visualized in IGV (Additional file 1: Figures S20-S23), and it was observed that, in some cases, introns persisted in RNA molecules, indicating splicing errors. For example, *RB1* mutation c.1695 + 24,161 G > T or c.1695 + 29,182 insertion, between exons 17 and 18, led to un-spliced introns. There is also evidence that some variants might disrupt interactions between proteins and RNA strands, potentially affecting post translational changes and protein production. For instance, *EZH2* mutation c.−8 + 9249 A > T, upstream of exon 1, might interfere with protein-RNA interactions, preventing splicing. Further investigation is required to understand how these variants affect the transcriptome and potentially play a role as oncogenic drivers.

Table 1 summarizes the genes studied in this work, with results from the tumour types where classification was most accurate. Details regarding top-ranked genes and affected pathways for *TP53* are omitted, as they were extensively discussed in prior work with very similar scores despite differences in genome versions [13].

**Table 1:** The 50 genes studied in this work, their mode of analysis, final F1 score, and corresponding top Gini score

Gene	Tumour types	Final F1 Score	Top Gini Score
APC	Balanced Set of COADREAD	0.71	0.0119
AR	KIRP	0.71	0.0014
ARID1A	Balanced Set of KICH, KIRP, LGG, PCPG and UVM	0.86	0.0257
ASXL1	Balanced Set of COADREAD	0.81	0.0030
ATM	BRCA, CESC, and PCPG	0.68	0.0044
ATR	All Tumour Types	0.72	0.0124
ATRX	LGG	0.84	0.0519
BRAF	Balanced Set of COADREAD and THCA	0.93	0.0364
BRAF	Balanced Set of COADREAD	0.86	0.0165
BRAF	Balanced Set of THCA	0.93	0.0487
BRCA1	Balanced Set of KICH, KIRP and UCEC	0.78	0.0142
BRCA2	All Tumour Types	0.73	0.0021
CDH1	KIRP, LIHC, PRAD, SARC, THYM, UCEC and UVM	0.74	0.0041
CDK12	Balanced Set of KIRP and UCEC	0.83	0.0092
CDKN2A	All Tumour Types	0.77	0.0086
CTCF	All Tumour Types	0.72	0.0036
CTNNB1	Balanced Set of HNSC, KIRC, PCPG and UVM	0.77	0.0197
EGFR	COADREAD, HNSC, KIRP, LGG and STAD	0.76	0.0061
EP300	BRCA, PCPG, THCA and UCEC	0.73	0.0020
ERBB4	CESC	0.64	0.0021
EZH2	COADREAD, KIRP, LGG and THYM	0.74	0.0086
FBXW7	All Tumour Types	0.74	0.0050
FLT3	All Tumour Types	0.73	0.0015
GATA3	All Tumour Types	0.72	0.0065
KDM6A	KIRP and UCEC	0.76	0.0066
KEAP1	Balanced Set of THCA	0.83	0.0161
KIT	All Tumour Types	0.70	0.0063
KRAS	All Tumour Types	0.71	0.0093
MAP3K1	All Tumour Types	0.73	0.0048
MECOM	All Tumour Types	0.73	0.0013
MTOR	Balanced Set of LGG and PCPG	0.85	0.0027
NCOR1	All Tumour Types	0.76	0.0068
NF1	All Tumour Types	0.72	0.0048
NFE2L2	KICH	0.67	0.0155
NOTCH1	Balanced Set of KIRC	0.78	0.0129
NRAS	LGG and PCPG	0.89	0.0198
NSD1	All Tumour Types	0.72	0.0046
PBRM1	Balanced Set of HNSC, KIRC, MESO, PCPG and UVM	0.79	0.0190
PDGFRA	Balanced Set of KICH	0.85	0.0055
PIK3CA	KIRP, PCPG and UVM	0.79	0.0057
PIK3R1	All Tumour Types	0.72	0.0019
POLQ	All Tumour Types	0.68	0.0025
PTEN	LGG, PRAD, SARC and SKCM	0.79	0.0074
RB1	All Tumour Types	0.75	0.0109
SETBP1	Balanced Set of COADREAD, HNSC and PRAD	0.71	0.0086
SETD2	Balanced Set of HNSC, KIRC, PCPG and UVM	0.79	0.0188
SF3B1	UVM	0.78	0.0211
SMAD4	Balanced Set of COADREAD	0.83	0.0043
SPOP	Balanced Set of KIRP, THCA and THYM	0.83	0.0176
STAG2	KIRP	0.76	0.0076
TET2	All Tumour Types	0.72	0.0012
TP53	All Tumour Types	0.87	0.0424

## Discussion

The integration of transcriptomic data with genomic alterations gives a more comprehensive view of cancer biology and leads to discovery of novel biomarkers [13, 19]. This study examined transcriptomic changes arising from alterations in key cancer driver genes. Our findings revealed that cancer genes fall in distinct groups with respect to their impacts on the transcriptome. Many genes do not confer unique transcriptional patterns following alterations, which may reflect similar impacts on pathways and redundancy in transcriptional signalling. Some genes exhibit tumour-type-specific patterns, including *BRAF*, *ATRX*, and *PTEN*, where transcriptional signatures of mutation are strong but limited to specific tumour types. Conversely, specific cancer driver genes exhibit pan-cancer transcriptional patterns when altered. The most striking example is mutations in *TP53*, as previously described [13], but the findings presented here demonstrate that *CDKN2A*, *RB1*, and *NCOR1* also have notably unique pan-cancer transcriptional impacts. These relate to tumour biology and potentially to association with therapeutic sensitivity, particularly relevant for tumour suppressor mutations which are typically difficult to target [149, 150]. Classification of tumours based on *TP53* mutations revealed that *MYBL2* and *MDM2* play key roles in classification. *MYBL2* is upregulated in *TP53* mutant samples, while *MDM2* is upregulated in *TP53* wild-type samples [13]. This suggests targeting these genes in presence and absence of *TP53* mutations respectively could be an effective strategy for tumour suppression.

*ATRX* showed one of the strongest tumour-type-specific transcriptional patterns in LGG tumours, with *DRG2* gene having the highest Gini score. *DRG2* plays an important role in cell cycle arrest at the G2/M phase during mitosis and affects expression of cell cycle and checkpoint genes [179]. Meanwhile, *ATRX*, a chromatin remodeler, binds cell cycle transition genes, and its loss disrupts the maintenance of the G2/M checkpoint after irradiation [180]. Thus, we hypothesize that *ATRX* mutations affecting the cell cycle may influence *DRG2* expression level. Indeed, our analysis confirmed *DRG2* downregulation in *ATRX* mutant samples, with a stronger correlation to *ATRX* mutations than to *IDH1* mutations, while only correlation with *IDH1* mutations have been discussed previously [47]. These findings suggest that restoring *DRG2* expression in *ATRX* mutant cases could hold therapeutic potential, especially since patients with *IDH1*-mutant tumours were shown to have better prognoses [181, 182].

*RB1* alterations had a pan-cancer transcriptional pattern. *AURKA*, a kinase with roles in mitosis and cancer cell survival [149, 150], contributed most significantly to *RB1* tumour classification. We observed *AURKA* overexpression in *RB1* mutant cells, making it a promising therapeutic target for patients with *RB1* mutations. Indeed, *AURKA* inhibitors have been described as effective against *RB1* mutant cells [149, 150]. This is particularly significant since *RB1*, a tumour suppressor, cannot be targeted directly.

*PTEN*, a frequently mutated tumour suppressor, had transcriptional patterns associated with several tumour types, with *AURKA* emerging as the second most important feature in classification. *AURKA* inhibitors have been shown to be effective in *PTEN* deficient mice [183], highlighting the effectiveness of our model in identifying actionable targets in *PTEN* mutant cases. We found that genes with the highest and third-highest Gini scores, *CDCA8* and *CDC20*, were overexpressed in *PTEN* mutant samples. *CDCA8* competes with *PTEN* for *AKT* binding [138], while *CDC20* physically interacts with *PTEN* in mitotic checkpoint complex [140], both negatively affecting survival [138, 176]. Thus, targeting them as well as *AURKA* could provide therapeutic benefits, particularly in *PTEN* mutant LGG, PRAD, SARC, and SKCM tumours.

Insights gained from the RF model can guide the discovery of new therapeutic strategies. For instance, *AURKA* inhibitors, effective in *RB1* and *PTEN* mutant tumours [149, 150, 183], could be trialed in tumours with mutations in less-studied tumour suppressors, such as *FBXW7* and *NSD1*, for which we found *AURKA* to be a key gene in classification. Additionally, *AURKA* is upregulated when *FBXW7* and *NSD1* are mutated. *FBXW7* has also been shown to negatively regulate *AURKA* [93, 94], further supporting the value in future experiments exploring targeting of *AURKA* in *FBXW7* mutant cell lines and tumours.

For genes with no or weak transcriptional patterns, several mechanisms could explain this outcome. One possibility is that the effect of these gene alterations may be dispersed across multiple genes and pathways, leading to weaker transcriptional signals. Alternatively, these alterations could primarily manifest at the protein level, influencing factors like post-translational modifications, stability, or availability, which may not be directly reflected in the transcriptome [184, 185]. Moreover, some alterations might be clonally diverse, affecting a subset of cells within the tumour [186], making effects harder to detect.

Using the RF classifier on samples with non-impactful mutations was especially valuable where alterations have ambiguous effects on RNA or protein function, helping to uncover insights that may otherwise remain unnoticed. In previous work, we demonstrated that samples with certain mutations labeled as silent actually caused splicing defects [13]. Here, we observed that intron variants might also have pathological effects, impairing the wild-type activity of genes like *NCOR1* and *RB1*. These results underscore the role of non-protein-altering mutations and the importance of detailed investigation of individual mutations.

This analysis is subject to several technical and biological confounding factors, including variations in sample acquisition, data processing, tumour purity, biopsy site, and stromal and immune composition. However, RF models are well-suited to overcome these challenges [187–189], and we observed that they outperformed SVMs, NNs, and XGBoost models. They are also less prone to overfitting compared to other models. However, certain limitations like small sample size and class imbalance can influence the performance, often compounding one another. For example, down-sampling to address class imbalance reduces available data, exacerbating the problem of small sample size. We observed signs of overfitting in some genes, particularly when the analysis was focused on balanced sets of a single or few tumour types. In such cases, although the F1 score was high, the top Gini score remained relatively low, suggesting that the model may have learned patterns specific to the limited dataset rather than generalizable trends. The best strategy to address these issues is to increase the sample size. Large ongoing projects, like the NIH Cancer Moonshot program and the UK Biobank, aim to recruit a large number of participants, to gather health data and sequence genomic material [190, 191]. The large datasets produced by these efforts will help uncover more generalizable patterns in genomics data. Moreover, follow-up studies investigating gene regulatory mechanisms, such as ChIP-seq experiments and protein interaction network analyses, can further enrich the findings from this work and provide validation. Therefore, it is essential to incorporate proteomic, epigenomic, and regulatory data collection alongside genomic variation and expression profiling to support robust conclusions.

This study deepens our understanding of cancer biology and gene interactions, providing a framework for identifying molecular targets in tumours with previously untargetable genes. Here, we showed the importance of combining transcriptomic and genomic data, with potential to facilitate development directions for new targeted therapies. These insights will be instrumental in navigating the ever-evolving landscape of cancer treatment and moving closer to more precise and effective solutions for cancer management.

## Conclusions

In this work, we demonstrated how ML can help unravel complexities of the cancer transcriptome, offering insights that accelerate the development of novel treatments. We identified potential drug targets for eliminating cancer cells by classifying samples based on gene alterations. This analysis led to discovery that *AURKA* inhibitors may be effective in cancers where tumour suppressors like *FBXW7* or *NSD1* are compromised. Additionally, we found a strong correlation between *DRG2* expression levels and *ATRX* gene mutations. These results underscore the potential of computational approaches in identifying new therapeutic strategies tailored to the unique molecular profiles of individual patients.

## Methods

### Data preprocessing

Expression and gene variation data for TCGA were downloaded from online resources [192–195], while data for the POG study was retrieved from Canada’s Michael Smith Genome Sciences Centre servers [7]. Only samples with all data types were included, resulting in 8726 TCGA and 608 POG samples. A list of comparable genes overlapping between TCGA and POG datasets resulted in 56,645 transcribed genes, of which 18,606 (33%) are annotated as protein coding. Principal component analysis (PCA) plots were generated to visualize expression values (Additional file 1: Figures S1-S3). For validation analysis, tumour cell line data was also obtained from DepMap version 24Q4 (https://depmap.org/portal) [175].

The focus of the study was on frequently mutated genes and the ones essential in cancer biology. An overlap of gene lists from related studies yielded 50 shared genes [3, 5, 6]. Tumour samples were grouped by mutational status (mutated vs wildtype) for each gene using somatic and germline mutation data. The samples in the mutated group were further divided into “impactful” and “non-impactful” categories based on expected consequences of mutations [13, 196]. Samples containing “impactful” mutations or wildtype gene copies were used to create feature matrices. For analyses including CNA and SV data, samples with copy number changes or structural variations were reclassified as “impactful”.

### Random Forests performance

Performance of RF, SVM, and NN models were compared on classification of *TP53* mutational status (Additional file 1: Figure S4). Main hyperparameters were fine-tuned using 90% of samples and validated on the remaining 10%. Samples were categorized as either wild-type or mutant based on SNV/INDEL data. The performance of the RF model was evaluated using 5-fold cross-validation (CV) across TCGA and POG datasets for all the genes of interest, and F1 scores were computed (Fig. 1A). To evaluate the impact of additional gene alterations, samples with CNAs or SVs were re-classified as mutant, and the RF model’s performance was evaluated (Additional file 1: Figure S5). Since SVs had minimal impact on F1 scores, the focus was narrowed to SNVs/INDELs and CNAs (Fig. 1B). Samples with CNAs were only labeled as mutant if their inclusion resulted in a substantial improvement in F1 score. To validate this improvement, test F1 scores on 10% of samples were compared to the 5-fold CV F1 scores from the 90% training set (Additional file 1: Figure S6). The RF model was then trained on all TCGA and POG samples and the final F1 score were recorded (Fig. 1C).

### Tumour type-specific analysis and class imbalance at the tumour type level

F1 scores were computed across 33 TCGA tumour types (Fig. 2). To account for F1 score variability in some genes, a *z*-test with alpha of 0.1 was conducted, establishing a significance threshold of 0.728 (Additional file 1: Figure S7). For each gene of interest, tumour types with F1 scores above this threshold were selected for downstream analysis. If no tumour type met the threshold, the one with the highest F1 score was chosen (Additional file 1: Table S1). Model’s performance was compared between pan-cancer and tumour type-specific analyses (Fig. 3A). Tumour-specific analyses were pursued only if the average F1 score improved by more than 5%.

The impact of class imbalance at the tumour type level was then examined. Balancing tumour types for *BRAF* mutations improved F1 scores (Additional file 1: Figure S8), and thus, performance was tested on balanced sets of tumour types for all genes. A *z*-test on F1 scores with alpha of 0.1 resulted in a significance threshold of 0.785 (Additional file 1: Figure S9). Tumour types with F1 scores above this threshold or the one with the highest balanced F1 score (Additional file 1: Table S2) were used to perform 30 permutations of 5-fold CV. Additionally, balanced sets from all tumour types were analysed in the same way, and average F1 scores were compared across all settings (Fig. 3B). Subsequently, the analysis focused on balanced sets of specific tumour types only when the average F1 score showed an improvement of more than 5%. For multi-tumour-type analyses, model performance was investigated both within individual cancer types and across the whole set (Additional file 1: Tables S3-S17). If performance dropped for one of the tumour types, that tumour type was excluded, and performance was re-evaluated (Additional file 1: Tables S18-S22).

Subsequently, the lists of top-ranked genes in classification were examined. In some instances, the majority of top genes were located at nearby chromosomal regions to the gene under investigation, likely due to inclusion of CNAs in the analysis. To ensure that the observed transcriptional modifications were associated with alterations in the function of the genes of interest, nearby genes were iteratively removed until no cluster of physically adjacent genes appeared among the top-ranked genes (Additional file 1: Table S23) [197, 198]. The top genes in classification were then studied for their associations with the genes of interest, based on retrieved literature (Additional file 1: Tables S24-S56). In specific cases, like *APC* and *BRAF*, evaluating top genes in classification led to a revision of the analysis approach (Additional file 1: Figure S10 and Tables S57-S59).

### Examining transcriptional patterns

After training, thresholds were established for number of key genes in classification using a permutation-based method (Additional file 1: Figures S11 and S12) [13]. For certain genes of interest, no genes were found to have a significant impact, typically with low F1 or top Gini scores. These genes were considered to have no or weak transcriptional patterns (Additional file 1: Figures S13 and S14). To define a threshold, a *z*-test with alpha 0.1 was applied on 5-fold CV F1 scores and a lower percentile with alpha 0.25 on top Gini scores (Additional file 1: Figures S15 and S16). A lower threshold of 0.686 for F1 and a lower critical value of 0.0021 for Gini score were obtained. Genes below these thresholds were categorized as having no or weak transcriptional patterns (Fig. 4). Moreover, a few genes were identified with high F1 scores but low top Gini scores. These genes were flagged for potential overfitting, especially due to small training sets. Consequently, these genes, along with those categorized as having no or weak transcriptional patterns, were excluded from further analysis.

For the remaining genes, associations between the top contributing genes and genes under investigation were gathered from the literature (Additional file 1: Tables S24-S56). The number of important genes in classification is shown in Fig. 5. These genes were further used in Gene Set Enrichment Analysis using the Database for Annotation, Visualization, and Integration Discovery [177, 178]. The top five enriched pathways with significant *p*-values are in Additional file 1: Table S60. SHAP importance was also found using the Python SHAP package for the top 15 features of the three genes with highest top Gini scores (Additional file 1: Figures S17-S19) [199]. The mutational status of samples with non-impactful mutations was also predicted. When the majority of samples were classified as mutant, these were further examined using Integrative Genomic Viewer (IGV) [200].

## Supplementary Information

Additional file 1: This file contains a detailed description of the methodologies used for data analysis, along with supplementary tables S1-S62 and supplementary figures S1-S23. TableS1 – Tumour types selected for downstream analysis based on all samples. TableS2 – Tumour types selected for downstream analysis based on balanced sets of samples. Tables S3 to S17 – Number of mutant and wild-type samples and F1 scores based on alterations in *ARID1A*, *BRAF*, *BRCA1*, *CDH1*, *CTNNB1*, *EGFR*, *EZH2*, *KDM6A*, *NRAS*, *PBRM1*, *PIK3CA*, *PTEN*, *SETBP1*, *SETD2*, and *SPOP*. Tables S18 to S22 – Number of mutant and wild-type samples and F1 scores after excluding specific tumour types for *ARID1A*, *EGFR*, *EZH2*, *PBRM1*, and *SPOP*. Table S23 – Chromosomal regions excluded from analysis. Tables S24 to S56 – Top genes in classification of samples based on alterations in *APC*, *ARID1A*, *ATR*, *ATRX*, *BRAF*, *BRCA1*, *CDH1*, *CDKN2A*, *CTCF*, *CTNNB1*, *EGFR*, *EZH2*, *FBXW7*, *GATA3*, *KDM6A*, *KEAP1*, *KIT*, *KRAS*, *MAP3K1*, *NCOR1*, *NF1*, *NOTCH1*, *NRAS*, *NSD1*, *PBRM1*, *PIK3CA*, *PTEN*, *RB1*, *SETBP1*, *SETD2*, *SF3B1*, *SPOP*, and *STAG2*. Tables S57 to S59 – Top genes in classification of samples based on alterations in *APC* and *BRAF* under different settings. Table S60 – Top pathways affected by gene alterations. Table S61 – Top pathways affected by BRAF gene alterations in thyroid and colorectal cancers. Table S62 – Non-impactful mutations likely playing a role in pathogenesis. Figures S1 to S3 – PCA plots of POG, TCGA, and all samples. Figure S4 – Performance comparison across different models. Figure S5 – F1 scores based on different sets of gene alterations. Figure S6 – F1 score comparison between 5-fold CV and test set. Figure S7 – F1 scores distribution across all genes and tumour types. Figure S8 – Tumour-type-level F1 scores for *BRAF*. Figure S9 – F1 scores distribution based on balanced sets. Figure S10 – Tumour-type-level F1 scores for APC. Figures S11 to S14 – Gini scores based on true and randomly shuffled labels for *KRAS*, *PTEN*, *AR*, and *ERBB4*. Figure S15 – F1 score distribution across all genes. Figure S16 – Gini score distribution across all genes. Figures S17 to S19 – Top genes SHAP values for *ATRX*, *BRAF* and *TP53*. Figures S20 to S23 – Samples with intron variants predicted as mutant for *EGFR*, *EZH2*, *NCOR1*, and *RB1*.

## Data Availability

• Expression matrices containing TPM (transcript per million) values and CNA data were obtained from the University of California Santa Cruz repository (192). SNV/INDEL data files were downloaded from GDC data portal (193), and germline mutation data file was downloaded from genomic data commons website (194). Structural variation files for TCGA study were downloaded from cBioPortal (195). • Genomic and transcriptomic sequence datasets for the POG program are available at the European Genome-phenome Archive (EGA, https://ega-archive.org/) as part of the study EGAS00001001159. • The code used in this project can be found in this GitHub Repository: https://github.com/FaezeK/Cancer_Gene_Mutational_Status_Classifier.

## Abbreviations

ACC: Adrenocortical carcinoma
BC: British Columbia
BLCA: Bladder urothelial carcinoma
BRCA: Breast invasive carcinoma
CESC: Cervical squamous cell carcinoma and endocervical adenocarcinoma
CHOL: Cholangiocarcinoma
CNA: Copy number alteration
COAD: Colon adenocarcinoma
CV: Cross validation
DLBC: Lymphoid neoplasm diffuse large B-cell lymphoma
ESCA: Esophageal carcinoma
GBM: Glioblastoma multiforme
HNSC: Head and neck squamous cell carcinoma
IGV: Integrative Genomics Viewer
INDEL: Insertion/Deletion
KICH: Kidney chromophobe
KIRC: Kidney renal clear cell carcinoma
KIRP: Kidney renal papillary cell carcinoma
LAML: Acute myeloid leukaemia
LGG: Brain lower-grade glioma
LIHC: Liver hepatocellular carcinoma
LUAD: Lung adenocarcinoma
LUSC: Lung squamous cell carcinoma
MESO: Mesothelioma
ML: Machine learning
NN: Neural Network
OV: Ovarian serous cystadenocarcinoma
PAAD: Pancreatic adenocarcinoma
PCA: Principal component analysis
PCPG: Pheochromocytoma and Paraganglioma
POG: Personalized Oncogenomics
PRAD: Prostate adenocarcinoma
READ: Rectum adenocarcinoma
RF: Random Forest
SARC: Sarcoma
SKCM: Skin cutaneous melanoma
SNV: Single-nucleotide variation
STAD: Stomach adenocarcinoma
SV: Structural variant
SVM: Support Vector Machine
TCGA: The Cancer Genome Atlas
TGCT: Testicular germ cell tumours
THCA: Thyroid carcinoma
THYM: Thymoma
UCEC: Uterine corpus endometrial carcinoma
UCS: Uterine carcinosarcoma
UVM: Uveal melanoma
