# Supplementary material for: Transcriptional patterns of cancer-related genes in primary and metastatic tumours revealed by machine learning
Source: BMC Biol. 2025 Aug 7;23:246. doi: 10.1186/s12915-025-02339-z (PMC12329921; doi:10.1186/s12915-025-02339-z)
Supplement: Supplementary file 1 — Additional file 1: This file contains a detailed description of the methodologies used for data analysis, along with supplementary tables S1-S62 and supplementary figures S1-S23. TableS1 – Tumour types selected for downstream analysis based on all samples. TableS2 – Tumour types selected for downstream analysis based on balanced sets of samples. Tables S3 to S17 – Number of mutant and wild-type samples and F1 scores based on alterations in ARID1A, BRAF, BRCA1, CDH1, CTNNB1, EGFR, EZH2, KDM6A, NRAS, PBRM1, PIK3CA, PTEN, SETBP1, SETD2, and SPOP. Tables S18 to S22 – Number of mutant and wild-type samples and F1 scores after excluding specific tumour types for ARID1A, EGFR, EZH2, PBRM1, and SPOP. Table S23 – Chromosomal regions excluded from analysis. Tables S24 to S56 – Top genes in classification of samples based on alterations in APC, ARID1A, ATR, ATRX, BRAF, BRCA1, CDH1, CDKN2A, CTCF, CTNNB1, EGFR, EZH2, FBXW7, GATA3, KDM6A, KEAP1, KIT, KRAS, MAP3K1, NCOR1, NF1, NOTCH1, NRAS, NSD1, PBRM1, PIK3CA, PTEN, RB1, SETBP1, SETD2, SF3B1, SPOP, and STAG2. Tables S57 to S59 – Top genes in classification of samples based on alterations in APC and BRAF under different settings. Table S60 – Top pathways affected by gene alterations. Table S61 – Top pathways affected by BRAF gene alterations in thyroid and colorectal cancers. Table S62 – Non-impactful mutations likely playing a role in pathogenesis. Figures S1 to S3 – PCA plots of POG, TCGA, and all samples. Figure S4 – Performance comparison across different models. Figure S5 – F1 scores based on different sets of gene alterations. Figure S6 – F1 score comparison between 5-fold CV and test set. Figure S7 – F1 scores distribution across all genes and tumour types. Figure S8 – Tumour-type-level F1 scores for BRAF. Figure S9 – F1 scores distribution based on balanced sets. Figure S10 – Tumour-type-level F1 scores for APC. Figures S11 to S14 – Gini scores based on true and randomly shuffled labels for KRAS, PTEN, AR, and ERBB4. Figur [file 12915_2025_2339_MOESM1_ESM.pdf]

Supplementary File

Extended Methods

Data Preprocessing

Expression matrices containing TPM (transcript per million) values and CNA data were obtained from the University of California Santa Cruz repository. SNV/INDEL data files were downloaded from GDC data portal, and germline mutation data file was downloaded from genomic data commons website. Structural variation files for TCGA study were downloaded from cBioPortal. As for the POG study, processed expression and gene variation data from 608 patients with metastatic disease who were recruited before any chemotherapy or after one line of chemotherapy were obtained from the Canada's Michael Smith Genome Sciences Centre servers. TCGA expression, somatic SNV/INDEL and CNA data files were used to find samples with all data types which provided 8726 samples. TCGA and POG expression matrices were consolidated based on a list of comparable genes that overlap between both datasets that resulted in a total of 56,645 transcribed genes, of which 18,606 (33%) are annotated as protein coding. The Principal Component Analysis (PCA) plots of log2 (TPM+0.001) values showed that, despite tumour or disease (metastatic or primary tumour) type, expression profiles clustered together (Figures S1-S3).

To increase the likelihood of benefiting a larger number of patients while keeping a feasible list of genes, frequently mutated genes and the ones essential in cancer biology were prioritized. Using a minimum threshold of 2% mutation rate across all cancers, 135 frequently mutated genes were obtained from the Mendiratta et al study. Overlap with Kandoth et al. paper's 127 significantly mutated genes and 299 cancer driver genes found by Bailey et al. yielded 50 shared genes for downstream analysis of transcriptome modifications. Tumour samples were grouped by mutational status (mutated vs wildtype) for each gene using somatic and germline mutation data. The samples in the mutated group were further divided into "impactful" and "non-impactful" categories based on expected consequences of mutations. The samples containing mutations deemed "Low" or "Modifier" impact by Ensembl (version 107), were placed in the "non-impactful" group, as these either result in no protein modification or have uncertain effects. The remaining samples containing mutations were categorized as "impactful" and were used to create feature matrices along with samples containing wildtype gene copies. Samples with "non-impactful" mutations were excluded to increase the likelihood of only pathogenic driver alterations being used for learning. For analyses including CNA and SV data, samples with copy number changes or structural variations were reclassified as "impactful".

Random Forests Initial Performance

Prior to training the RF model, the main hyperparameters were fine-tuned using 90% of the available samples, following the approach detailed in our prior study. The obtained values were subsequently validated using the remaining 10% of the samples to ensure the model's generalizability. Samples initially were categorized as either wild-type or mutant based solely on SNVs/INDELs data. The performance of the RF model was evaluated using 5-fold cross-validation (CV) across both TCGA and POG datasets for all the genes of interest. F1 scores were computed to gauge the model's effectiveness, and the ratios of samples with "impactful" mutations to those with wild-type gene copies were also calculated. These ratios provided insight into how the imbalance between the two classes might influence the performance (Figure 1A).

Addition of More Data Types

To evaluate the impact of additional gene alterations, CNAs and SVs were incorporated into the analyses. Samples with different combinations of alterations were categorized as mutants, and the RF model's performance was evaluated across 30 permutations for each setting. Average F1 scores and standard deviations were calculated and compared (Figure S5). Since SVs had minimal impact on F1 scores, the focus was narrowed to SNVs/INDELs and CNAs (Figure 1B). Samples with CNAs were only labeled as mutant if their inclusion resulted in a substantial improvement in F1 score.

To validate the analysis settings (using SNVs/INDELs data alone or combined with CNAs), 10% of samples were randomly set aside, while maintaining the mutant-to-wild-type ratio. The model was trained on the remaining samples and tested on the reserved 10%. The test F1 scores were compared to the previously obtained 5-fold CV F1 scores (Figure S6). Once settings were confirmed, the RF model was trained on all TCGA and POG samples. The final F1 score and the ratio of mutant to wild-type samples were recorded (Figure 1C).

Tumour Type Specific Analysis and Class Imbalance at the Tumour Type Level

To determine whether the model's strong performance is specific to certain cancer types or consistent across a pan-cancer setting, F1 scores were plotted against the ratio of minor (the set of either mutant or wild-type samples, whichever is smaller) to major group sizes across 33 TCGA tumour types (Figure 2). F1 scores for some genes varied widely across tumour types, so a statistical test was used to establish significance. Specifically, for each gene and tumour type

with at least 10 mutated samples, the RF model was evaluated using 5-fold CV. F1 scores were plotted (Figure S7), and since the distribution followed a normal curve, a z-test with alpha of 0.1 was performed. A significance threshold of 0.728 was obtained for F1 scores. For each gene of interest, tumour types with F1 scores above this threshold were selected for downstream analysis, or the tumour type with the highest F1 score was chosen if none met the threshold (Table S1).

To compare the model's performance between pan-cancer and tumour type specific analyses, selected tumour types were used in 30 permutations of 5-fold CV. Average and standard deviation of F1 scores were calculated and compared to the pan-cancer results (Figure 3A). Tumour-specific analyses were pursued only if the average F1 score improved by more than 5%, accounting for reduced sample size.

The impact of class imbalance at the tumour type level was then examined. Tumour types with poor classification performance for *BRAF* mutations were balanced and the model's performance was re-evaluated (Figure S8). This improved F1 scores for some tumour types, prompting further analysis. Specifically, for each gene and tumour type with at least 10 mutated samples, the larger group (mutated or wild-type) was down-sampled to create balanced sets. 5-fold CV was performed, and F1 scores were obtained. A distribution graph of the F1 scores was generated (Figure S9) and a z-test with alpha of 0.1 resulted in a significance threshold of 0.785. Tumour types with F1 scores above this threshold were selected for downstream analysis. If the F1 score of no tumour type exceeded this score, the one with the highest balanced F1 score was chosen (Table S2).

30 permutations of 5-fold CV were performed on the selected tumour types, and the average F1 scores and standard deviations were calculated. Additionally, balanced sets from all tumour types were analyzed in the same way, and average F1 scores were compared across all settings (Figure 3B). Subsequently, the analysis focused on balanced sets of specific tumour types only when the average F1 score showed an improvement of more than 5%, as down-sampling reduces training samples. For genes where the most significant F1 scores were achieved in settings with multiple tumour types (balanced or unbalanced), model performance was further investigated both within individual cancer types and across the combined set (Tables S3-S17). If performance dropped for one of the tumour types in the combined set, that tumour type was excluded, and performance was re-evaluated (Tables S18-S22).

Examining the list of top-ranked genes in classification revealed that in some instances, all or majority of the genes were located at nearby chromosomal regions to the gene under investigation, likely due to the inclusion of CNAs in the analysis. This aligns with biological expectations, because CNAs typically affect larger chromosomal regions compared to SNVs/INDELs and genes located in proximity are likely impacted by the same copy number events, resulting in correlated expression changes. To mitigate this and ensure that the observed transcriptional modifications were associated with alterations in the function of the genes of

interest, nearby cytobands containing genes that highly contributed to classification were flagged. The cytobands coordinates were found using the NCBI ideogram file, and genes within the cytobands were obtained via the BioMart data mining tool. Genes from these regions were iteratively removed until no cluster of physically adjacent genes appeared among the top-ranked genes. The final excluded chromosomal coordinates are in table S23. Once removing nearby genes, the top contributing genes to classification were examined for their associations with the genes of interest, based on retrieved literature (Tables S24-S56). In specific cases, like *APC* and *BRAF*, evaluating F1 scores and top genes in classification led to a revision of the analysis approach (Figure S10 and Tables S57-S59).

Examining Transcriptional Patterns

After determining the optimal analysis setting and training the model for each gene of interest, thresholds for selecting key genes in classification were established using a permutation-based method, described in our previous work. Gini importance scores were plotted to gene ranks, and thresholds for identifying the top genes were set accordingly (examples are provided in Figures S11 and S12).

Some genes, when trained with the top 1000 genes (features) and randomly shuffled labels, showed higher feature importance scores than when using the true mutational labels. These genes also tended to exhibit lower F1 or top Gini scores compared to other genes. Thus, these genes were considered to have no or weak transcriptional patterns (Figures S13 and S14). To formally define a threshold for no or weak transcriptional signals, 5-fold CV F1 scores, along with the Gini score of the most influential feature in classification, were obtained and their distributions were plotted (Figures S15 and S16). Since the distribution of F1 scores followed a normal curve, a z-test with an alpha of 0.1 was performed, yielding a significance lower threshold of 0.686. The distribution of top Gini scores was right-skewed, so the lower percentile was calculated with an alpha of 0.25, giving a lower critical value of 0.0021. Genes with F1 scores below 0.686 or top Gini scores below 0.0021 were categorized as having no or weak transcriptional patterns. Moreover, a few genes were identified with high F1 scores but low top Gini scores. These genes were flagged for potential overfitting, especially since for all of them, a balanced set of one or two tumour types were used during classification. Low Gini scores also indicate that none of the features contributed meaningfully to the classification. Consequently, these genes, along with those categorized as having no or weak transcriptional patterns, were excluded from further analysis.

For the remaining genes, associations between the top 15 genes and the genes of interest were gathered from literature (Tables S24-S56). If fewer than 15 genes passed the threshold, associations for all contributing genes were investigated. The number of important genes in the

classification is shown in figure 5. These genes were further used in Gene Set Enrichment Analysis (GSEA) using the Database for Annotation, Visualization, and Integration Discovery (DAVID). A threshold of 0.05 was applied for p-values, adjusted for multiple testing using the Benjamini-Hochberg method. The top five enriched pathways for genes of interest are in table S60. If fewer than five pathways met the threshold, all were reported. SHAP importance was also found using the Python SHAP package for the top 15 features of the three genes with highest top Gini scores (Figures S17-S19).

Next, the mutational status of samples with non-impactful mutations was predicted using the fully trained RF model. When the majority of samples were classified as mutant, these were further examined using the Integrative Genomic Viewer (IGV), as it was expected to have no change in the produced proteins and a similar behavior to the wildtype category.

Supplementary Tables

Table S1. Tumour types selected for downstream analysis on tumour-type specific transcriptional modifications. COAD and READ tumour types were combined. TCGA tumour types: ACC=Adrenocortical carcinoma, BLCA=Bladder Urothelial Carcinoma, BRCA=Breast invasive carcinoma, CESC=Cervical squamous cell carcinoma and endocervical adenocarcinoma, CHOL=Cholangiocarcinoma, COAD=Colon adenocarcinoma, DLBC=Lymphoid Neoplasm Diffuse Large B-cell Lymphoma, ESCA=Esophageal carcinoma, GBM=Glioblastoma multiforme, HNSC=Head and Neck squamous cell carcinoma, KICH=Kidney Chromophobe, KIRC=Kidney renal clear cell carcinoma, KIRP=Kidney renal papillary cell carcinoma, LAML=Acute Myeloid Leukemia, LGG=Brain Lower Grade Glioma, LIHC=Liver hepatocellular carcinoma, LUAD=Lung adenocarcinoma, LUSC=Lung squamous cell carcinoma, MESO=Mesothelioma, OV=Ovarian serous cystadenocarcinoma, PAAD=Pancreatic adenocarcinoma, PCPG=Pheochromocytoma and Paraganglioma, PRAD=Prostate adenocarcinoma, READ=Rectum adenocarcinoma, SARC=Sarcoma, SKCM=Skin Cutaneous Melanoma, STAD=Stomach adenocarcinoma, TGCT=Testicular Germ Cell Tumors, THCA=Thyroid carcinoma, THYM=Thymoma, UCEC=Uterine Corpus Endometrial Carcinoma, UCS=Uterine Carcinosarcoma, UVM=Uveal Melanoma.

Gene	Tumour Types
APC	COADREAD
AR	KIRP
ARID1A	BRCA, LGG, LIHC, PCPG, UVM
ASXL1	BRCA, CESC
ATM	BRCA, CESC, PCPG
ATR	HNSC, KIRP, PCPG
ATRX	LGG
BRAF	COADREAD, SKCM, THCA

BRCA1	BRCA, KICH, KIRP, UCEC
BRCA2	BRCA
CDH1	KIRP, LIHC, PRAD, SARC, THYM, UCEC, UVM
CDK12	BRCA, KICH, KIRP, SARC, UCEC
CDKN2A	HNSC, KIRC, LGG, MESO, PAAD, UCEC
CTCF	KIRP, LIHC, PRAD, SARC, THYM, UVM
CTNNB1	BRCA, CESC, HNSC, KIRC, LIHC, PCPG, UVM
EGFR	COADREAD, HNSC, KIRP, LGG, STAD, THCA
EP300	BRCA, PCPG, THCA, UCEC
ERBB4	CESC, PAAD
EZH2	COADREAD, KIRP, LGG, THCA, THYM
FBXW7	LIHC, MESO, UCEC
FLT3	UCEC
GATA3	LGG, SARC
KDM6A	KIRP, UCEC
KEAP1	GBM, LUAD, STAD, UCEC
KIT	ACC, KICH
KRAS	COADREAD, PAAD
MAP3K1	BLCA, HNSC, PRAD, STAD
MECOM	UCEC, UVM
MTOR	BRCA, LGG, PCPG
NCOR1	BRCA, COADREAD, KICH, KIRP, LIHC, PAAD, PCPG, UCEC
NF1	BRCA, KICH, KIRP, PCPG, SARC, UCEC
NFE2L2	KICH, THCA
NOTCH1	KIRC
NRAS	LGG, PCPG
NSD1	BLCA, HNSC, KIRC
PBRM1	BRCA, CESC, HNSC, KIRC, KIRP, PCPG, UVM
PDGFRA	KICH
PIK3CA	KIRP, PCPG, UVM
PIK3R1	BLCA, HNSC, STAD
POLQ	BRCA
PTEN	LGG, PRAD, SARC, SKCM
RB1	BRCA, CESC, COADREAD, GBM, LGG, LIHC, PRAD, SARC
SETBP1	HNSC, PRAD
SETD2	BRCA, CESC, HNSC, KIRC, KIRP, PCPG, UVM
SF3B1	UVM
SMAD4	STAD
SPOP	BRCA, KICH, KIRP
STAG2	KIRP
TET2	LIHC, MESO
TP53	BLCA, BRCA, COADREAD, HNSC, LGG, LIHC, LUAD, STAD, UCEC

Table S2. Tumour types selected for downstream analysis on examining transcriptional modifications using balanced sets of specific tumour-type. COAD and READ tumour types were combined.

Gene	Tumour Types

APC	COADREAD
AR	THCA
ARID1A	KICH, KIRP, LGG, PCPG, THYM, UVM
ASXL1	COADREAD
ATM	BRCA, CESC, PCPG, TGCT
ATR	CESC, KIRP, PCPG
ATRX	LGG, UCEC
BRAF	COADREAD, THCA
BRCA1	KICH, KIRP, UCEC
BRCA2	COADREAD
CDH1	BRCA, KIRP, LIHC, OV, PRAD, UVM
CDK12	KIRP, UCEC
CDKN2A	KIRC, KIRP, PAAD, THCA, UCEC
CTCF	BRCA, KIRP, LIHC, PRAD, THYM
CTNNB1	HNSC, KIRC, PCPG, UVM
EGFR	COADREAD, KIRP, LGG
EP300	THCA, UVM
ERBB4	CESC
EZH2	KIRP, LGG, THCA, THYM
FBXW7	LIHC, MESO
FLT3	COADREAD
GATA3	KIRP, LGG
KDM6A	KIRC
KEAP1	THCA
KIT	KIRC
KRAS	PAAD, STAD
MAP3K1	STAD, THCA
MECOM	LUSC, PCPG, UVM
MTOR	LGG, PCPG
NCOR1	BRCA, COADREAD, KICH, KIRP, LAML, LIHC, PCPG, THCA
NF1	KICH, KIRP, PCPG
NFE2L2	KICH, THCA
NOTCH1	KIRC
NRAS	LGG, PCPG, THCA, UCEC
NSD1	KICH, KIRC, PRAD, THYM
PBRM1	HNSC, KIRC, MESO, PCPG, THYM, UVM
PDGFRA	KICH
PIK3CA	HNSC, KIRP, LUSC, PCPG, UVM
PIK3R1	THYM
POLQ	BRCA, HNSC
PTEN	LGG, PRAD, SARC, SKCM
RB1	COADREAD, LGG, LIHC, PRAD, SARC
SETBP1	COADREAD, HNSC, PRAD
SETD2	HNSC, KIRC, PCPG, UVM
SF3B1	KICH
SMAD4	COADREAD, THYM
SPOP	KICH, KIRP, THCA, THYM
STAG2	THCA
TET2	MESO

TP53	BRCA, COADREAD, LGG, LIHC, LUAD, SKCM, UCEC

Table S3. Number of mutant and wild-type samples as well as F1 score of classification based on ARID1A alterations across selected tumour types and their combination

Tumour Type	Number of Mutant Samples	Number of Wild-type Samples	F1 Score for Individual Tumour Type	F1 Score for the Combined Set
KICH	12	12	0.83	0.83
KIRP	59	59	0.81	0.86
LGG	207	207	0.92	0.93
PCPG	57	57	0.89	0.92
THYM	10	10	0.90	0.73
UVM	29	29	0.90	0.92
Combined Set of Above	374	374	-	0.91

Table S4. Number of mutant and wild-type samples as well as F1 score of classification based on BRAF alterations across selected tumour types and their combination

Tumour Type	Number of Mutant Samples	Number of Wild-type Samples	F1 Score for Individual Tumour Type	F1 Score for the Combined Set
COADREAD	56	56	0.86	0.85
THCA	198	198	0.93	0.95
Combined Set of Above	254	254	-	0.93

Table S5. Number of mutant and wild-type samples as well as F1 score of classification based on BRCA1 alterations across selected tumour types and their combination

Tumour Type	Number of Mutant Samples	Number of Wild-type Samples	F1 Score for Individual Tumour Type	F1 Score for the Combined Set
KICH	16	16	0.84	0.87
KIRP	91	91	0.87	0.87
UCEC	79	79	0.81	0.77
Combined Set of Above	186	186	-	0.83

Table S6. Number of mutant and wild-type samples as well as F1 score of classification based on CDH1 alterations across selected tumour types and their combination

Tumour Type	Number of Mutant Samples	Number of Wild-type Samples	F1 Score for Individual Tumour Type	F1 Score for the Combined Set

KIRP	148	127	0.87	0.85
LIHC	171	182	0.81	0.82
PRAD	150	332	0.83	0.80
SARC	157	118	0.77	0.79
THYM	15	104	0.75	0.83
UCEC	75	88	0.80	0.82
UVM	22	64	0.86	0.79
Combined Set of Above	738	1015	-	0.83

Table S7. Number of mutant and wild-type samples as well as F1 score of classification based on CTNNB1 alterations across selected tumour types and their combination

**Tumour Type**	**Number of Mutant Samples**	**Number of Wild-type Samples**	**F1 Score for Individual Tumour Type**	**F1 Score for the Combined Set**
HNSC	127	127	0.89	0.84
KIRC	38	38	0.81	0.82
PCPG	65	65	0.83	0.89
UVM	39	39	0.94	0.94
Combined Set of Above	269	269	-	0.86

Table S8. Number of mutant and wild-type samples as well as F1 score of classification based on EGFR alterations across selected tumour types and their combination

**Tumour Type**	**Number of Mutant Samples**	**Number of Wild-type Samples**	**F1 Score for Individual Tumour Type**	**F1 Score for the Combined Set**
COADREAD	281	149	0.76	0.81
HNSC	242	252	0.73	0.77
KIRP	169	107	0.86	0.89
LGG	130	369	0.83	0.82
STAD	215	190	0.73	0.77
THCA	18	464	0.73	0.55
Combined Set of Above	1055	1531	-	0.85

Table S9. Number of mutant and wild-type samples as well as F1 score of classification based on EZH2 alterations across selected tumour types and their combination

**Tumour Type**	**Number of Mutant Samples**	**Number of Wild-type Samples**	**F1 Score for Individual Tumour Type**	**F1 Score for the Combined Set**
COADREAD	239	188	0.76	0.78
KIRP	167	109	0.85	0.88

LGG	176	323	0.84	0.82
THCA	21	460	0.73	0.63
THYM	20	99	0.73	0.74
Combined Set of Above	623	1179	-	0.86

Table S10. Number of mutant and wild-type samples as well as F1 score of classification based on KDM6A alterations across selected tumour types and their combination

Tumour Type	Number of Mutant Samples	Number of Wild-type Samples	F1 Score for Individual Tumour Type	F1 Score for the Combined Set
KIRP	129	147	0.74	0.78
UCEC	77	82	0.73	0.73
Combined Set of Above	206	229	-	0.76

Table S11. Number of mutant and wild-type samples as well as F1 score of classification based on NRAS alterations across selected tumour types and their combination

Tumour Type	Number of Mutant Samples	Number of Wild-type Samples	F1 Score for Individual Tumour Type	F1 Score for the Combined Set
LGG	198	302	0.94	0.94
PCPG	119	43	0.75	0.75
Combined Set of Above	317	345	-	0.92

Table S12. Number of mutant and wild-type samples as well as F1 score of classification based on PBRM1 alterations across selected tumour types and their combination

Tumour Type	Number of Mutant Samples	Number of Wild-type Samples	F1 Score for Individual Tumour Type	F1 Score for the Combined Set
HNSC	122	122	0.84	0.89
KIRC	35	35	0.83	0.87
MESO	28	28	0.80	0.80
PCPG	66	66	0.84	0.90
THYM	15	15	0.90	0.70
UVM	40	40	0.94	0.96
Combined Set of Above	306	306	-	0.88

Table S13. Number of mutant and wild-type samples as well as F1 score of classification based on PIK3CA alterations across selected tumour types and their combination

Tumour Type	Number of Mutant Samples	Number of Wild-type Samples	F1 Score for Individual Tumour Type	F1 Score for the Combined Set
KIRP	103	173	0.81	0.79
PCPG	96	65	0.87	0.85
UVM	47	40	0.93	0.95
Combined Set of Above	246	278	-	0.84

Table S14. Number of mutant and wild-type samples as well as F1 score of classification based on PTEN alterations across selected tumour types and their combination

Tumour Type	Number of Mutant Samples	Number of Wild-type Samples	F1 Score for Individual Tumour Type	F1 Score for the Combined Set
LGG	120	380	0.86	0.86
PRAD	165	318	0.81	0.80
SARC	154	124	0.76	0.75
SKCM	245	132	0.79	0.80
Combined Set of Above	684	954	-	0.84

Table S15. Number of mutant and wild-type samples as well as F1 score of classification based on SETBP1 alterations across selected tumour types and their combination

Tumour Type	Number of Mutant Samples	Number of Wild-type Samples	F1 Score for Individual Tumour Type	F1 Score for the Combined Set
COADREAD	97	97	0.83	0.89
HNSC	212	212	0.79	0.83
PRAD	123	123	0.83	0.80
Combined Set of Above	432	432	-	0.83

Table S16. Number of mutant and wild-type samples as well as F1 score of classification based on SETD2 alterations across selected tumour types and their combination

Tumour Type	Number of Mutant Samples	Number of Wild-type Samples	F1 Score for Individual Tumour Type	F1 Score for the Combined Set
HNSC	123	123	0.87	0.88
KIRC	37	37	0.82	0.84
PCPG	67	67	0.84	0.87
UVM	38	38	0.95	0.94
Combined Set of Above	265	265	-	0.88

Table S17. Number of mutant and wild-type samples as well as F1 score of classification based on SPOP alterations across selected tumour types and their combination

Tumour Type	Number of Mutant Samples	Number of Wild-type Samples	F1 Score for Individual Tumour Type	F1 Score for the Combined Set
KICH	16	16	0.87	0.66
KIRP	87	87	0.88	0.88
THCA	21	21	0.83	0.93
THYM	14	14	0.79	0.77
Combined Set of Above	138	138	-	0.85

Table S18. Number of mutant and wild-type samples as well as F1 score of classification based on ARID1A alterations across selected tumour types and their combination after excluding THYM samples

Tumour Type	Number of Mutant Samples	Number of Wild-type Samples	F1 Score for Individual Tumour Type	F1 Score for the Combined Set
KICH	12	12	0.83	0.78
KIRP	59	59	0.81	0.86
LGG	207	207	0.92	0.92
PCPG	57	57	0.89	0.93
UVM	29	29	0.90	0.90
Combined Set of Above	364	364	-	0.90

Table S19. Number of mutant and wild-type samples as well as F1 score of classification based on EGFR alterations across selected tumour types and their combination after excluding THCA samples

Tumour Type	Number of Mutant Samples	Number of Wild-type Samples	F1 Score for Individual Tumour Type	F1 Score for the Combined Set
COADREAD	281	149	0.76	0.80
HNSC	242	252	0.73	0.76
KIRP	169	107	0.86	0.87
LGG	130	369	0.83	0.82
STAD	215	190	0.73	0.77
Combined Set of Above	1037	1067	-	0.82

Table S20. Number of mutant and wild-type samples as well as F1 score of classification based on EZH2 alterations across selected tumour types and their combination after excluding THCA samples

Tumour Type	Number of Mutant Samples	Number of Wild-type Samples	F1 Score for Individual Tumour Type	F1 Score for the Combined Set
COADREAD	239	188	0.76	0.83
KIRP	167	109	0.85	0.89
LGG	176	323	0.84	0.82
THYM	20	99	0.73	0.80
Combined Set of Above	602	719	-	0.85

Table S21. Number of mutant and wild-type samples as well as F1 score of classification based on PBRM1 alterations across selected tumour types and their combination after excluding THYM samples

Tumour Type	Number of Mutant Samples	Number of Wild-type Samples	F1 Score for Individual Tumour Type	F1 Score for the Combined Set
HNSC	122	122	0.84	0.86
KIRC	35	35	0.83	0.83
MESO	28	28	0.80	0.84
PCPG	66	66	0.84	0.87
UVM	40	40	0.94	0.97
Combined Set of Above	291	291	-	0.87

Table S22. Number of mutant and wild-type samples as well as F1 score of classification based on SPOP alterations across selected tumour types and their combination after excluding KICH samples

Tumour Type	Number of Mutant Samples	Number of Wild-type Samples	F1 Score for Individual Tumour Type	F1 Score for the Combined Set
KIRP	87	87	0.88	0.87
THCA	21	21	0.83	0.93
THYM	14	14	0.79	0.85
Combined Set of Above	122	122	-	0.88

Table S23. The chromosome coordinates used to exclude genes in close proximity to the genes of interest

Gene under Investigation	Chromosome	Start	End
APC	N/A (Only SNVs/INDELs were used for this gene)		
AR	N/A (No genes/regions were excluded)		
ARID1A	1	1	123400000
ASXL1	20	30400001	64444167
ATM	11	63600001	135086622
ATR	3	113700001	198295559
ATRX	N/A (Only SNVs/INDELs were used for this gene)		
BRAF	N/A (Only SNVs/INDELs were used for this gene)		
BRCA1	17	1	83257441
BRCA2	13	27200001	50300000
CDH1	16	1	90338345
CDK12	17	1	83257441
CDKN2A	9	18500001	28000000
CTCF	16	38400001	90338345
CTNNB1	3	2800001	79800000
EGFR	7	1	159345973
EP300	22	17400001	50818468
ERBB4	2	196600001	242193529
EZH2	7	1	159345973
FBXW7	4	97900001	190214555
FLT3	13	27200001	28300000
GATA3	10	3800001	17300000
KDM6A	N/A (No genes/regions were excluded)		
KEAP1	N/A (No genes/regions were excluded)		
KIT	4	48200001	145900000
KRAS	N/A (Only SNVs/INDELs were used for this gene)		
MAP3K1	5	1	93000000
MECOM	3	100300001	198295559
MTOR	1	1	115500000
NCOR1	17	1	22700000
NF1	17	27400001	83257441
NFE2L2	2	118100001	242193529
NOTCH1	9	1	138394717
NRAS	1	1	115500000
NSD1	5	1	181538259
PBRM1	3	4000001	79800000
PDGFRA	N/A (No genes/regions were excluded)		
PIK3CA	3	100300001	198295559
PIK3R1	5	1	181538259
POLQ	3	100300001	198295559
PTEN	10	68800001	133797422
RB1	13	18900001	61800000
SETBP1	18	21500001	80373285
SETD2	3	2800001	79800000
SF3B1	N/A (No genes/regions were excluded)		
SMAD4	18	8500001	80373285
SPOP	17	1	83257441

STAG2	N/A (No genes/regions were excluded)		
TET2	4	87100001	186200000
TP53	N/A (Only SNVs/INDELs were used for this gene)		

Table S24. List of top genes in classification of APC alterations, their importance scores, and retrieved associations with APC

Gene	Score	Known Associations with APC	Ref
LY6G6F-LY6G6D ENSG00000250641	0.0119	LY6G6D is an antigen that is overexpressed in MSS CRCs when compared to other solid tumours	28
RNF43 ENSG00000108375	0.0097	Genetic inactivation or reduced RNA levels of this gene is observed in APC wild-type CRCs	29
CEL ENSG00000170835	0.0071	Not known	
POU5F1B ENSG00000212993	0.0071	Wnt/beta-catenin was shown to be associated with this gene	30,31
LY6G6D ENSG00000244355	0.0067	LY6G6D is an antigen that is overexpressed in MSS CRCs when compared to other solid tumours	28
ASCL2 ENSG00000183734	0.0062	A transcription factor that is regulated by Wnt signal	32
AC124067.4 ENSG00000254290	0.0061	lncRNA that was shown to have some role in tumor microenvironment infiltration, cancer stemness, and drug resistance in COAD	33
PLAGL2 ENSG00000126003	0.0057	Its overexpression was shown to downregulate APC, while its knockdown led to APC upregulation	34
PLA2G12B ENSG00000138308	0.0054	Not known	
AXIN2 ENSG00000168646	0.0049	Degrades Beta-catenin in Wnt Signalling pathway and forms a protein complex with APC	35
DPEP1 ENSG00000015413	0.0046	Not known	
ZNRF3 ENSG00000183579	0.0045	Is inactivated in APC wild-type CRCs as an alternate mechanism to activate Wnt signalling pathway	36
RUBCNL ENSG00000102445	0.0042	Not known	
SLC5A6 ENSG00000138074	0.0040	Not known	
SPACA3 ENSG00000141316	0.0040	Not known	

Table S25. List of top genes in classification of ARID1A alterations, their importance scores, and retrieved associations with ARID1A

Gene	Score	Known Associations with ARID1A	Ref
RPL22L1 ENSG00000163584	0.0257	ARID1A mutant tumours were shown to be genetically dependent to RPL22L1	37

HMGN2P5 ENSG00000234664	0.0134	Not known	
TRIM67 ENSG00000119283	0.0111	Not known	
KCNIP2 ENSG00000120049	0.0100	Not known	
RUNDC3A-AS1 ENSG00000267750	0.0096	Not known	
AL109810.2 ENSG00000235710	0.0087	Not known	
AC068057.2 ENSG00000228528	0.0067	Not known	
AC005740.1 ENSG00000226040	0.0067	Not known	
NFATC1 ENSG00000131196	0.0060	Is a downstream target of ARID1A which is activated by it. Also, plays important transcriptional roles in absence of ARID1A. Its overexpression leads to dissociation of ARID1A from the genome	38,39
TMEM147 ENSG00000105677	0.0059	Not known	
DRG2 ENSG00000108591	0.0058	Not known	
ATCAY ENSG00000167654	0.0053	Not known	
AC124798.1 ENSG00000260196	0.0053	Not known	
CLVS1 ENSG00000177182	0.0044	Not known	
PAX5 ENSG00000196092	0.0043	Not known	

Table S26. List of top genes in classification of ATR alterations, their importance scores, and retrieved associations with ATR

Gene	Score	Known Associations with ATR	Ref
TFG ENSG00000114354	0.0124	Not known	
FAM83D ENSG00000101447	0.0067	Not known	
UBE2C ENSG00000175063	0.0067	Its expression has a strong negative correlation with ATR expression. Also, it regulates PER1 which interacts with ATR as a negative regulator of cell division	40,41
MYBL2 ENSG00000101057	0.0065	Interacts with ATR to exit from S phase into G2 during cell division	42
CDC20 ENSG00000117399	0.0063	Not known	
CKS1B ENSG00000173207	0.0058	Not known	

TUBA1C ENSG00000167553	0.0056	Not known	
TROAP ENSG00000135451	0.0054	Not known	
LINC01633 ENSG00000260976	0.0052	Not known	
ACAA1 ENSG00000060971	0.0051	Was shown to be negatively correlated with ATR in lung cancer	43
AURKA ENSG00000087586	0.0051	Its overexpression was shown to be associated with downregulation of ATR in colon cancer	44
CENPA ENSG00000115163	0.0048	It was shown that ATR is needed for proper localization of the proteins produced by this gene	45
TRIP13 ENSG00000071539	0.0045	Its knockdown is associated with increased p53 phosphorylation which is a primary target of ATR	46
BIRC5 ENSG00000089685	0.0040	Not known	
TMEM189 ENSG00000240849	0.0040	Not known	

Table S27. List of top genes in classification of ATRX alterations, their importance scores, and retrieved associations with ATRX

Gene	Score	Known Associations with ATRX	Ref
DRG2 ENSG00000108591	0.0519	In IDH-mutant gliomas, high mutation rate of ATRX and downregulation of DRG2 have been observed	47
AC068057.2 ENSG00000228528	0.0394	Not known	
EDA2R ENSG00000131080	0.0343	Not known	
OR4N2 ENSG00000176294	0.0317	Not known	
OR4K6P ENSG00000228304	0.0292	Not known	
TERT ENSG00000164362	0.0279	It is significantly downregulated with partial loss of ATRX activity	48
TRIP4 ENSG00000103671	0.0190	Not known	
DEFB119 ENSG00000180483	0.0183	Not known	
TPTEP1 ENSG00000100181	0.0182	Not known	
GRPEL2 ENSG00000164284	0.0181	Not known	
FDXR ENSG00000161513	0.0156	Mutations in ATRX were shown to be positively associated with FDXR expression	49
STOX1 ENSG00000165730	0.0143	Not known	
PTCHD4 ENSG00000244694	0.0129	Not known	

REM1 ENSG00000088320	0.0120	Not known	
CD81-AS1 ENSG00000238184	0.0111	Not known	

Table S28. List of top genes in classification of BRAF alterations, their importance scores, and retrieved associations with BRAF

Gene	Score	Known Associations with BRAF	Ref
DCSTAMP ENSG00000164935	0.0364	Is overexpressed in presence of BRAF mutations in thyroid cancer	50,51
TMPRSS6 ENSG00000187045	0.0262	Is overexpressed in presence of BRAF mutations in thyroid cancer	52
NECTIN4 ENSG00000143217	0.0214	Is overexpressed in presence of BRAF mutations in melanoma	53
ERBB3 ENSG00000065361	0.0166	Is one of the most potent activators of AKT pathway and a key factor in development of resistance to BRAF and MEK inhibitors	54
KLK10 ENSG00000129451	0.0163	Is hypomethylated in thyroid tumours with mutated BRAF	55
PNPLA5 ENSG00000100341	0.0154	Not known	
PDLIM4 ENSG00000131435	0.0149	Is overexpressed in presence of BRAF mutations in thyroid cancer	56
EPHA10 ENSG00000183317	0.0142	Is significantly downregulated in thyroid tumours with wild-type BRAF	51
KLK7 ENSG00000169035	0.0117	Is overexpressed in presence of BRAF mutations in thyroid cancer	57
TACSTD2 ENSG00000184292	0.0103	Is overexpressed in presence of BRAF mutations in thyroid cancer	58
FN1 ENSG00000115414	0.0102	Is differentially expressed between BRAF-wt and BRAF-mut samples in thyroid cancer	59
LAD1 ENSG00000159166	0.0097	Is induced by BRAF V600E in thyroid cancer	56
CRLF2 ENSG00000205755	0.0094	Not known	
SYT12 ENSG00000173227	0.0091	It is associated with BRAF mutated thyroid tumours	60
SLC34A2 ENSG00000157765	0.0074	Is overexpressed in BRAF mutant and underexpressed in BRAF wild-type thyroid tumours	51,61

Table S29. List of top genes in classification of BRCA1 alterations, their importance scores, and retrieved associations with BRCA1

Gene	Score	Known Associations with BRCA1	Ref
TBRG4 ENSG00000136270	0.0142	Is a regulator of TGF-beta which interacts with BRCA1 and can block or enhance BRCA1 activity	62,63

CGNL1<br>ENSG00000128849	0.0055	Not known	
CHCHD7<br>ENSG00000170791	0.0046	Not known	
NQO1<br>ENSG00000181019	0.0043	BRCA1 knockdown was shown to decrease NQO1 expression. Also, NRF2 was shown to interact with BRCA1 which is a direct regulator of NQO1	64,65
TSKU<br>ENSG00000182704	0.0040	Not known	
BLNK<br>ENSG00000095585	0.0039	Is downregulated in absence of BRCA1	66
MAP6<br>ENSG00000171533	0.0037	Not known	
CCNG1<br>ENSG00000113328	0.0036	Not known	
ABHD11<br>ENSG00000106077	0.0035	Not known	
NSUN5<br>ENSG00000130305	0.0035	Not known	
PDCD4<br>ENSG00000150593	0.0032	Not known	
PNMA3<br>ENSG00000183837	0.0028	Not known	
NDUFB4P11<br>ENSG00000259374	0.0026	Not known	

Table S30. List of top genes in classification of CDH1 alterations, their importance scores, and retrieved associations with CDH1

Gene	Score	Known Associations with CDH1	Ref
IGF2BP1<br>ENSG00000159217	0.0041	Frequently interacts with CDH1. Also, it leads to overexpression of LEF1 and SNAI2 which are negative regulators of CDH1	67,68
CIRBP<br>ENSG00000099622	0.0033	Not known	
ABTB3<br>ENSG00000151136	0.0027	Not known	
IQGAP3<br>ENSG00000183856	0.0026	Not known, but there are some associations between CDH1 and IQGAP1 and IQGAP2	69,70
SYTL1<br>ENSG00000142765	0.0023	Not known	
JMJD7<br>ENSG00000243789	0.0023	Not known	
EDN3<br>ENSG00000124205	0.0022	Not known	
SPATA18<br>ENSG00000163071	0.0022	Not known	
ENTREP1<br>ENSG00000135063	0.0020	Not known	

CNIH4 ENSG00000143771	0.0019	Not known	
PDCD4 ENSG00000150593	0.0018	Its knockdown leads to downregulation of CDH1	71

Table S31. List of top genes in classification of CDKN2A alterations, their importance scores, and retrieved associations with CDKN2A

Gene	Score	Known Associations with CDKN2A	Ref
UBE2C ENSG00000175063	0.0086	High expression of this gene is positively correlated with CDKN2A expression	72
TROAP ENSG00000135451	0.0080	Not known	
TPX2 ENSG00000088325	0.0079	Not known	
PLK1 ENSG00000166851	0.0075	In breast tumours with high levels of PLK1, CDKN2A was observed to be upregulated	73
BIRC5 ENSG00000089685	0.0074	In oral squamous cells carcinoma, it was observed that CDKN2A is highly involved in BIRC5 expression	74
CENPA ENSG00000115163	0.0072	Not known	
NEK2 ENSG00000117650	0.0066	Not known	
MYBL2 ENSG00000101057	0.0063	Not known	
AURKA ENSG00000087586	0.0062	Is upregulated when CDKN2A is inhibited	75
AURKB ENSG00000178999	0.0058	Is downregulated when CDKN2A is expressed	76
CDCA8 ENSG00000134690	0.0056	Is upregulated when CDKN2A is inhibited	75
CDC20 ENSG00000117399	0.0056	Its expression is positively correlated with CDKN2A expression	77
ZFPM2-AS1 ENSG00000251003	0.0050	Not known	
CDC25C ENSG00000158402	0.0047	Not known	
KIF20A ENSG00000112984	0.0047	Not known	

Table S32. List of top genes in classification of CTCF alterations, their importance scores, and retrieved associations with CTCF

Gene	Score	Known Associations with CTCF	Ref
NEK2 ENSG00000117650	0.0036	Not known	
POC1A ENSG00000164087	0.0035	Not known	

AURKA ENSG00000087586	0.0033	Its expression is positively regulated by CTCF mediated by FOXM1	78
CKS1B ENSG00000173207	0.0032	Not known	
CDC25C ENSG00000158402	0.0031	Is activated by PLK1 which is phosphorylated by AURKA (associated with CTCF as mentioned)	79,80
GPRASP1 ENSG00000198932	0.0029	Not known	
CCNB1 ENSG00000134057	0.0025	Is activated by PLK1 which is phosphorylated by AURKA (associated with CTCF as mentioned)	78
PKMP3 ENSG00000220563	0.0024	Not known	
EARS2 ENSG00000103356	0.0023	Not known	
AGAP2 ENSG00000135439	0.0022	Not known	
RPL39L ENSG00000163923	0.0020	Not known	
SNRPN ENSG00000128739	0.0020	CTCF binding seems to have a role in regulation of SNRPN as a part of imprinting process	81
TK1 ENSG00000167900	0.0019	Not known	
ZNF154 ENSG00000179909	0.0017	Not known	
KIF20A ENSG00000112984	0.0017	Not known	

Table S33. List of top genes in classification of CTNNB1 alterations, their importance scores, and retrieved associations with CTNNB1

Gene	Score	Known Associations with CTNNB1	Ref
RPSA2 ENSG00000225178	0.0197	Not known	
MORC2 ENSG00000133422	0.0110	It positively regulates CTNNB1 expression and Beta-catenin signaling pathway	82
RPL14P1 ENSG00000139239	0.0087	Not known	
IPO9 ENSG00000198700	0.0068	Not known	
ABCB6 ENSG00000115657	0.0052	Its depletion was shown to decrease CTNNB1 expression	83
RPL15P3 ENSG00000212802	0.0051	Not known	
C3orf38 ENSG00000179021	0.0044	Not known	
PANX2 ENSG00000073150	0.0042	Not known, but overexpression of PANX1 was shown to be correlated with CTNNB1 upregulation	84
MSL3B ENSG00000224287	0.0037	Not known	

TTYH3<br>ENSG00000136295	0.0037	Its overexpression was shown to be associated with upregulation of CTNNB1 and Beta-catenin signaling pathway	85
CLPTM1L<br>ENSG00000049656	0.0035	Not known	
SLC35B1<br>ENSG00000121073	0.0033	Not known	
JAG2<br>ENSG00000184916	0.0032	Not known	
PCGF1<br>ENSG00000115289	0.0030	Overexpression of Hoxa9, a direct target of PCGF1, was shown to be associated with upregulation of CTNNB1	86
KRT10-AS1<br>ENSG00000167920	0.0030	Not known	

Table S34. List of top genes in classification of EGFR alterations, their importance scores, and retrieved associations with EGFR

Gene	Score	Known Associations with EGFR	Ref
OPN3<br>ENSG00000054277	0.0061	Not known	
GALNT3<br>ENSG00000115339	0.0052	Its knockdown was shown to alter EGFR glycosylation which can lead to increased activation	87
MIR9-3HG<br>ENSG00000255571	0.0049	Not known	
SH2D4A<br>ENSG00000104611	0.0049	Not known	
IGF2BP2<br>ENSG00000073792	0.0045	EGFR expression was shown to be positively associated with IGF2BP2	88,89
SNHG11<br>ENSG00000174365	0.0043	Not known	
KLRC4<br>ENSG00000183542	0.0040	Not known	
ERGIC3<br>ENSG00000125991	0.0035	Not known	
FNDC11<br>ENSG00000125531	0.0032	Not known	
CD58<br>ENSG00000116815	0.0030	Not known	
F11R<br>ENSG00000158769	0.0030	Not known	
AC068775.1<br>ENSG00000255641	0.0028	Not known	
KLRC3<br>ENSG00000205810	0.0027	Not known	
LINC02253<br>ENSG00000259485	0.0025	Not known	
SMOC1<br>ENSG00000198732	0.0025	Is downregulated in presence of EGFR 19Del mutation and is upregulated in presence of EGFR L858R mutation	90

Table S35. List of top genes in classification of EZH2 alterations, their importance scores, and retrieved associations with EZH2

Gene	Score	Known Associations with EZH2	Ref
TMEM54 ENSG00000121900	0.0086	Not known	
SNHG18 ENSG00000250786	0.0065	Not known	
VAV3 ENSG00000134215	0.0046	Its expression was shown to be associated with EZH2 mutational status	91
LRRC36 ENSG00000159708	0.0041	Not known	
KCNK5 ENSG00000164626	0.0039	Not known	
GSS ENSG00000100983	0.0035	Not known	
GPR27 ENSG00000170837	0.0034	Not known	
KIF9 ENSG00000088727	0.0030	Not known	
DPEP1 ENSG00000015413	0.0028	Not known	
UBAC2 ENSG00000134882	0.0026	Not known	
PHETA2 ENSG00000177096	0.0025	Not known	
HNF4G ENSG00000164749	0.0025	Transcriptionally activates lncRNA-DAW which directly interacts with EZH2 and leads to its degradation	92
C3orf85 ENSG00000241224	0.0024	Not known	
TMT1B ENSG00000170439	0.0023	Not known	
TNFRSF11B ENSG00000164761	0.0021	Not known	

Table S36. List of top genes in classification of FBXW7 alterations, their importance scores, and retrieved associations with FBXW7

Gene	Score	Known Associations with FBXW7	Ref
MYBL2 ENSG00000101057	0.0050	Not known	
UBE2C ENSG00000175063	0.0048	Not known	
AURKA ENSG00000087586	0.0047	Is negatively regulated and can be degraded by FBXW7	93,94
TRIP13 ENSG00000071539	0.0046	Directly binds to the promoter of FBXW7 and inhibits its transcription	95,96
ALG3 ENSG00000214160	0.0042	Not known	

MTFR2 ENSG00000146410	0.0041	Not known	
TROAP ENSG00000135451	0.0041	Not known	
FAM83D ENSG00000101447	0.0040	Directly binds to FBXW7 and downregulates its expression	97,98
TTK ENSG00000112742	0.0039	FBXW7-deficient cells were shown to be vulnerable to TTK knockdown. Also, loss of FBXW7 was demonstrated to increase ANXA2 expression which regulates TTK expression	99,100
TPX2 ENSG00000088325	0.0035	Not known	
NUF2 ENSG00000143228	0.0031	Was shown to be upregulated when FBXW7 was silenced	101
NEK2 ENSG00000117650	0.0031	Is negatively regulated by circ-FBXW7 (FBXW7 circular RNA)	102
TMEM229B ENSG00000198133	0.0030	Not known	
NUDT9 ENSG00000170502	0.0027	Not known	
CENPA ENSG00000115163	0.0027	FBXW7 loss of function leads to phosphorylation of CENPA and its reduced level at centromeres	103

Table S37. List of top genes in classification of GATA3 alterations, their importance scores, and retrieved associations with GATA3

Gene	Score	Known Associations with GATA3	Ref
AURKA ENSG00000087586	0.0065	GATA3 binds to its promoter and positively regulates it	104
UBE2C ENSG00000175063	0.0062	Its expression was shown to be positively correlated with GATA3	105
CENPA ENSG00000115163	0.0058	Not known	
CDC20 ENSG00000117399	0.0052	It has been shown that GATA3 mutations are associated with downregulation of CDC20 and GATA3 expression is negatively associated with CDC20 overexpression	106
TPX2 ENSG00000088325	0.0050	Not known	
NEK2 ENSG00000117650	0.0049	Not known	
NUF2 ENSG00000143228	0.0041	Its expression was shown to be positively correlated with GATA3	107, 108
BIRC5 ENSG00000089685	0.0041	Not known	
ARMH3 ENSG00000120029	0.0038	Not known	
CDCA8 ENSG00000134690	0.0037	Was shown to be a target of GATA3 and to be regulated by it	109

KIF2C ENSG00000142945	0.0036	Not known	
TRIP13 ENSG00000071539	0.0031	Not known	
KIF4A ENSG00000090889	0.0029	Not known	
SGO1 ENSG00000129810	0.0029	Not known	
MYBL2 ENSG00000101057	0.0026	Not known	

Table S38. List of top genes in classification of KDM6A alterations, their importance scores, and retrieved associations with KDM6A

Gene	Score	Known Associations with KDM6A	Ref
ABCF3 ENSG00000161204	0.0066	Not known	
ACAD9 ENSG00000177646	0.0058	Not known	
EPB41L4A-AS1 ENSG00000224032	0.0050	Not known	
TIMMDC1 ENSG00000113845	0.0048	Not known	
MRPS22 ENSG00000175110	0.0040	Not known	
EEF2 ENSG00000167658	0.0038	Not known	
SNHG8 ENSG00000269893	0.0038	Not known	
RGS3 ENSG00000138835	0.0035	Not known	
MIX23 ENSG00000160124	0.0035	Not known	
EEF1A1 ENSG00000156508	0.0035	Not known	
TUFM ENSG00000178952	0.0034	Not known	
ALG3 ENSG00000214160	0.0034	Not known	
CLCN2 ENSG00000114859	0.0033	Not known	
PNMA3 ENSG00000183837	0.0028	Not known	
LSG1 ENSG00000041802	0.0027	Not known	

Table S39. List of top genes in classification of KEAP1 alterations, their importance scores, and retrieved associations with KEAP1

Gene	Score	Known Associations with KEAP1	Ref
DIRAS3 ENSG00000162595	0.0161	Its expression was shown to be associated with KEAP1 accumulation	110
AHNAK2 ENSG00000185567	0.0153	Not known	
PRSS2 ENSG00000275896	0.0126	Not known	
PTPRE ENSG00000132334	0.0121	Not known	
MFGE8 ENSG00000140545	0.0118	Upregulates NRF2 activation and NRF2 is a direct target of KEAP1	111
PPP1R1B ENSG00000131771	0.0116	Knockdown of NRF2 (target of KEAP1) was shown to be associated with PPP1R1B downregulation	112
PROS1 ENSG00000184500	0.0111	Not known	
MRPS12 ENSG00000128626	0.0097	Not known	
ERFE ENSG00000178752	0.0095	It was shown to be downregulated in NRF2 (target of KEAP1) deficient mice	113
ACSL5 ENSG00000197142	0.0093	Not known	
EIF4A2 ENSG00000156976	0.0081	Inactivation of KEAP1 was shown to confer resistance to EIF4A inhibition. Also, NRF2 (target of KEAP1) gene products can improve EIF4A-dependent transcripts translation	114, 115
STXBP2 ENSG00000076944	0.0081	Not known	
SP100 ENSG00000067066	0.0080	A component of promyelocytic leukemia-nuclear body which plays a role in regulating NRF2 (target of KEAP1) stability	116
PTK2B ENSG00000120899	0.0075	Not known	
PRICKLE1 ENSG00000139174	0.0068	Not known	

Table S40. List of top genes in classification of KIT alterations, their importance scores, and retrieved associations with KIT

Gene	Score	Known Associations with KIT	Ref
TROAP ENSG00000135451	0.0063	Not known	
AURKA ENSG00000087586	0.0062	Not known	
UBE2C ENSG00000175063	0.0055	Not known	
TPX2 ENSG00000088325	0.0044	Not known	
MTFR2 ENSG00000146410	0.0040	Not known	

MYBL2 ENSG00000101057	0.0039	Not known	
TTK ENSG00000112742	0.0036	Not known	
TRIP13 ENSG00000071539	0.0033	Not known	
NUF2 ENSG00000143228	0.0032	Not known	
GATB ENSG00000059691	0.0031	Not known	
NEK2 ENSG00000117650	0.0028	Not known	
BIRC5 ENSG00000089685	0.0028	Is highly expressed in KIT D816V mutated cells and confers resistance to TNF in these cells	117
SKA1 ENSG00000154839	0.0028	Not known	
CBR4 ENSG00000145439	0.0028	Not known	
FAM83D ENSG00000101447	0.0027	Not known	

Table S41. List of top genes in classification of KRAS alterations, their importance scores, and retrieved associations with KRAS

Gene	Score	Known Associations with KRAS	Ref
AC009065.3 ENSG00000259933	0.0093	Not known	
BCL2L15 ENSG00000188761	0.0092	Was shown to be upregulated in presence of KRAS and PIK3CA mutations and KRAS mutational status was shown to be associated with the diagnostic predictive power of BCL2L15 expression	118
AL049836.1 ENSG00000258919	0.0091	Not known	
ERN2 ENSG00000134398	0.0087	Not known	
APOBEC1 ENSG00000111701	0.0079	Subsets of APOBEC1-edited transcripts encode proteins that play a role in KRAS-related cellular pathways	119
GPR35 ENSG00000178623	0.0079	Not known	
LINC02086 ENSG00000244649	0.0073	Not known	
CEACAM6 ENSG00000086548	0.0070	Its overexpression was shown to be associated with KRAS mutations, and knockdown in KRAS mutant samples inhibited tumour growth. Also, it was shown that CEACAM6 is associated with or dependent on KRAS	120, 121
AL355312.4 ENSG00000273132	0.0059	Not known	
CLDN10-AS1 ENSG00000223392	0.0058	Not known	

AC008870.5 ENSG00000278912	0.0045	Not known	
MST1R ENSG00000164078	0.0044	In KRAS mutant sample, its expression was shown to mediate tumour growth. Also, it was shown that MST1R is associated with or dependent on KRAS	121, 122
EPS8L3 ENSG00000198758	0.0040	Not known	
PPP1R14D ENSG00000166143	0.0037	Not known	
CEACAM5 ENSG00000105388	0.0035	Its overexpression was shown to be associated with KRAS mutations	123

Table S42. List of top genes in classification of MAP3K1 alterations, their importance scores, and retrieved associations with MAP3K1

**Gene**	**Score**	**Known Associations with MAP3K1**	**Ref**
UBE2C ENSG00000175063	0.0048	Not known	
FAM83D ENSG00000101447	0.0047	Not known	
AURKA ENSG00000087586	0.0042	Not known	
NEK2 ENSG00000117650	0.0039	Not known	
CENPA ENSG00000115163	0.0036	Not known	
MYBL2 ENSG00000101057	0.0035	Not known	
CDC20 ENSG00000117399	0.0035	Was shown to be upregulated when MAP3K1 was highly expressed	124
HARS1 ENSG00000170445	0.0034	Not known	
CDCA3 ENSG00000111665	0.0033	Not known	
TROAP ENSG00000135451	0.0032	Not known	
MTFR2 ENSG00000146410	0.0032	Not known	
MED7 ENSG00000155868	0.0032	Not known	
CKS1B ENSG00000173207	0.0031	Not known	
CDC25C ENSG00000158402	0.0029	Is downregulated when MAP3K1 is inhibited and upregulated with MAP3K1 is highly expressed	124, 125
BIRC5 ENSG00000089685	0.0028	Is downregulated when MAP3K1 is mutated	126

Table S43. List of top genes in classification of NCOR1 alterations, their importance scores, and retrieved associations with NCOR1

Gene	Score	Known Associations with NCOR1	Ref
AURKA ENSG00000087586	0.0068	Not known	
ALG3 ENSG00000214160	0.0049	Not known	
NEK2 ENSG00000117650	0.0048	Not known	
UBE2C ENSG00000175063	0.0048	Not known	
TROAP ENSG00000135451	0.0048	Not known	
CDC20 ENSG00000117399	0.0043	Not known	
CENPA ENSG00000115163	0.0037	Not known	
MYBL2 ENSG00000101057	0.0036	Directly interacts with NCOR1 and is negatively regulated by NCOR1	127
CIRBP ENSG00000099622	0.0035	Was shown to interact with NCOR1 to regulate circadian rhythm	128, 129
CDCA8 ENSG00000134690	0.0035	Not known	
TK1 ENSG00000167900	0.0035	Not known	
RAB5IF ENSG00000101084	0.0033	Not known	
IQGAP3 ENSG00000183856	0.0029	Not known	
BIRC5 ENSG00000089685	0.0026	Not known	
TPX2 ENSG00000088325	0.0025	Not known	

Table S44. List of top genes in classification of NF1 alterations, their importance scores, and retrieved associations with NF1

Gene	Score	Known Associations with NF1	Ref
UBE2C ENSG00000175063	0.0048	Not known	
AURKA ENSG00000087586	0.0043	Is overexpressed in NF1 mutant tumour cells	130, 131
TRIP13 ENSG00000071539	0.0035	Not known	
CDC20 ENSG00000117399	0.0031	Is overexpressed in NF1 mutant melanoma cells	132
MYBL2 ENSG00000101057	0.0030	In MYBL2 high tumours, higher rate of NF1 downregulation and heterozygous loss was observed	133

CENPA ENSG00000115163	0.0030	Not known	
CBX7 ENSG00000100307	0.0027	Not known	
UBE2T ENSG00000077152	0.0026	Not known	
TROAP ENSG00000135451	0.0025	Not known	
NEK2 ENSG00000117650	0.0022	Not known	
CIRBP ENSG00000099622	0.0022	Not known	
TPX2 ENSG00000088325	0.0020	Not known	
KIF2C ENSG00000142945	0.0020	Not known	
CDCA8 ENSG00000134690	0.0020	Not known	
CDC25C ENSG00000158402	0.0018	Not known	

Table S45. List of top genes in classification of NOTCH1 alterations, their importance scores, and retrieved associations with NOTCH1

Gene	Score	Known Associations with NOTCH1	Ref
MICU1 ENSG00000107745	0.0129	Not known	
PPP1R12A-AS1 ENSG00000257557	0.0123	Not known	
PTH1R ENSG00000160801	0.0104	Is PTH receptor, and PTH treatment was shown to affect NOTCH signaling pathway components	134
TCTA ENSG00000145022	0.0097	Not known	
AC011352.3 ENSG00000251320	0.0081	Not known	
AC011352.1 ENSG00000248362	0.0075	Not known	
MYL3 ENSG00000160808	0.0073	Its knockdown was shown to enhance expression of NOTCH related genes and activate NOTCH signaling pathway	135
CYB5D2 ENSG00000167740	0.0068	Not known	
MAT1A ENSG00000151224	0.0067	Not known	
SPRYD4 ENSG00000176422	0.0067	Not known	
SPINK13 ENSG00000214510	0.0067	It was shown to be differentially expressed between BCAT1 (a target of NOTCH1) wild-type and BCAT1 knockout samples	136

C5orf46 ENSG00000178776	0.0061	Not known	
SLC17A1 ENSG00000124568	0.0060	Not known	
MOCOS ENSG00000075643	0.0055	Not known	
LINC02609 ENSG00000233593	0.0054	Not known	

Table S46. List of top genes in classification of NRAS alterations, their importance scores, and retrieved associations with NRAS

Gene	Score	Known Associations with NRAS	Ref
CHGB ENSG00000089199	0.0198	Not known	
CCDC146 ENSG00000135205	0.0163	Not known	
LEFTY2 ENSG00000143768	0.0129	Not known	
KRT8P30 ENSG00000224928	0.0127	Not known	
AL031658.2 ENSG00000278012	0.0124	Not known	
SEZ6L2 ENSG00000174938	0.0114	Not known	
BCL2L12 ENSG00000126453	0.0109	Not known	
AC026369.1 ENSG00000249695	0.0104	Not known	
RIIAD1 ENSG00000178796	0.0102	Not known	
SRRM3 ENSG00000177679	0.0088	Not known	
COQ8B ENSG00000123815	0.0087	Not known	
KCNK3 ENSG00000171303	0.0086	Not known	
NFATC1 ENSG00000131196	0.0081	Not known	
L1CAM ENSG00000198910	0.0080	Not known	
AC012213.4 ENSG00000271830	0.0079	Not known	

Table S47. List of top genes in classification of NSD1 alterations, their importance scores, and retrieved associations with NSD1

Gene	Score	Known Associations with NSD1	Ref

CDCA2 ENSG00000184661	0.0046	Not known	
UBE2C ENSG00000175063	0.0043	Not known	
CAMK2B ENSG00000058404	0.0037	Not known	
AURKA ENSG00000087586	0.0035	Not known	
CDC20 ENSG00000117399	0.0034	Not known	
CENPA ENSG00000115163	0.0034	Not known	
TROAP ENSG00000135451	0.0032	Not known	
CKS1B ENSG00000173207	0.0029	Not known	
MYBL2 ENSG00000101057	0.0028	Not known	
ESPL1 ENSG00000135476	0.0027	Not known	
MTFR2 ENSG00000146410	0.0027	Not known	
TMEM229B ENSG00000198133	0.0027	Not known	
BIRC5 ENSG00000089685	0.0022	Not known	
FAM83D ENSG00000101447	0.0020	Not known	
NUSAP1 ENSG00000137804	0.0018	Not known	

Table S48. List of top genes in classification of PBRM1 alterations, their importance scores, and retrieved associations with PBRM1

**Gene**	**Score**	**Known Associations with PBRM1**	**Ref**
RPSAP58 (RPSA2) ENSG00000225178	0.0190	Not known	
MORC2 ENSG00000133422	0.0117	Not known	
RPL14P1 ENSG00000139239	0.0101	Not known	
RPSAP54 ENSG00000213621	0.0075	Not known	
RPSAP19 ENSG00000183298	0.0066	Not known	
RPL29P26 ENSG00000241556	0.0062	Not known	
RPL15P3 ENSG00000212802	0.0054	Not known	

RPSAP12 ENSG00000240087	0.0042	Not known	
TRNT1 ENSG00000072756	0.0041	Not known	
RPL32P18 ENSG00000146677	0.0036	Not known	
RPSAP14 ENSG00000233984	0.0028	Not known	
ABCB6 ENSG00000115657	0.0027	Not known	
PANX2 ENSG00000073150	0.0027	Not known	
SNAPC1 ENSG00000023608	0.0027	Not known	
AC005538.2 ENSG00000279809	0.0027	Not known	

Table S49. List of top genes in classification of PIK3CA alterations, their importance scores, and retrieved associations with PIK3CA

Gene	Score	Known Associations with PIK3CA	Ref
ZNF691-DT ENSG00000228192	0.0057	Not known	
APOM ENSG00000204444	0.0052	Not known	
AC012354.1 ENSG00000225156	0.0039	Not known	
COMTD1 ENSG00000165644	0.0036	Not known	
RNF113A ENSG00000125352	0.0035	Not known	
EPB41L4A-AS1 ENSG00000224032	0.0034	Not known	
CCDC87 ENSG00000182791	0.0034	Not known	
LINC02332 ENSG00000259054	0.0030	Not known	
HCG15 ENSG00000227214	0.0027	Not known	
DANCR ENSG00000226950	0.0027	Was shown to positively regulate PIK3CA expression and activate PI3K/AKT signaling pathway	137
TMEM121 ENSG00000184986	0.0027	Not known	
TMEM82 ENSG00000162460	0.0027	Not known	
PARP3 ENSG00000041880	0.0026	Not known	
RHOD ENSG00000173156	0.0024	Not known	

VSTM2L ENSG00000132821	0.0024	Not known	

Table S50. List of top genes in classification of PTEN alterations, their importance scores, and retrieved associations with PTEN

Gene	Score	Known Associations with PTEN	Ref
CDCA8 ENSG00000134690	0.0074	Competes with PTEN for AKT binding which affects CDCA8 expression	138
AURKA ENSG00000087586	0.0074	Downregulation of PTEN was shown to increase AURKA expression and downregulation of AURKA was shown to increase PTEN expression. It was also shown that phosphorylated AURKA is a downstream target of PTEN	139
CDC20 ENSG00000117399	0.0073	Was shown to physically interact with PTEN in mitotic checkpoint complex	140
CCNB1 ENSG00000134057	0.0067	Knockdown of PTEN was shown to decrease CCNB1 expression	141
ANXA5 ENSG00000164111	0.0066	Not known	
RPA3 ENSG00000106399	0.0066	RPA was shown to function as a trimer of RPA1, RPA2, and RPA3 which forms a complex with PTEN	142, 143
AC026401.3 ENSG00000280206	0.0066	Not known	
SHOX2 ENSG00000168779	0.0065	Its high expression was shown to be correlated with PTEN mutations	144
KIF4A ENSG00000090889	0.0063	Its high expression was shown to be associated with PTEN mutations and shallow deletion	145
CCNB2 ENSG00000157456	0.0057	Its knockdown was found to decrease PTEN centrosomal levels	146
CTHRC1 ENSG00000164932	0.0047	Not known	
GP2 ENSG00000169347	0.0042	It was found to be upregulated upon PTEN loss	147
ASF1B ENSG00000105011	0.0040	Not known	
CD58 ENSG00000116815	0.0039	Low expression of PTEN was shown to be associated with CD58 downregulation	148
PIGT ENSG00000124155	0.0038	Not known	

Table S51. List of top genes in classification of RB1 alterations, their importance scores, and retrieved associations with RB1

Gene	Score	Known Associations with RB1	Ref
FAM83D ENSG00000101447	0.0109	Not known	

AURKA ENSG00000087586	0.0084	RB1 mutant cells were shown to be highly sensitive to AURKA inhibitors, with evidence showing that they rely on elevated AURKA activity for survival	149, 150
TROAP ENSG00000135451	0.0080	Not known	
CDC25C ENSG00000158402	0.0074	Not known	
UBE2C ENSG00000175063	0.0074	Its expression was shown to be negatively correlated with RB1 expression	151
BIRC5 ENSG00000089685	0.0062	RB1 was shown to contribute to the regulation of survivin (encoded by BIRC5) activity and RB1-negative cells were shown to be dependent on survivin for survival	152, 153
NEK2 ENSG00000117650	0.0058	Its knockdown was shown to be associated with higher RB1 activity	154, 155
CDCA8 ENSG00000134690	0.0057	Its overexpression was shown to be positively correlated with RB1 mutations, and it was shown to be downregulated in response to p53/RB signaling	156, 157
TPX2 ENSG00000088325	0.0052	Not known	
DEPDC1 ENSG00000024526	0.0050	Not known	
CENPA ENSG00000115163	0.0047	RB knockdown was found to increase CENPA expression	158
KIF4A ENSG00000090889	0.0047	Not known	
SGO1 ENSG00000129810	0.0039	Not known	
DSN1 ENSG00000149636	0.0036	Not known	
CCNB2 ENSG00000157456	0.0036	Not known	

Table S52. List of top genes in classification of SETBP1 alterations, their importance scores, and retrieved associations with SETBP1

Gene	Score	Known Associations with SETBP1	Ref
SPATA18 ENSG00000163071	0.0086	Not known	
RPL17P6 ENSG00000226084	0.0062	Not known	
RPL17P36 ENSG00000236058	0.0037	Not known	
RPL17P18 ENSG00000234742	0.0032	Not known	
AL157394.1 ENSG00000261438	0.0027	Not known	
IL18R1 ENSG00000115604	0.0025	Not known	

CELF5 ENSG00000161082	0.0022	Not known	
DCAF13 ENSG00000164934	0.0022	Not known	

Table S53. List of top genes in classification of SETD2 alterations, their importance scores, and retrieved associations with SETD2

Gene	Score	Known Associations with SETD2	Ref
RPSAP58 (RPSA2) ENSG0000225178	0.0188	Not known	
MORC2 ENSG00000133422	0.0098	Not known	
RPL15P3 ENSG00000212802	0.0069	Not known	
IPO9 ENSG00000198700	0.0052	Not known	
MSL3B ENSG00000224287	0.0049	Not known	
RPL14P1 ENSG00000139239	0.0041	Not known	
CIMAP1C ENSG00000182950	0.0039	Not known	
C3orf38 ENSG00000179021	0.0037	Not known	
PANX2 ENSG00000073150	0.0036	Not known	
ABCB6 ENSG00000115657	0.0030	Not known	
RPSAP12 ENSG00000240087	0.0030	Not known	
RPSAP14 ENSG00000233984	0.0029	Not known	
AC005538.2 ENSG00000279809	0.0027	Not known	
KRT10-AS1 ENSG00000167920	0.0026	Not known	
SLC29A4 ENSG00000164638	0.0025	Not known	

Table S54. List of top genes in classification of SF3B1 alterations, their importance scores, and retrieved associations with SF3B1

Gene	Score	Known Associations with SF3B1	Ref
AC022400.8 ENSG00000279088	0.0211	Not known	
IDUA ENSG00000127415	0.0148	Not known	

AP000892.3 ENSG00000276505	0.0119	Not known	
SRRM5 ENSG00000226763	0.0088	It was shown to be overexpressed in presence of SF3B1 mutations	159
FIG4 ENSG00000112367	0.0086	Not known	
ABCB7 ENSG00000131269	0.0075	Mutant SF3B1 was shown to induce missplicing in ABCB7, resulting in reduced expression	160, 161
BX649632.1 ENSG00000273249	0.0070	Not known	
RNPEPL1 ENSG00000142327	0.0069	Was shown to be differentially spliced in presence of SF3B1 mutations	162, 163
ZNF576 ENSG00000124444	0.0056	Not known	
AL158196.1 ENSG00000276968	0.0054	Not known	
CARD16 ENSG00000204397	0.0051	Not known	
FAM53B-AS1 ENSG00000233334	0.0048	Not known	
LINC01637 ENSG00000237476	0.0047	Not known	
LMAN1 ENSG00000074695	0.0045	Not known	
BTBD6 ENSG00000184887	0.0045	Not known	

Table S55. List of top genes in classification of SPOP alterations, their importance scores, and retrieved associations with SPOP

Gene	Score	Known Associations with SPOP	Ref
CCNG1 ENSG00000113328	0.0176	Not known	
ATP1B3 ENSG00000069849	0.0116	Not known	
ABHD11 ENSG00000106077	0.0078	Not known	
DUSP15 ENSG00000149599	0.0070	Not known	
RNF40 ENSG00000103549	0.0058	Not known, but both RNF40 and SPOP are E3 ubiquitin ligases	
LRRN2 ENSG00000170382	0.0056	Not known	
CALM3 ENSG00000160014	0.0054	Not known	
PTCD1 ENSG00000106246	0.0052	Not known	
TSACC ENSG00000163467	0.0046	Not known	

SORCS2 ENSG00000184985	0.0043	Not known	
FAM186B ENSG00000135436	0.0041	Not known	
AC012508.2 ENSG00000260634	0.0040	Not known	
DDX19A-DT ENSG00000261777	0.0040	Not known	
MRPS33 ENSG00000090263	0.0039	Not known	
TPK1 ENSG00000196511	0.0039	Not known	

Table S56. List of top genes in classification of STAG2 alterations, their importance scores, and retrieved associations with STAG2

Gene	Score	Known Associations with STAG2	Ref
PSMB3P1 ENSG00000258907	0.0076	Not known	
PLCD1 ENSG00000187091	0.0056	Not known	
RGS3 ENSG00000138835	0.0043	Not known	
NINJ1 ENSG00000131669	0.0042	Not known	
ACAD9 ENSG00000177646	0.0042	Not known	
ATP2A1 ENSG00000196296	0.0034	Not known	
KCNMB4 ENSG00000135643	0.0034	Not known	
TRAIP ENSG00000183763	0.0034	Not known	
AC005586.2 ENSG00000261305	0.0032	Not known	
AC002401.3 ENSG00000275025	0.0032	Not known	
SNHG8 ENSG00000269893	0.0032	Not known	
ACAA1 ENSG00000060971	0.0031	Not known	
MRPS25 ENSG00000131368	0.0029	Not known	

Table S57. List of top genes in classification of APC alterations when all tumour types were included in the analysis, their importance scores, and retrieved associations with either APC or colorectal cancers (CRCs)

Gene	Score	Known Associations with APC or COADREAD	Ref
CDX2 ENSG00000165556	0.0381	Intestinal specific transcription factor that activates APC	164
NOX1 ENSG00000007952	0.0325	Is over-expressed in colon cancers and correlates with activating mutations in K-Ras	165
TRABD2A ENSG00000186854	0.0319	Antagonizes Wnt function	166
RNF43 ENSG00000108375	0.0279	Its mutations are associated with aggressive CRCs	167
AL117382.2 ENSG00000226812	0.0263	Not known	
AP003774.2 ENSG00000236935	0.0241	Not known	
LY6G6D ENSG00000244355	0.0236	Significantly overexpressed in CRC when compared with other human solid tumours	28
GPA33 ENSG00000143167	0.0227	An antigen expressed in >95% of colon cancers	168
CDX1 ENSG00000113722	0.0224	Necessary for the proper development of the intestinal tract and homeostasis of the intestinal epithelium. Also plays a role in CRC aggressiveness	169
AXIN2 ENSG00000168646	0.0217	Plays a role in the pathogenesis of colorectal cancer	170

Table S58. List of top genes in classification of BRAF alterations when only thyroid tumour samples were included in the analysis, their importance scores, and retrieved associations with BRAF

Gene	Score	Known Associations with BRAF	Ref
DCSTAMP ENSG00000164935	0.0487	Is overexpressed in presence of BRAF mutations in thyroid cancer	50,51
TMPRSS6 ENSG00000187045	0.0436	Is overexpressed in presence of BRAF mutations in thyroid cancer	52
PNPLA5 ENSG00000100341	0.0306	Not known	
ERBB3 ENSG00000065361	0.0305	Is one of the most potent activators of AKT pathway and a key factor in development of resistance to BRAF and MEK inhibitors	54
NECTIN4 ENSG00000143217	0.0304	Is overexpressed in presence of BRAF mutations in melanoma	53
PDLIM4 ENSG00000131435	0.0259	Is overexpressed in presence of BRAF mutations in thyroid cancer	56
FN1 ENSG00000115414	0.0205	Is differentially expressed between BRAF-wt and BRAF-mut samples in thyroid cancer	59
CRLF2 ENSG00000205755	0.0184	Not known	
SYT12 ENSG00000173227	0.0164	It is associated with BRAF mutated thyroid tumours	60

LY6G6C ENSG00000204421	0.0162	Not known	
KLK7 ENSG00000169035	0.0152	Is overexpressed in presence of BRAF mutations in thyroid cancer	57
SLC34A2 ENSG00000157765	0.0149	Is overexpressed in BRAF mutant and underexpressed in BRAF wild-type thyroid tumours	51,61
EPHA10 ENSG00000183317	0.0138	Is significantly downregulated in thyroid tumours with wild-type BRAF	51
KLK10 ENSG00000129451	0.0121	Is hypomethylated in thyroid tumours with mutated BRAF	55
TACSTD2 ENSG00000184292	0.0115	Is overexpressed in presence of BRAF mutations in thyroid cancer	58

Table S59. List of top genes in classification of BRAF alterations when only colorectal tumour samples were included in the analysis, their importance scores, and retrieved associations with BRAF

Gene	Score	Known Associations with BRAF	Ref
CTTNBP2 ENSG00000077063	0.0165	Was shown to be a gene fusion partner with BRAF	171, 172
RPS4XP7 ENSG00000218265	0.0153	Not known	
PTPRD-AS1 ENSG00000225706	0.0131	Not known	
AP003774.2 ENSG00000236935	0.0112	Not known	
LY6G6F-LY6G6D ENSG00000250641	0.0110	Not known	
TRIM7 ENSG00000146054	0.0109	Is phosphorylated and activated in a BRAF dependent manner	173
LY6G6D ENSG00000244355	0.0109	Is highly downregulated in colorectal cancer with mutated BRAF	174
TDGF1 ENSG00000241186	0.0102	Not known	
ADGB ENSG00000118492	0.0095	Not known	
SLC30A2 ENSG00000158014	0.0094	Was found to be downregulated in BRAF wild-type thyroid tumours	51
CELP ENSG00000170827	0.0093	Not known	
ADGRG6 ENSG00000112414	0.0082	Not known	
TFAP2A ENSG00000137203	0.0081	Not known	
TRPV6 ENSG00000165125	0.0078	Not known	
POU5F1B ENSG00000212993	0.0075	Not known	

Table S60. Top five pathways affected by gene alterations with p-value greater than 0.05 (Fewer pathways reported if less than five meet this threshold).

Gene	Category	Pathway	P-value	Adjusted P-value
APC	GOTERM_BP_DIRECT	Stem Cell Proliferation	0.0013	0.20
	UP_SEQ_FEATURE	LIPID: GPI-anchor Amidated Serine	0.0015	0.25
	INTERPRO	ZNRF-3 Ecto	0.0020	0.11
	GOTERM_BP_DIRECT	Wnt Receptor Catabolic Process	0.0022	0.20
	INTERPRO	LY6G6d/LY6G6f	0.0031	0.11
ARID1A	UP_KW_MOLECULAR_FUNCTION	Ion Channel	3.6e-4	0.01
	UP_KW_MOLECULAR_FUNCTION	Voltage-gated Channel	0.0021	0.03
	GOTERM_MF_DIRECT	Transmembrane Transporter Binding	0.0026	0.20
	GOTERM_BP_DIRECT	Action Potential	0.0031	0.47
	UP_KW_BIOLOGICAL_PROCESS	Ion Transport	0.0035	0.067
ATR	GOTERM_BP_DIRECT	Cell Division	2.2e-15	5.7e-13
	UP_KW_BIOLOGICAL_PROCESS	Cell Division	5.1e-15	1.6e-13
	UP_KW_BIOLOGICAL_PROCESS	Mitosis	4.1e-14	6.6e-13
	UP_KW_BIOLOGICAL_PROCESS	Cell Cycle	3.7e-13	3.9e-12
	GOTERM_BP_DIRECT	Mitotic Cell Cycle	1.1e-10	1.4e-8
ATRX	GOTERM_BP_DIRECT	Cytoplasmic Translation	0.0011	0.34
	GOTERM_BP_DIRECT	Positive Regulation of Protein Binding	0.0043	0.69
	GOTERM_CC_DIRECT	Cytosolic Large Ribosomal Subunit	0.0093	0.65
	GOTERM_CC_DIRECT	Cytosolic Ribosome	0.013	0.65
	GOTERM_BP_DIRECT	Cellular Response to Peptidoglycan	0.014	1.0
BRAF	GOTERM_CC_DIRECT	Extracellular Region	1.8e-6	2.3e-4
	UP_KW_CELLULAR_COMPONENT	Secreted	2.7e-5	6.4e-4
	UP_SEQ_FEATURE	DOMAIN: UPAR/Ly6	2.3e-4	0.085
	GOTERM_CC_DIRECT	Plasma Membrane	2.6e-4	0.017
	INTERPRO	PH Domain	3.2e-4	0.047
BRCA1	GOTERM_BP_DIRECT	Negative Regulation of Myofibroblast Differentiation	0.0039	0.34
	GOTERM_BP_DIRECT	Response to Alkaloid	0.0061	0.34
	UP_KW_DISEASE	Williams-Beuren Syndrome	0.012	0.025
	GOTERM_BP_DIRECT	Response to Hormone	0.021	0.78
	GOTERM_CC_DIRECT	Dendrite	0.023	0.68
CDH1	UP_KW_MOLECULAR_FUNCTION	RNA-binding	0.035	0.39
	GOTERM_CC_DIRECT	Cytoplasmic Stress Granule	0.046	1.0

	GOTERM_BP_DIRECT	Negative Regulation of Translation	0.050	1.0
CDKN2A	UP_KW_BIOLOGICAL_PROCESS	Mitosis	3.5e-32	1.2e-30
	UP_KW_BIOLOGICAL_PROCESS	Cell Cycle	5.1e-28	6.8e-27
	UP_KW_BIOLOGICAL_PROCESS	Cell Division	6.2e-28	6.8e-27
	GOTERM_BP_DIRECT	Cell Division	6.9e-27	2.6e-24
	UP_KW_CELLULAR_COMPONENT	Centromere	1.2e-14	2.4e-13
CTCF	UP_KW_BIOLOGICAL_PROCESS	Cell Division	7.1e-7	9.8e-6
	UP_KW_BIOLOGICAL_PROCESS	Cell Cycle	1.4e-6	9.8e-6
	UP_KW_BIOLOGICAL_PROCESS	Mitosis	1.7e-6	9.8e-6
	GOTERM_BP_DIRECT	Cell Division	1.9e-6	3.9e-4
	GOTERM_MF_DIRECT	Protein Kinase Binding	1.4e-5	1.2e-3
CTNNB1	UP_KW_CELLULAR_COMPONENT	Endoplasmic Reticulum	5.6e-4	0.0067
	GOTERM_CC_DIRECT	Endoplasmic Reticulum Membrane	0.0019	0.095
	GOTERM_BP_DIRECT	Apoptotic Process	0.0094	0.72
	UP_SEQ_FEATURE	CARBOHYD: N-linked (GlcNAc…) Asparagine	0.011	0.65
	UP_SEQ_FEATURE	TRANSMEM: Helical	0.011	0.65
EGFR	UP_KW_CELLULAR_COMPONENT	Golgi Apparatus	0.0076	0.15
	GOTERM_BP_DIRECT	Stimulatory C-type Lectin Receptor Signaling Pathway	0.024	1.0
	BIOCARTA	Ras-Independent Pathway in NK Cell-mediated Cytotoxicity	0.025	0.049
	GOTERM_BP_DIRECT	Positive Regulation of Natural Killer Cell mediated Cytotoxicity	0.029	1.0
	UP_KW_CELLULAR_COMPONENT	Cytoplasm	0.038	0.38
EZH2	GOTERM_BP_DIRECT	Anatomical Structure Development	0.032	1.0
FBXW7	UP_KW_BIOLOGICAL_PROCESS	Mitosis	8.9e-34	2.2e-32
	UP_KW_BIOLOGICAL_PROCESS	Cell Cycle	8.3e-31	1.0e-29
	UP_KW_BIOLOGICAL_PROCESS	Cell Division	1.1e-29	9.5e-29
	GOTERM_BP_DIRECT	Cell Division	1.2e-27	3.8e-25
	UP_KW_CELLULAR_COMPONENT	Centromere	1.4e-19	2.7e-18
GATA3	GOTERM_BP_DIRECT	Cell Division	8.8e-23	2.3e-20
	UP_KW_BIOLOGICAL_PROCESS	Mitosis	8.0e-22	2.2e-20
	UP_KW_BIOLOGICAL_PROCESS	Cell Division	5.2e-19	7.3e-18
	UP_KW_BIOLOGICAL_PROCESS	Cell Cycle	4.6e-18	4.3e-17
	UP_KW_CELLULAR_COMPONENT	Centromere	9.1e-16	1.2e-14
KDM6A	INTERPRO	G_TR_CS	1.2e-5	6.5e-4
	UP_SEQ_FEATURE	DOMAIN: Tr-type G	2.9e-5	0.0011
	INTERPRO	EFTu-like_2	3.1e-5	8.7e-4
	GOTERM_MF_DIRECT	GTPase Activity	3.4e-5	8.8e-4
	GOTERM_BP_DIRECT	Translational Elongation	3.9e-5	0.0015
KEAP1	GOTERM_CC_DIRECT	Neuronal Cell Body	0.0023	0.14
	GOTERM_CC_DIRECT	Plasma Membrane	0.0028	0.14

	UP_SEQ_FEATURE	DOMAIN: F5/8 Type C2	0.011	1.0
	UP_SEQ_FEATURE	DOMAIN: F5/8 Type C1	0.011	1.0
	GOTERM_BP_DIRECT	Cellular Response to Leukemia Inhibitory Factor	0.012	1.0
KIT	UP_KW_BIOLOGICAL_PROCESS	Mitosis	4.3e-19	1.4e-17
	UP_KW_BIOLOGICAL_PROCESS	Cell Cycle	2.8e-18	4.6e-17
	UP_KW_BIOLOGICAL_PROCESS	Cell Division	5.7e-18	6.0e-17
	GOTERM_BP_DIRECT	Cell Division	7.2e-18	1.9e-15
	GOTERM_CC_DIRECT	Kinetochore	1.9e-13	1.6e-11
KRAS	UP_SEQ_FEATURE	CARBOHYD: N-linked (GlcNAc…) Asparagine	4.6e-4	0.083
	GOTERM_CC_DIRECT	Plasma Membrane	4.8e-4	0.031
	GOTERM_CC_DIRECT	Cell Surface	0.0073	0.24
	UP_SEQ_FEATURE	DOMAIN: Ig-like V-type	0.011	1.0
	UP_KW_PTM	Glycoprotein	0.013	0.14
MAP3K1	UP_KW_BIOLOGICAL_PROCESS	Mitosis	5.5e-16	1.2e-14
	UP_KW_BIOLOGICAL_PROCESS	Cell Division	2.5e-15	2.8e-14
	GOTERM_BP_DIRECT	Cell Division	2.8e-14	5.1e-12
	UP_KW_BIOLOGICAL_PROCESS	Cell Cycle	1.7e-13	1.2e-12
	GOTERM_BP_DIRECT	Mitotic Cell Cycle	2.9e-9	2.7e-7
NCOR1	UP_KW_BIOLOGICAL_PROCESS	Mitosis	1.3e-16	3.2e-15
	UP_KW_BIOLOGICAL_PROCESS	Cell Cycle	9.4e-16	1.2e-14
	UP_KW_BIOLOGICAL_PROCESS	Cell Division	1.9e-14	1.6e-13
	GOTERM_BP_DIRECT	Cell Division	3.4e-11	1.1e-8
	UP_KW_CELLULAR_COMPONENT	Centromere	1.1e-10	2.2e-9
NF1	UP_KW_BIOLOGICAL_PROCESS	Mitosis	6.2e-16	1.5e-14
	UP_KW_BIOLOGICAL_PROCESS	Cell Division	6.3e-14	7.9e-13
	GOTERM_BP_DIRECT	Cell Division	8.1e-14	1.9e-11
	UP_KW_BIOLOGICAL_PROCESS	Cell Cycle	3.5e-11	2.9e-10
	UP_KW_CELLULAR_COMPONENT	Centromere	1.3e-9	2.3e-8
NOTCH1	GOTERM_CC_DIRECT	Uniplex Complex	0.0092	0.60
	GOTERM_BP_DIRECT	Mitochondrial Calcium Ion Transmembrane Transport	0.015	1.0
	GOTERM_BP_DIRECT	Calcium Import into the Mitochondrion	0.017	1.0
	GOTERM_BP_DIRECT	Mitochondrial Calcium Ion Homeostasis	0.021	1.0
	UP_SEQ_FEATURE	TRANSIT: Mitochondrion	0.032	1.0
NRAS	GOTERM_BP_DIRECT	Inflammatory Response	2.2e-4	0.093
	GOTERM_BP_DIRECT	Regulation of Canonical NF-kappaB Signal Transduction	2.3e-4	0.093
	GOTERM_BP_DIRECT	Cell-cell Junction Maintenance	4.0e-4	0.11

	GOTERM_BP_DIRECT	Positive Regulation of Chemokine Production	6.1e-4	0.12
	GOTERM_BP_DIRECT	Signal Transduction	8.4e-4	0.14
NSD1	UP_KW_BIOLOGICAL_PROCESS	Mitosis	3.5e-18	6.0e-17
	UP_KW_BIOLOGICAL_PROCESS	Cell Division	9.3e-18	7.9e-17
	GOTERM_BP_DIRECT	Cell Division	7.5e-16	1.5e-13
	UP_KW_BIOLOGICAL_PROCESS	Cell Cycle	1.6e-14	8.9e-14
	GOTERM_BP_DIRECT	Mitotic Cell Cycle	9.1e-10	9.2e-8
PBRM1	GOTERM_MF_DIRECT	Protein Homodimerization Activity	0.0041	0.20
	GOTERM_BP_DIRECT	Positive Regulation of Protein-containing Complex Assembly	0.023	1.0
PIK3CA	UP_KW_MOLECULAR_FUNCTION	Transferase	0.019	0.24
	UP_SEQ_FEATURE	TRANSMEM: Helical; Signal -anchor for type II Membrane Protein	0.043	1.0
PTEN	UP_KW_BIOLOGICAL_PROCESS	Cell Cycle	6.9e-16	1.3e-14
	UP_KW_BIOLOGICAL_PROCESS	Mitosis	7.4e-16	1.3e-14
	GOTERM_BP_DIRECT	Cell Division	1.1e-14	4.4e-12
	UP_KW_BIOLOGICAL_PROCESS	Cell Division	1.1e-14	1.2e-13
	UP_KW_CELLULAR_COMPONENT	Centromere	2.4e-9	4.9e-8
RB1	UP_KW_BIOLOGICAL_PROCESS	Cell Cycle	2.0e-28	2.7e-27
	UP_KW_BIOLOGICAL_PROCESS	Mitosis	2.2e-28	2.7e-27
	GOTERM_BP_DIRECT	Cell Division	1.6e-26	6.1e-24
	UP_KW_BIOLOGICAL_PROCESS	Cell Division	1.4e-24	1.1e-23
	UP_KW_CELLULAR_COMPONENT	Centromere	5.2e-17	9.9e-16
SETBP1	No pathway was found based on the top eight genes in classification			
SETD2	UP_KW_BIOLOGICAL_PROCESS	Transport	0.0033	0.02
	GOTERM_MF_DIRECT	Monoatomic Cation Transmembrane Transporter Activity	0.0052	0.14
	GOTERM_MF_DIRECT	Efflux Transmembrane Transporter Activity	0.0072	0.14
SF3B1	GOTERM_MF_DIRECT	Cysteine-type Endopeptidase Activator Activity involved in Apoptotic Process	3.7e-4	0.028
	UP_KW_MOLECULAR_FUNCTION	Hydrolase	0.0014	0.022
	GOTERM_CC_DIRECT	Protease Inhibitor Complex	0.0023	0.11
	UP_KW_MOLECULAR_FUNCTION	Protease	0.0024	0.022
	UP_SEQ_FEATURE	MUTAGEN: IKK->AKA: Abolished Ability to Cleave IL18	0.0025	0.041
SPOP	UP_KW_CELLULAR_COMPONENT	Mitochondrion	4.8e-4	0.0086
	GOTERM_BP_DIRECT	Mitochondrial Translation	0.0053	0.67

	GOTERM_CC_DIRECT	Mitochondrion	0.0057	0.31
	GOTERM_BP_DIRECT	Translation	0.021	1.0
	UP_KW_DOMAIN	Transit Peptide	0.023	0.23
STAG2	SMART	C2	0.046	0.46

Table S61. Top five pathways affected by BRAF gene alterations in thyroid and colorectal tumours with p-value greater than 0.05

Tumour Type	Category	Pathway	P-value	Adjusted P-value
Thyroid	UP_KW_CELLULAR_COMPONENT	Secreted	9.0e-7	2.2e-5
	INTERPRO	PH Domain	2.6e-5	0.0072
	UP_SEQ_FEATURE	DOMAIN: PH	3.0e-5	0.013
	GOTERM_CC_DIRECT	Extracellular Region	6.2e-5	0.0084
	UP_SEQ_FEATURE	CARBOHYD: N-linked (GlcNAc…) Asparagine	7.6e-5	0.017
Colorectal	GOTERM_CC_DIRECT	Apical Plasma Membrane	0.0012	0.11
	UP_SEQ_FEATURE	MUTAGEN: Missing: Abolishes MARCHF2-mediated degradation	0.0040	1.0
	INTERPRO	LY6G6d/LY6G6f	0.0060	1.0
	KEGG_PATHWAY	Pancreatic Secretion	0.0070	0.78
	GOTERM_MF_DIRECT	Acetylcholine Receptor Inhibitor Activity	0.017	1.0

Table S62. Non-impactful mutation categories in which most samples were predicted as mutant (the p-value for each mutation type was obtained via a binomial test and it represents the probability of having the specific number of samples assigned to the mutant group assuming there is a 50% chance that samples will be assigned to this group by random assignment).

Gene	Mutation Type	Number of Samples Classified as Mutant	Number of Samples Classified as WT	P-value
EGFR	Intron Variant	21	1	1.1e-5
EZH2	Intron Variant	14	5	0.06
NCOR1	Intron Variant	65	21	2.0e-6
RB1	Intron Variant	92	15	0.0

Supplementary Figures

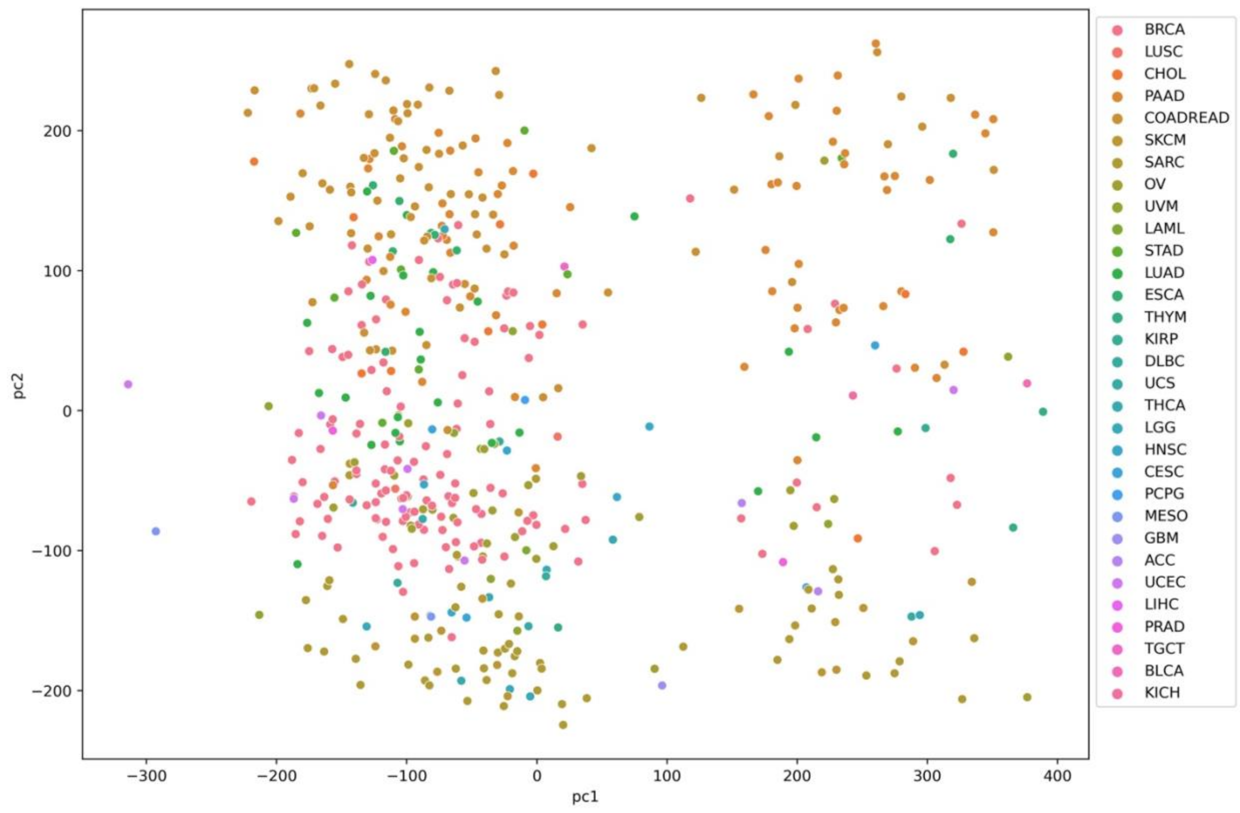

Figure S1. PCA plot made using log2(TPM+0.001) values of all POG samples. Colors represent different tumour types.

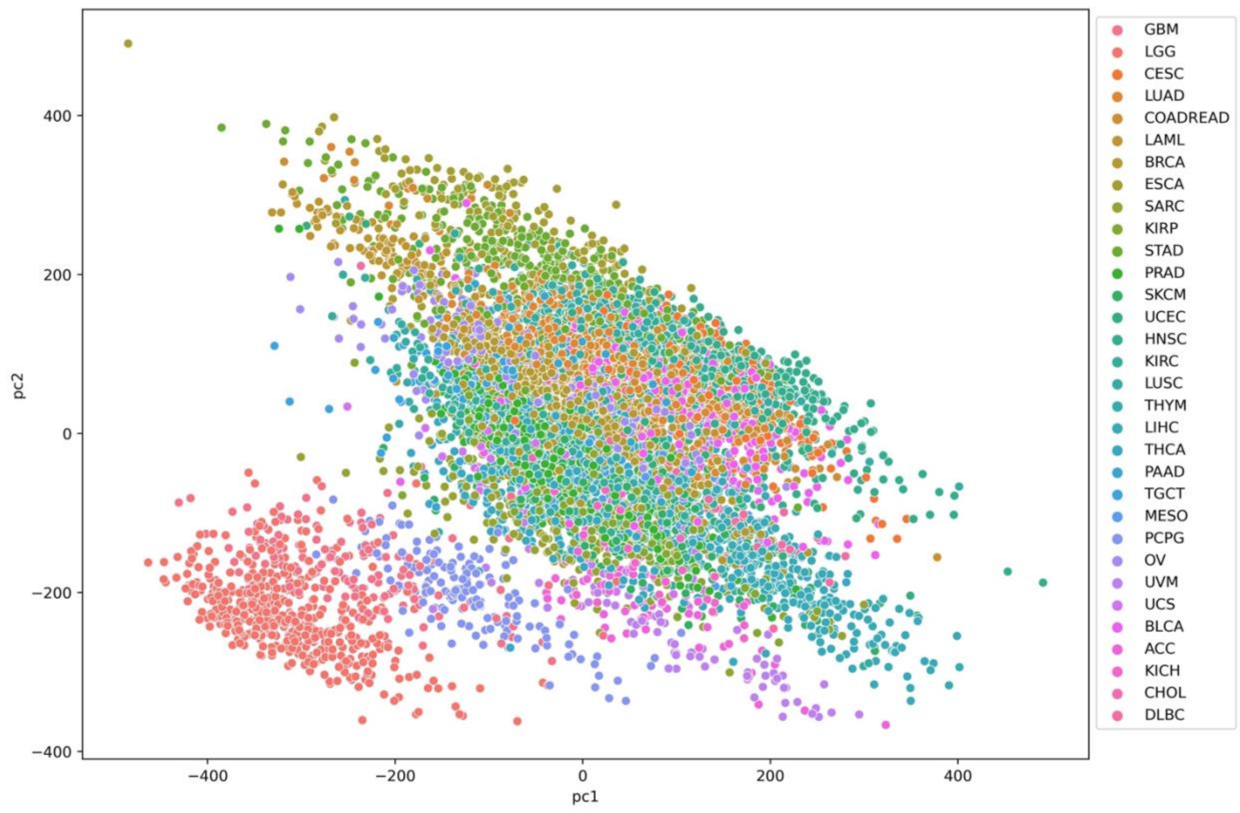

Figure S2. PCA plot made using log2(TPM+0.001) values of all TCGA samples. Colors represent different tumour types.

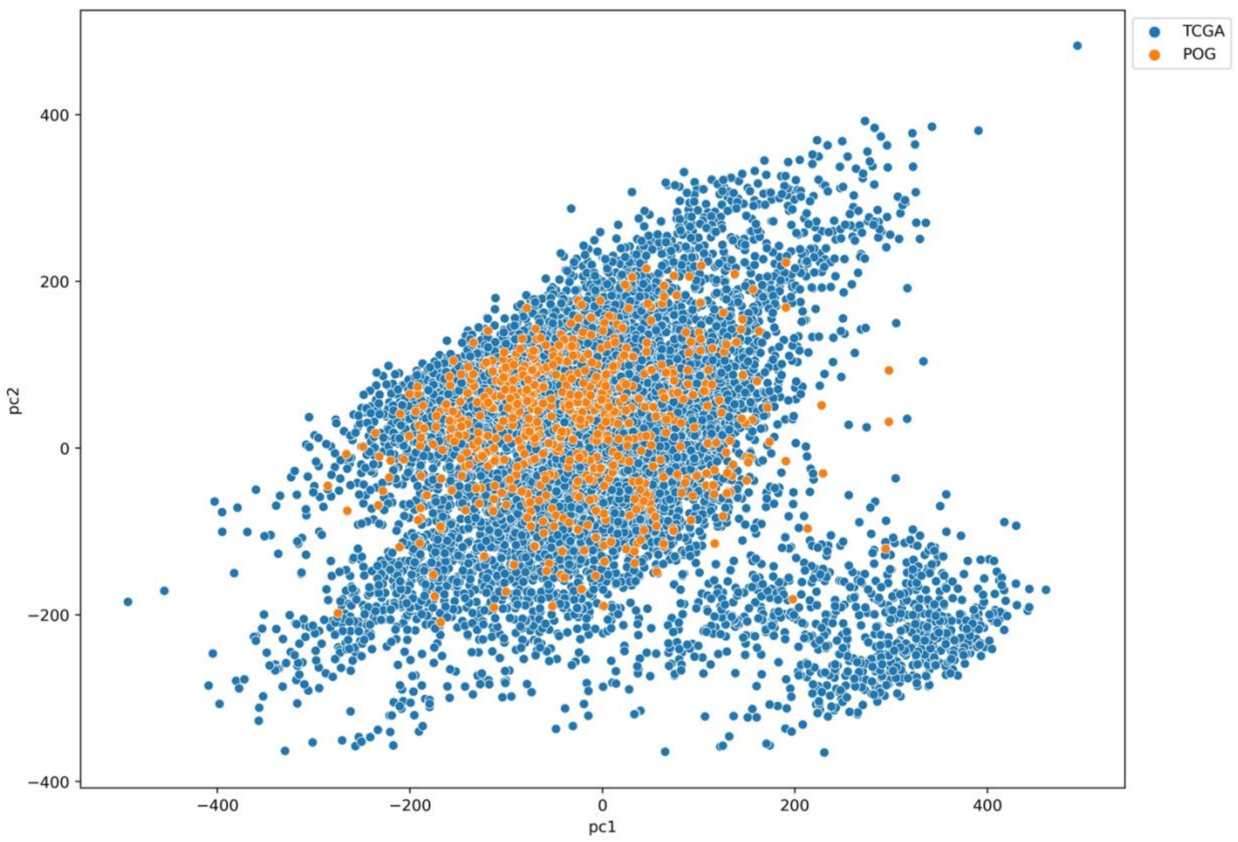

Figure S3. PCA plot made using log2(TPM+0.001) values of all TCGA and POG samples. Colors represent the cohorts that samples are from.

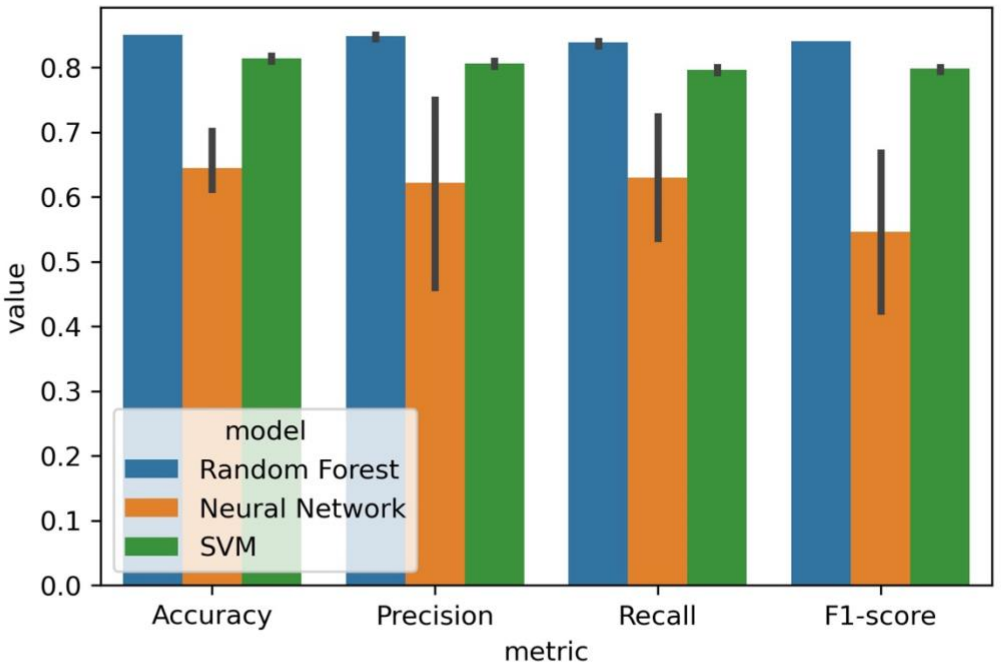

Figure S4. Performance metrics for classification of tumour samples based on *TP53* mutational status using random forest, support vector machine and neural network models.

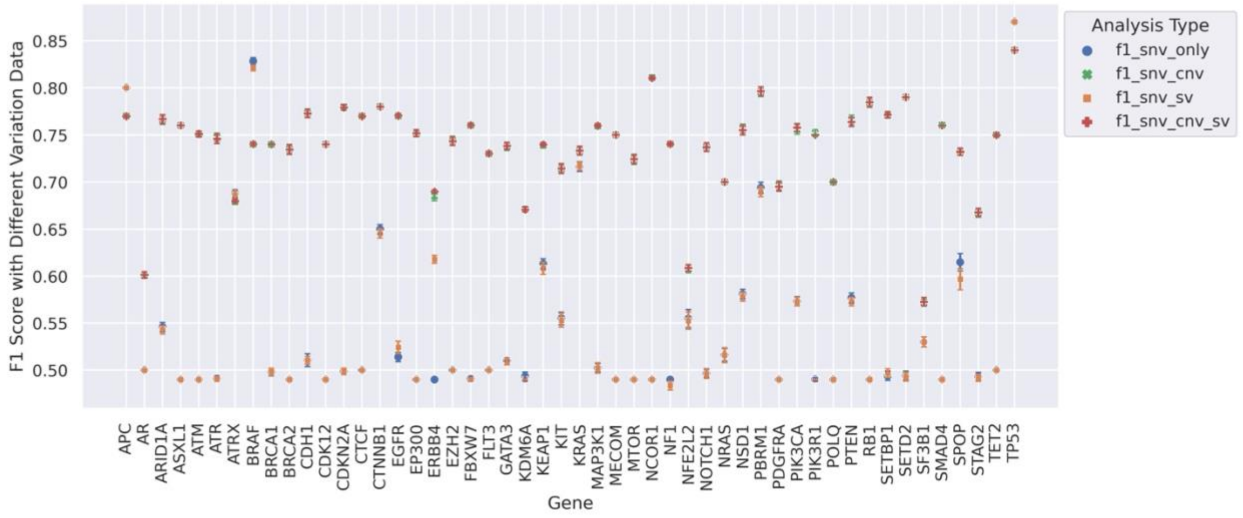

Figure S5. F1 score of classification based on different combinations of gene alterations. Blue circles represent results when only samples with SNVs/INDELs were labeled as mutant. Green crosses indicate results from analyses labeling samples with either SNVs/INDELs or CNAs as mutant. Orange squares show the results when samples with either SNVs/INDELs or SVs were labeled as mutant, and red crosses correspond to analyses where samples with any of SNVs/INDELs, CNAs, or SVs were labeled as mutant.

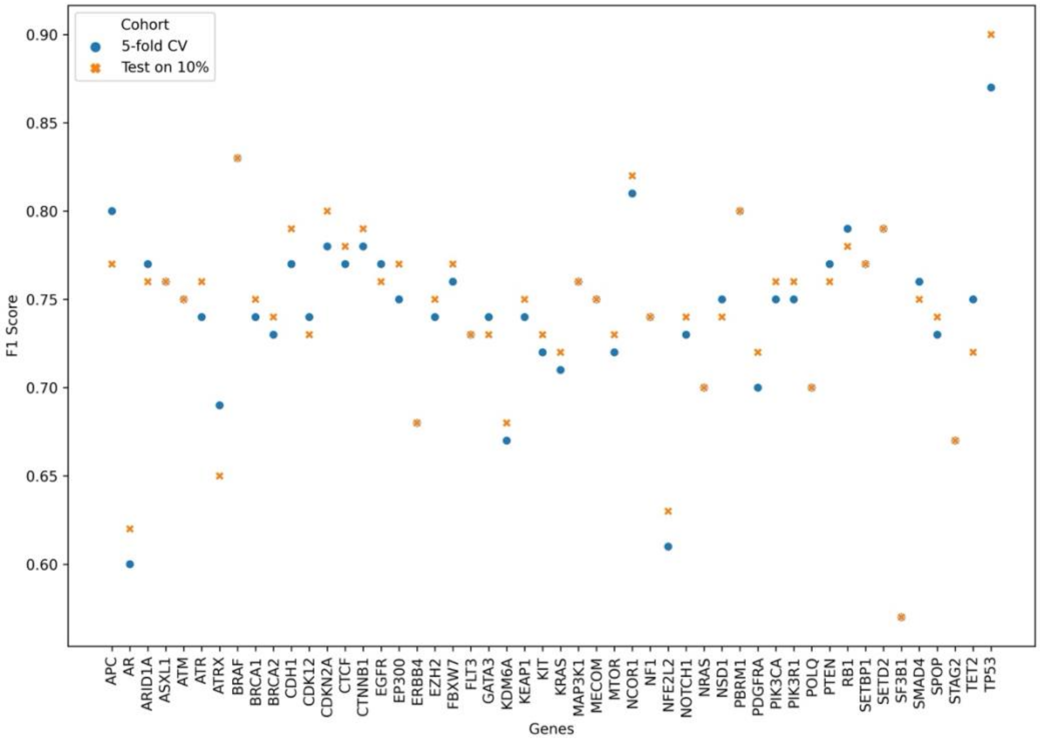

Figure S6. Comparison of F1 scores between 5-fold CV analyses and test on the 10% of randomly selected samples. Blue circles show the F1 scores when all samples were used in 5-fold CV analyses and orange crosses indicate the F1 scores when 90% of samples were used for training and 10% were used for testing.

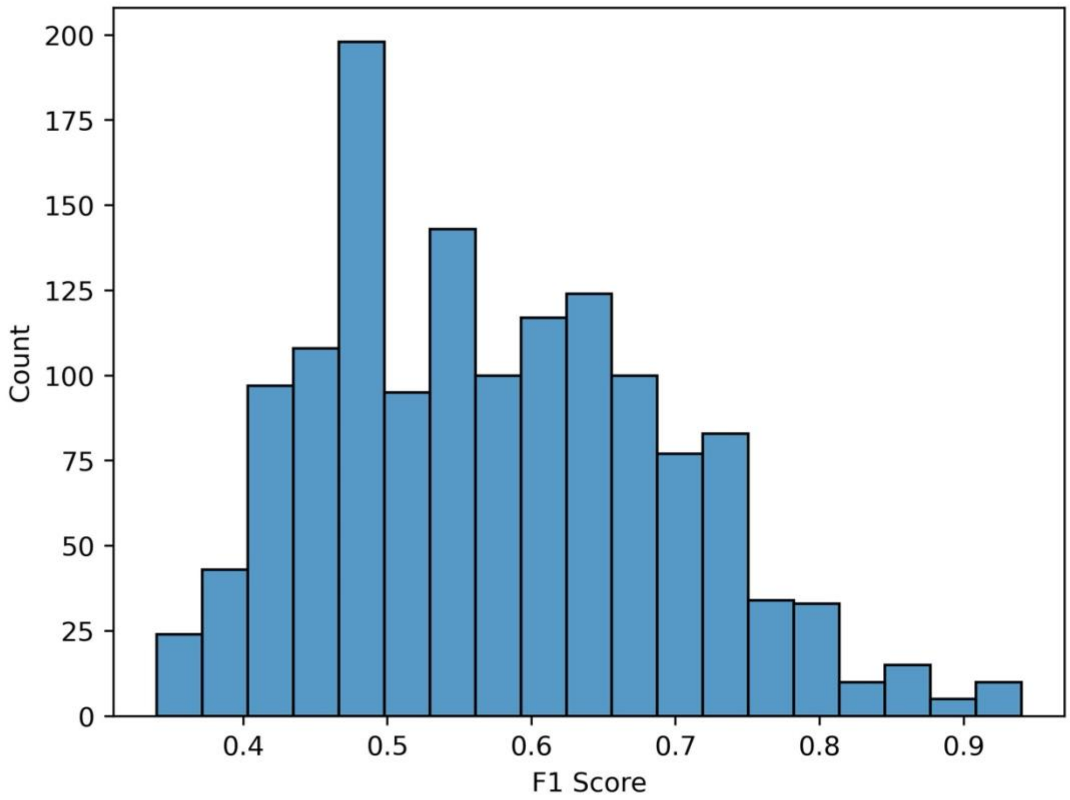

Figure S7. Distribution of F1 scores obtained from classifying samples based on alterations in 50 genes of interest across 33 TCGA tumour types.

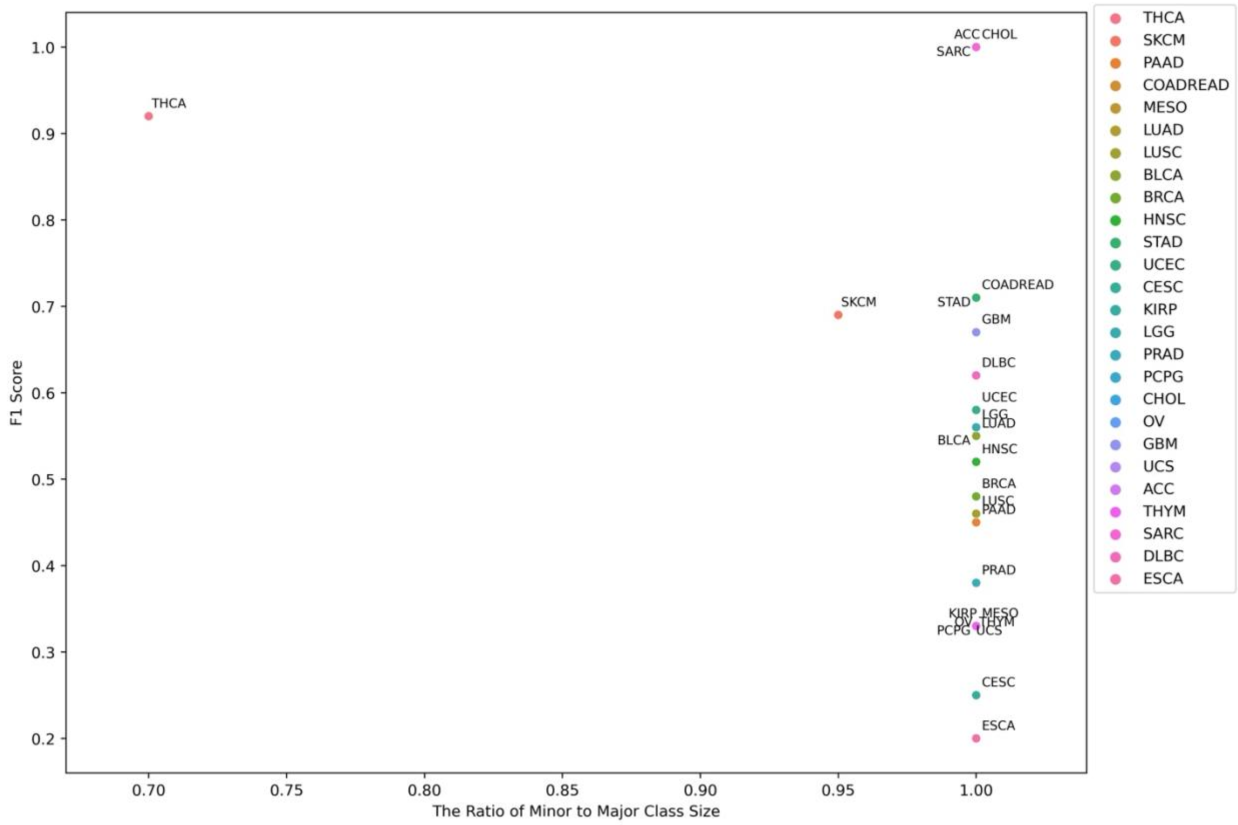

Figure S8. F1 score for classification of all samples in THCA and SKCM tumours and balanced sets of all other tumour types based on BRAF alterations (COAD and READ are combined). X-axis shows the ratio of the minor to the major group. The minor group is the set with either mutant or wild-type samples which is smaller in size and the major group is the set with either mutant or wild-type samples which is larger in size.

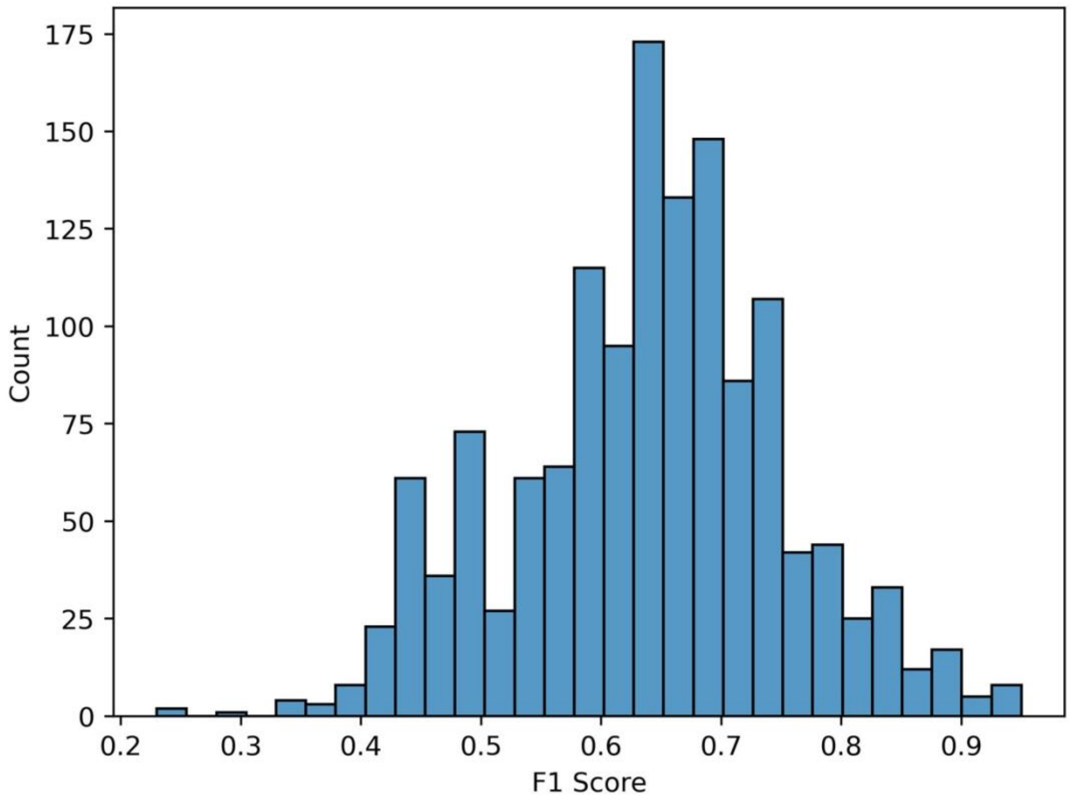

Figure S9. Distribution of F1 scores obtained from classifying balanced sets of samples based on alterations in 50 genes of interest across 33 TCGA tumour types.

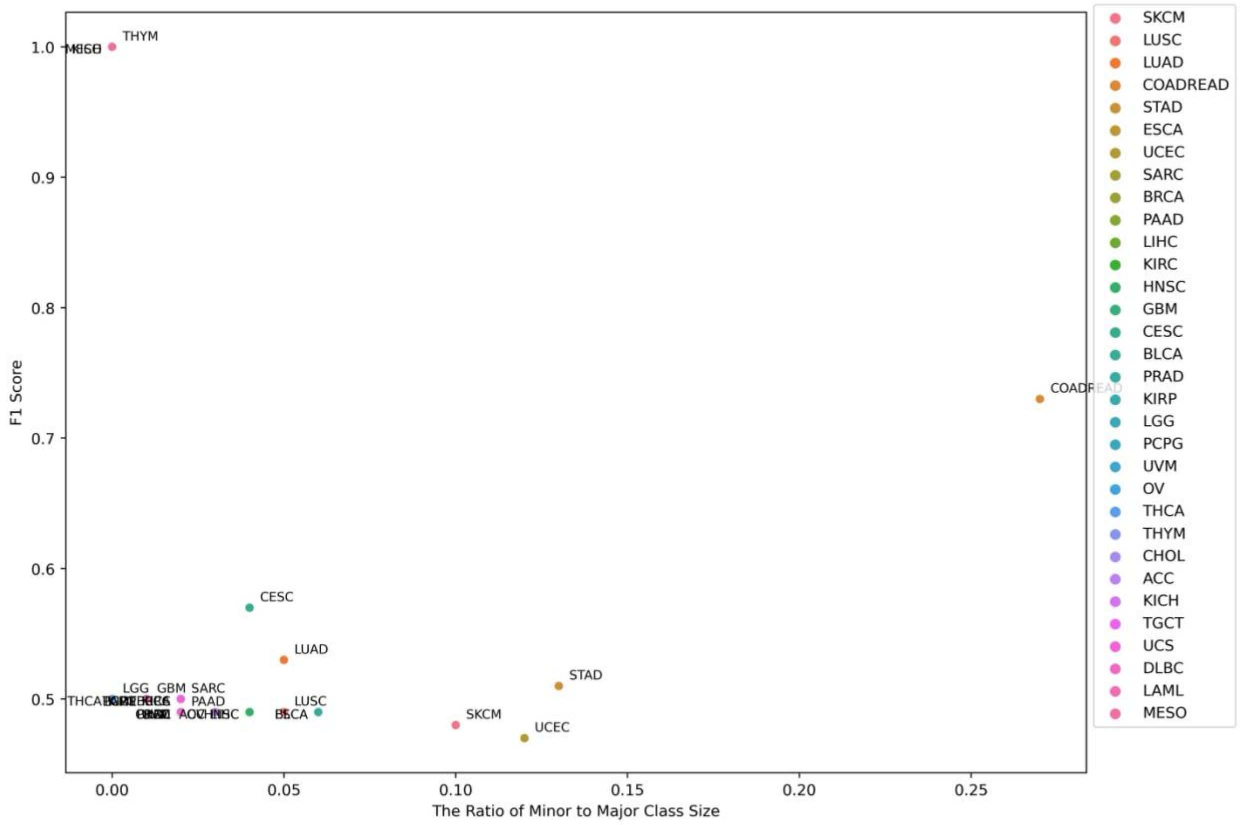

Figure S10. F1 score for classification of samples based on APC alterations against the ratio of the minor to the major group across 33 TCGA tumour type (COAD and READ are combined). The minor group is the set with either mutant or wild-type samples which is smaller in size and the major group is the set with either mutant or wild-type samples which is larger in size

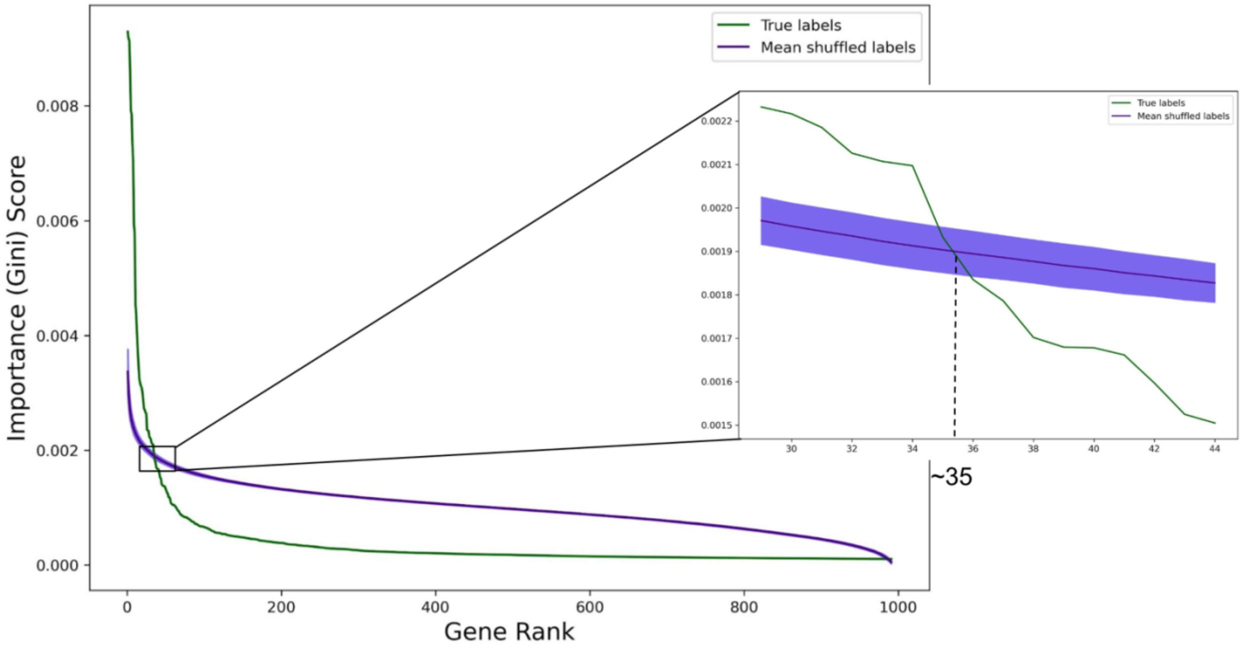

Figure S11. The importance scores in classification of KRAS alterations versus gene ranks extracted from the model trained using true labels (green) and the mean and standard deviation of importance scores of the gene ranks over 100 permutations of training the random forest with randomly shuffled labels (purple).

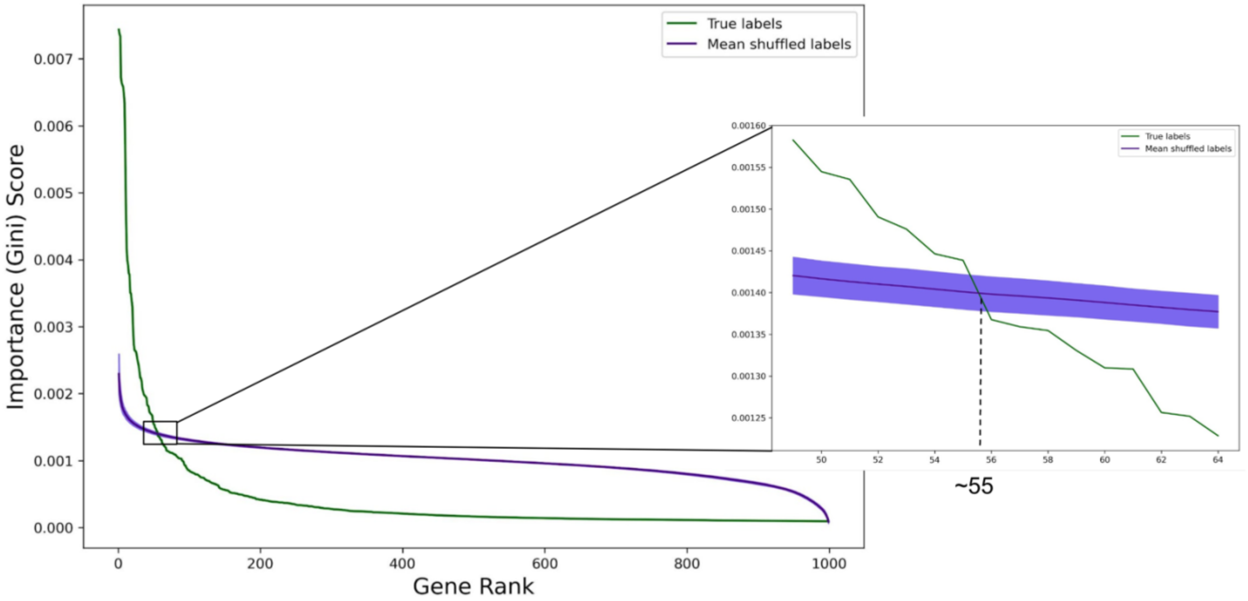

Figure S12. The importance scores in classification of PTEN alterations versus gene ranks extracted from the model trained using true labels (green) and the mean and standard deviation

of importance scores of the gene ranks over 100 permutations of training the random forest with randomly shuffled labels (purple).

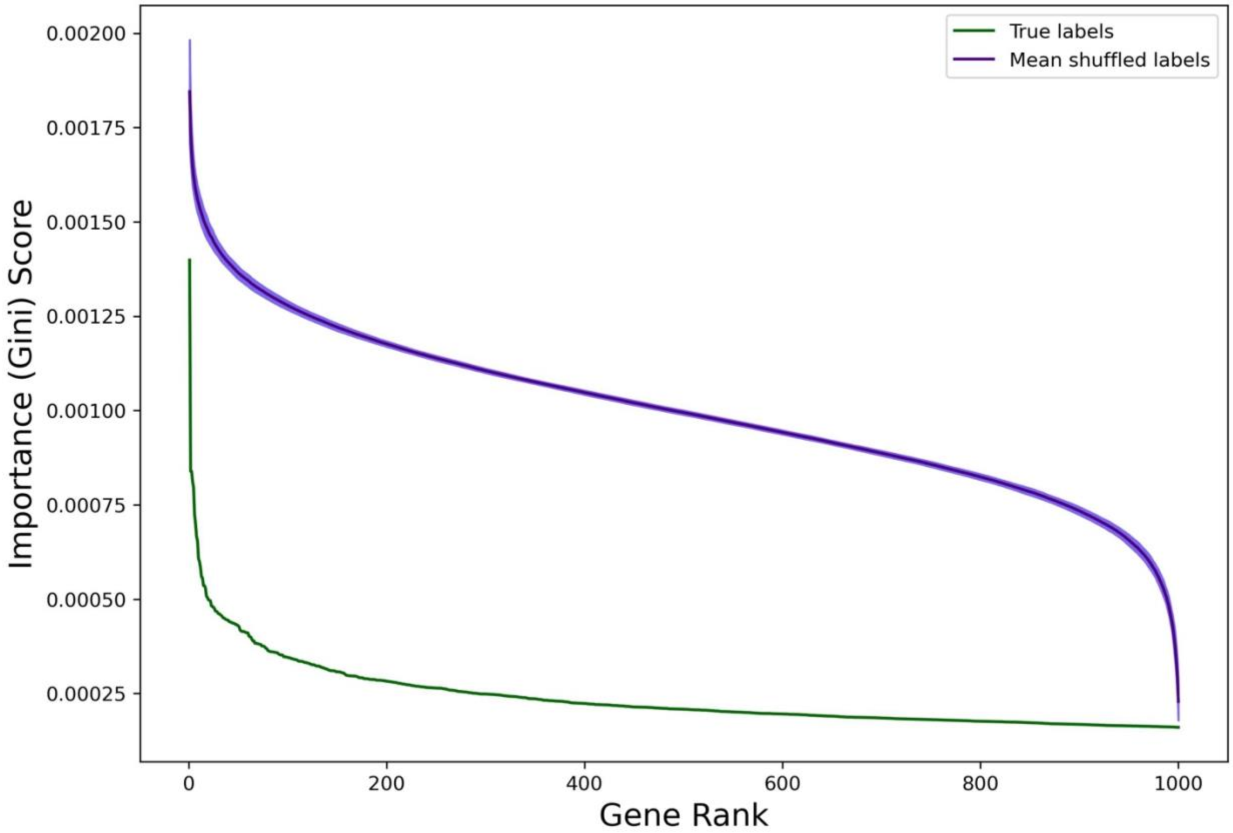

Figure S13. The importance scores in classification of AR alterations versus gene ranks extracted from the model trained using true labels (green) and the mean and standard deviation of importance scores of the gene ranks over 100 permutations of training the random forest with randomly shuffled labels (purple).

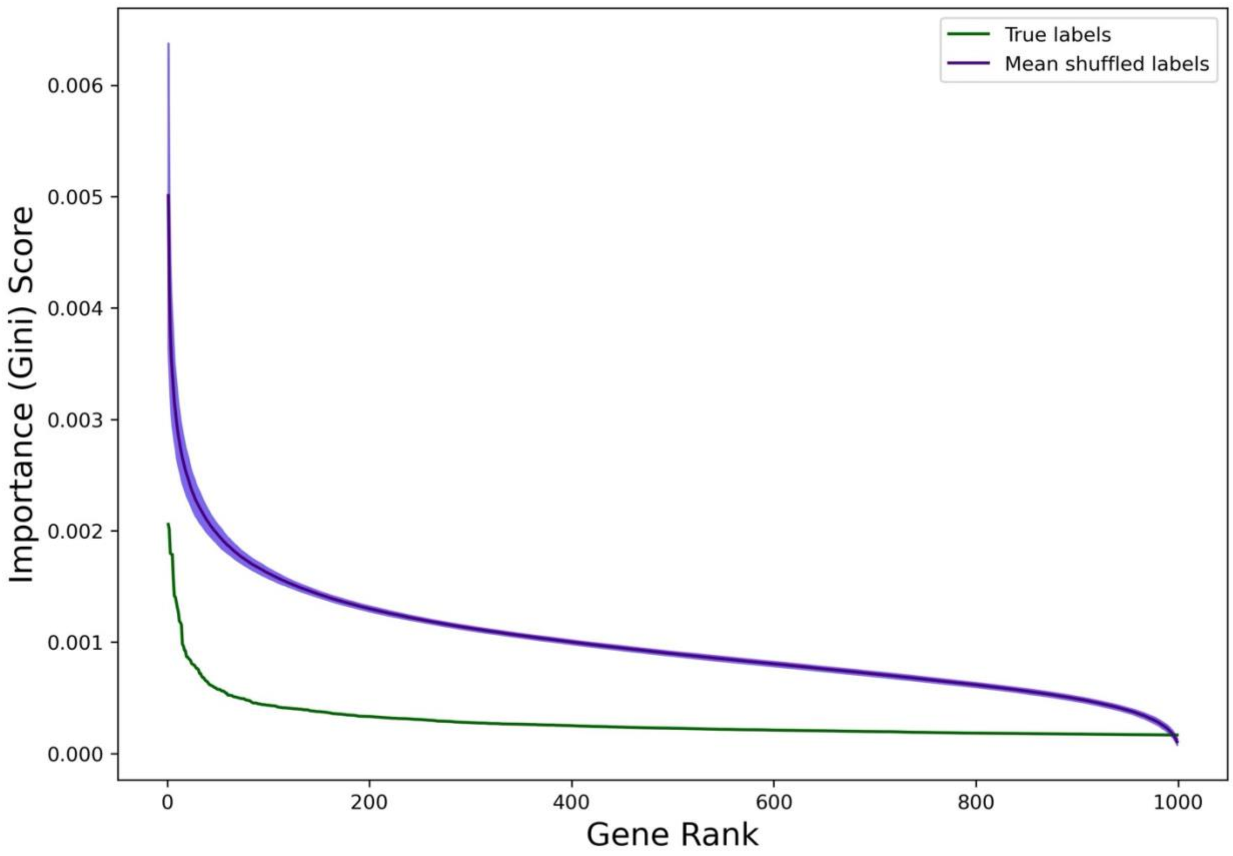

Figure S14. The importance scores in classification of ERBB4 alterations versus gene ranks extracted from the model trained using true labels (green) and the mean and standard deviation of importance scores of the gene ranks over 100 permutations of training the random forest with randomly shuffled labels (purple).

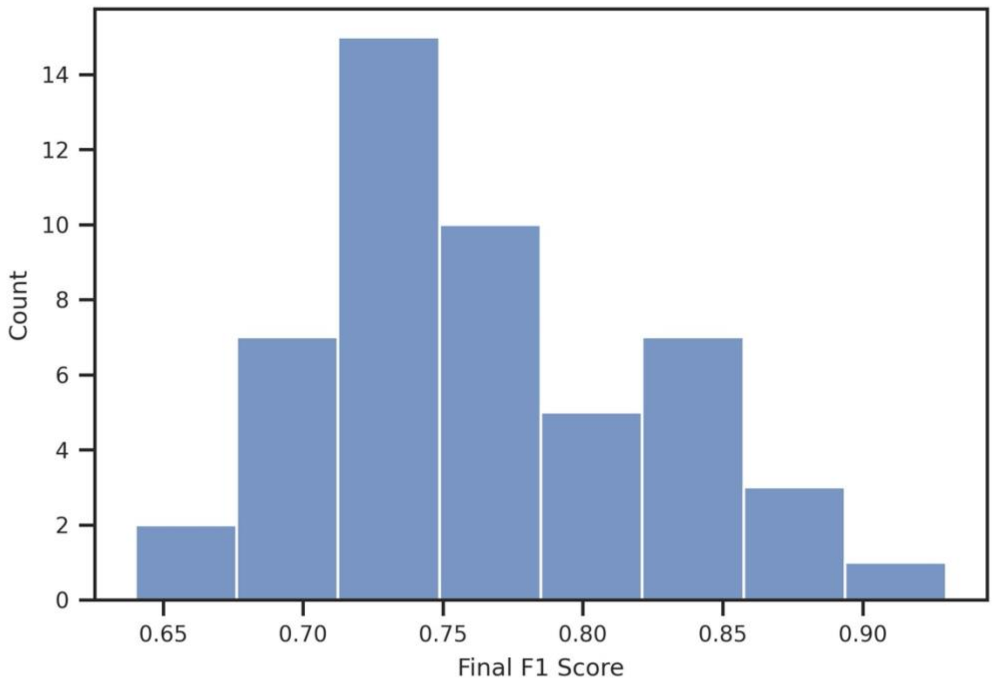

Figure S15. Distribution of F1 scores obtained from performing 5-fold CV on samples selected based on the best mode of analysis found previously.

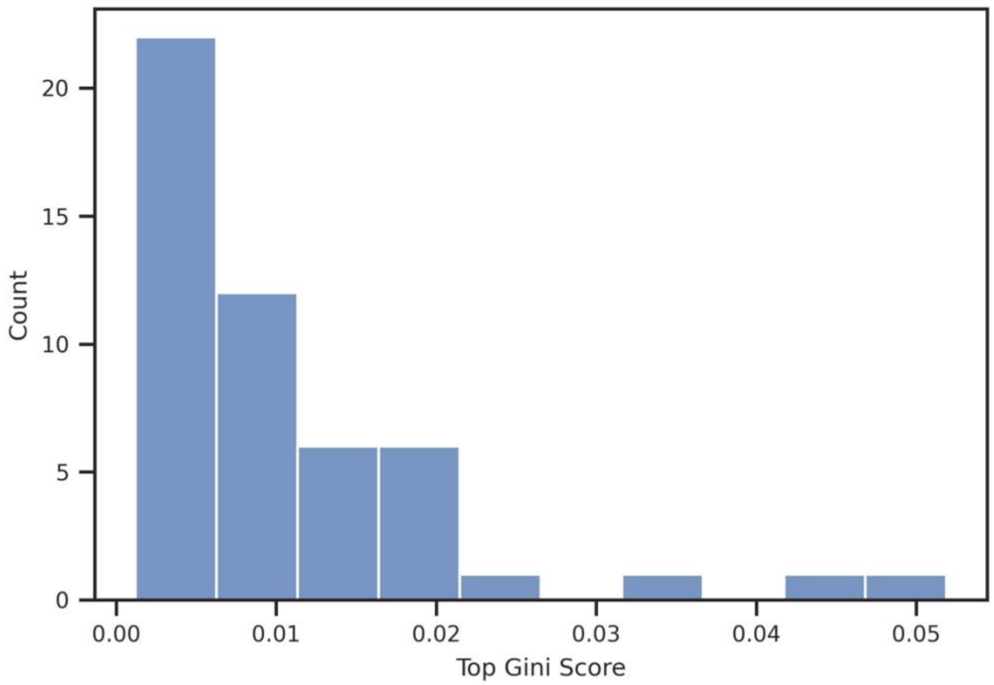

Figure S16. Distribution of top Gini scores from the most contributing feature to classification obtained after training the model using all samples from the best mode of analysis found previously.

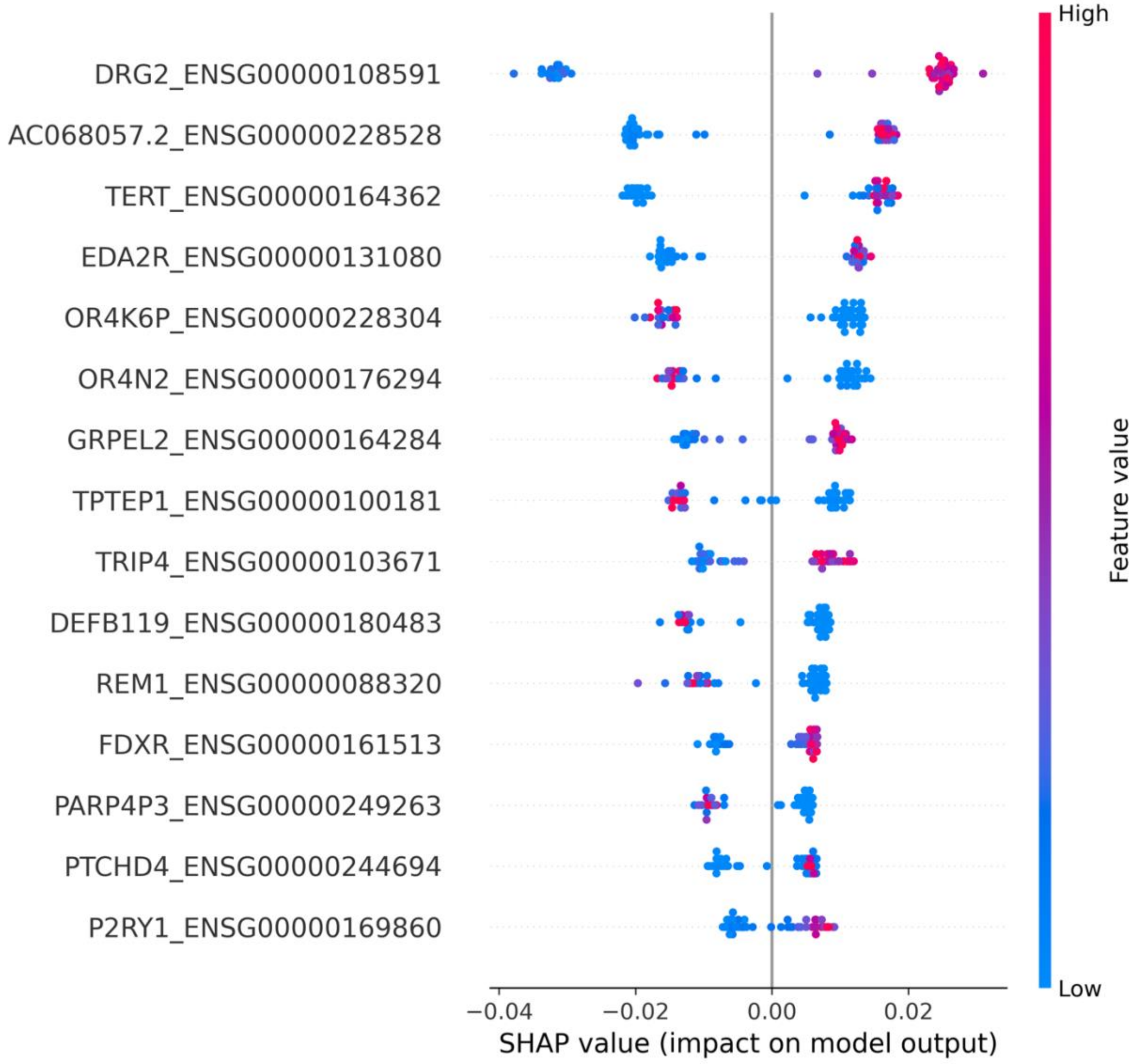

Figure S17. SHAP importance of top 15 features contributing to classification of samples based on *ATRX* mutations

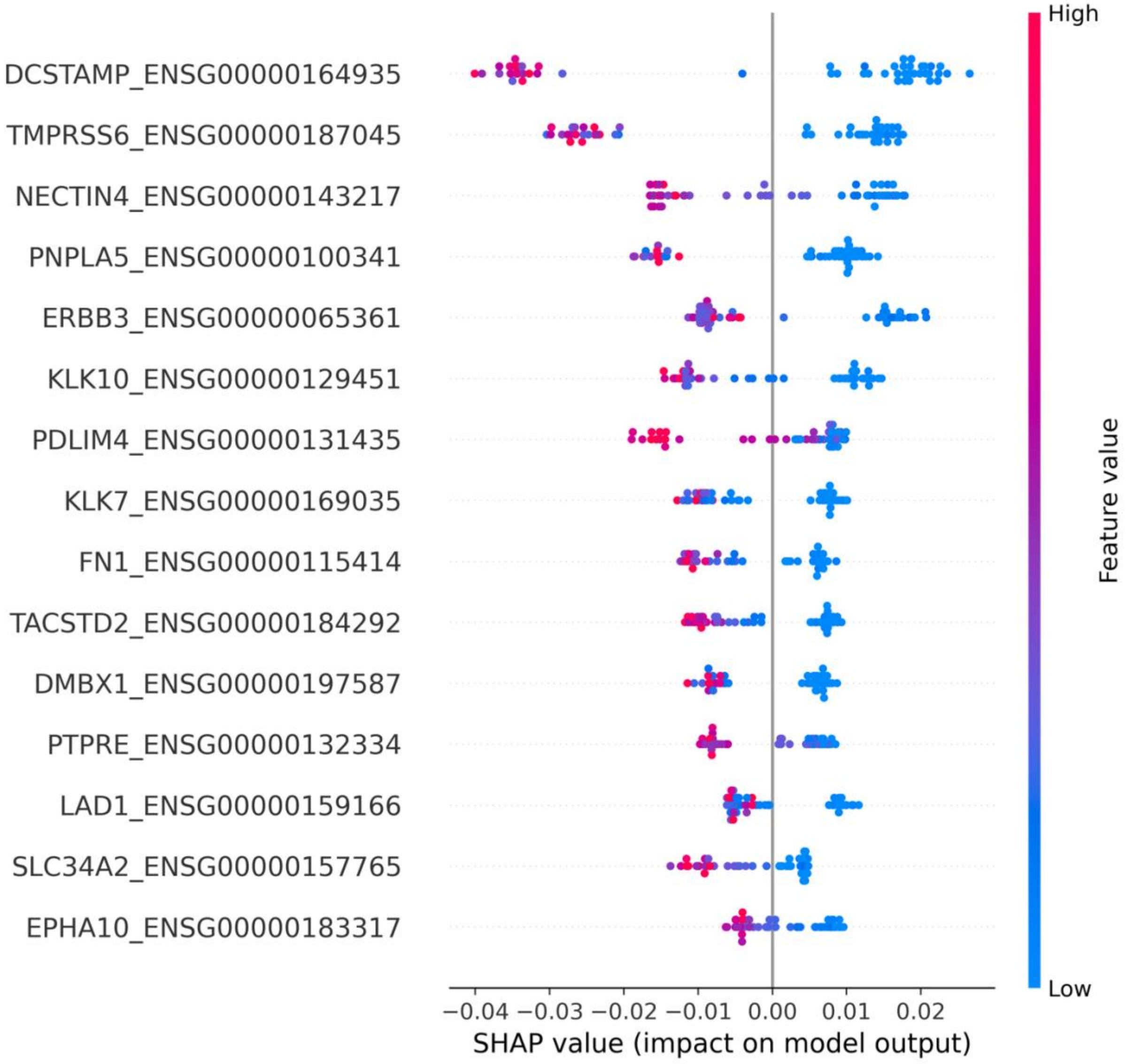

Figure S18. SHAP importance of top 15 features contributing to classification of samples based on *BRAF* mutations

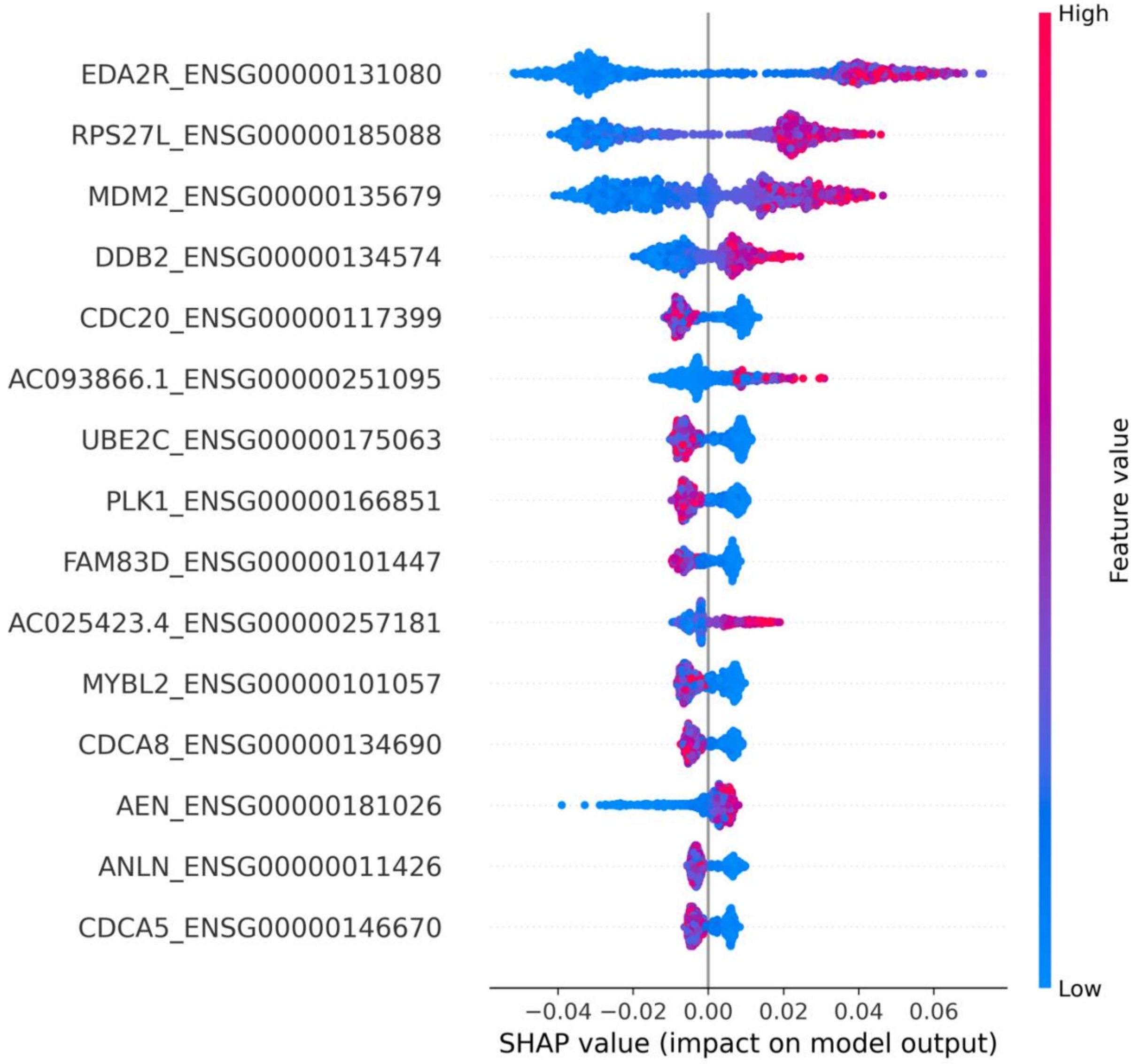

Figure S19. SHAP importance of top 15 features contributing to classification of samples based on *TP53* mutations

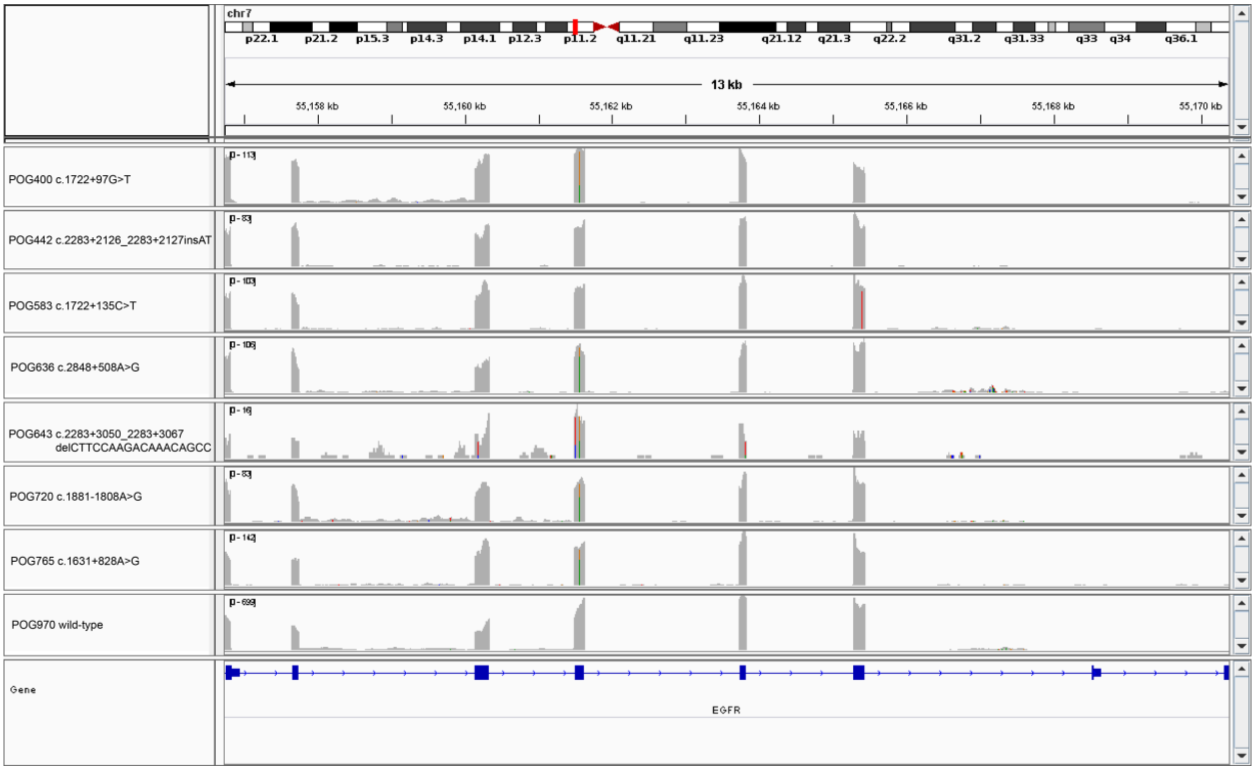

Figure S20. Examples of samples with EFGR intron variants that were predicted as having impactful mutations by the RF model

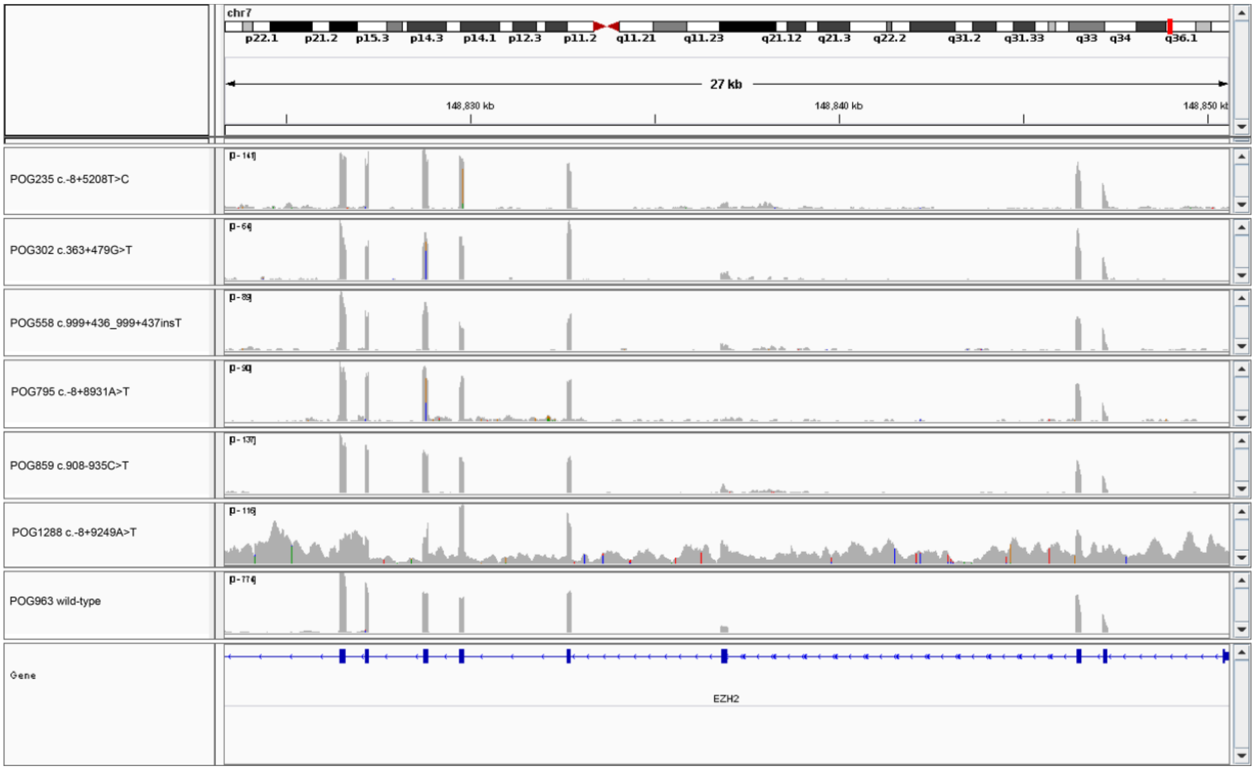

Figure S21. Examples of samples with EZH2 intron variants that were predicted as having impactful mutations by the RF model

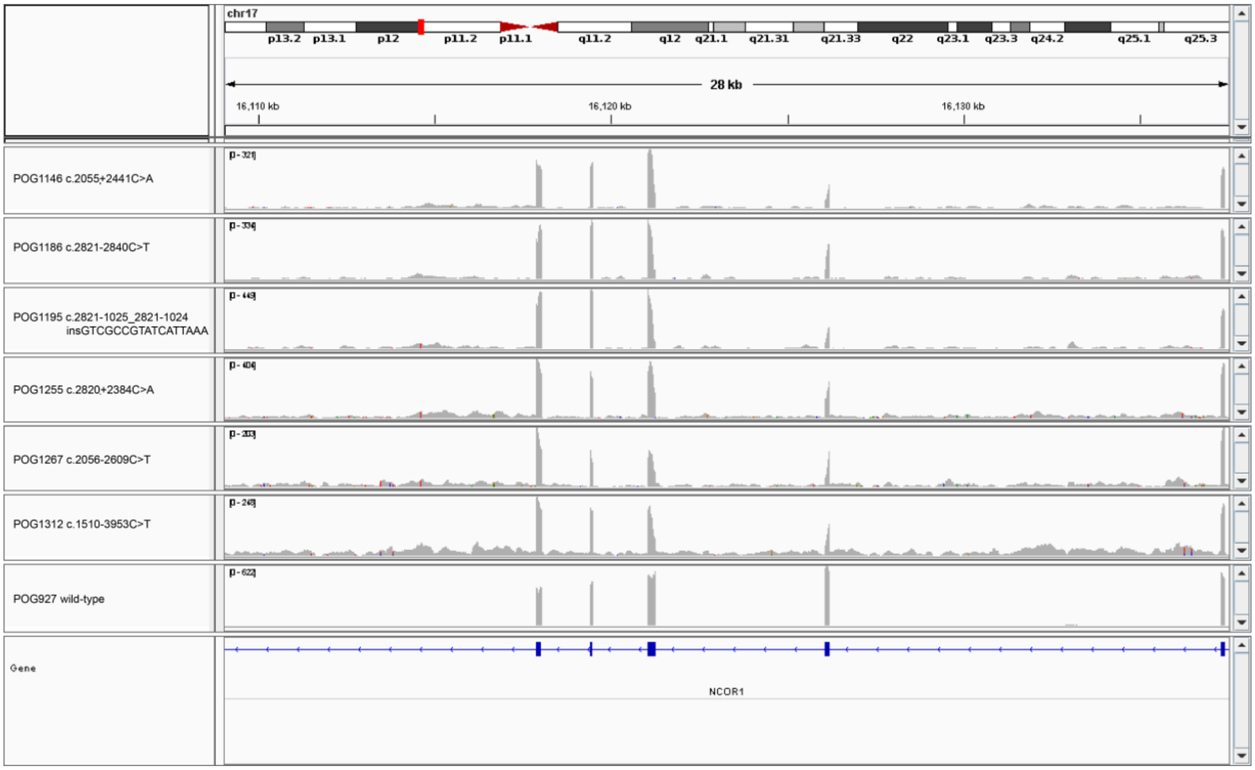

Figure S22. Examples of samples with NCOR1 intron variants that were predicted as having impactful mutations by the RF model

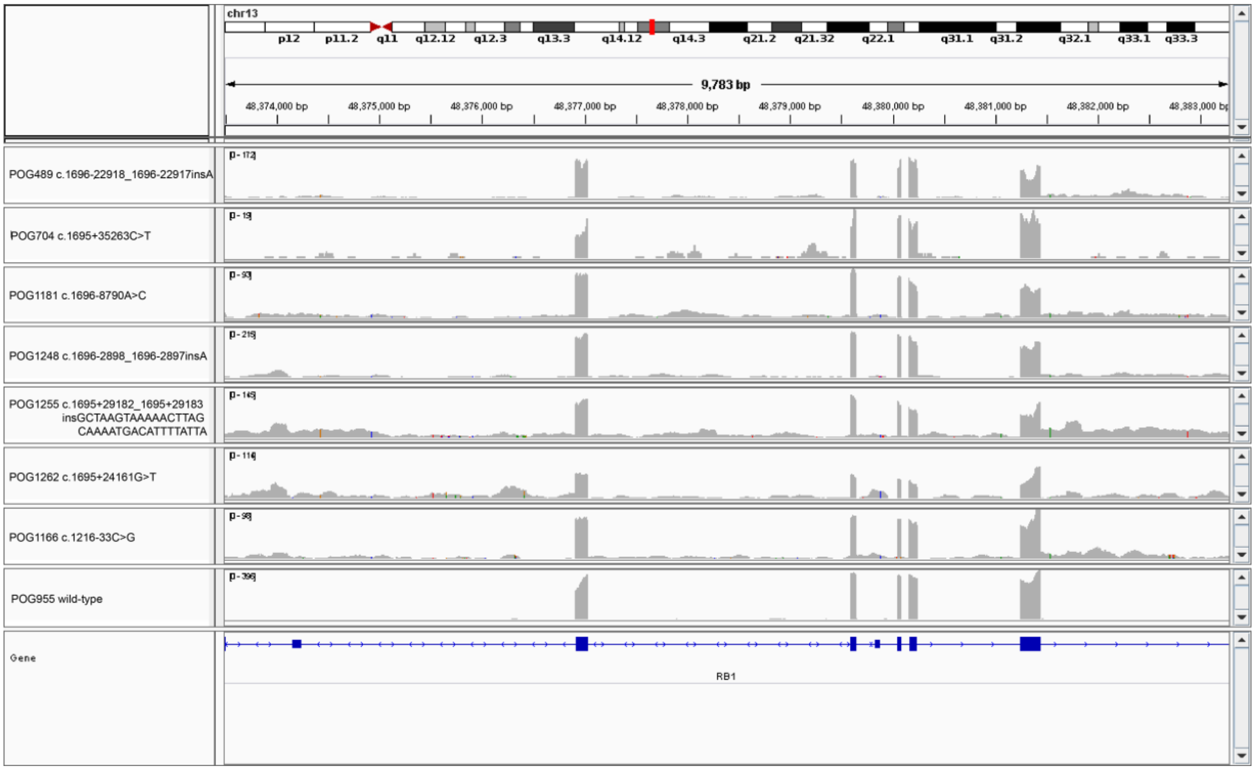

Figure S23. Examples of samples with RB1 intron variants that were predicted as having impactful mutations by the RF model
